# Systematic Approach to Parametrization of Disaccharides for the Martini 3 Coarse-Grained Force Field

**DOI:** 10.1021/acs.jcim.4c01874

**Published:** 2025-01-17

**Authors:** Astrid F. Brandner, Iain P. S. Smith, Siewert J. Marrink, Paulo C. T. Souza, Syma Khalid

**Affiliations:** † Department of Biochemistry, University of Oxford, Oxford OX1 3QU, U.K.; ‡ Groningen Biomolecular Sciences and Biotechnology Institute, University of Groningen, Nijenborgh 7, 9747 AG Groningen, The Netherlands; § Laboratoire de Biologie et Modélisation de la Cellule, CNRS, UMR 5239, Inserm, U1293, Université Claude Bernard Lyon 1, Ecole Normale Supérieure de Lyon, 46 Allée d’Italie, 69364 Lyon, France; ∥ Centre Blaise Pascal de Simulation et de Modélisation Numérique, Ecole Normale Supérieure de Lyon, 46 Allée d’Italie, 69364 Lyon, France

## Abstract

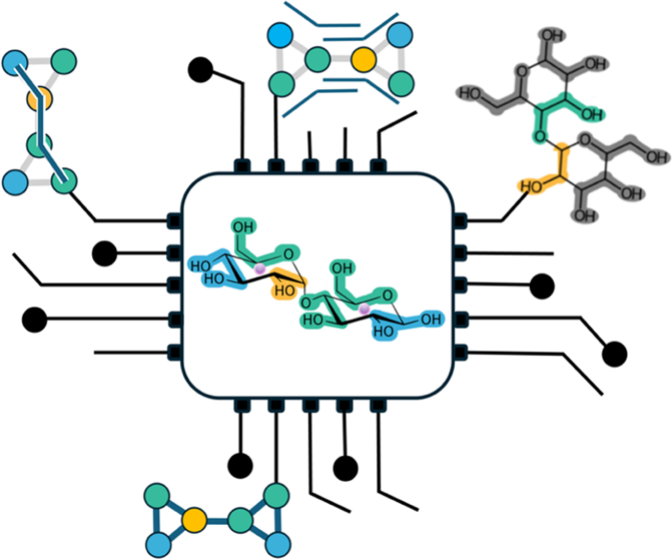

Sugars are ubiquitous in biology; they occur in all kingdoms of life. Despite their prevalence, they have often been somewhat neglected in studies of structure–dynamics–function relationships of macromolecules to which they are attached, with the exception of nucleic acids. This is largely due to the inherent difficulties of not only studying the conformational dynamics of sugars using experimental methods but indeed also resolving their static structures. Molecular dynamics (MD) simulations offer a route to the prediction of conformational ensembles and the time-dependent behavior of sugars and glycosylated macromolecules. However, at the all-atom level of detail, MD simulations are often too computationally demanding to allow a systematic investigation of molecular interactions in systems of interest. To overcome this, large scale simulations of complex biological systems have profited from advances in coarse-grained (CG) simulations. Perhaps the most widely used CG force field for biomolecular simulations is Martini. Here, we present a parameter set for glucose- and mannose-based disaccharides for Martini 3. The generation of the CG parameters from atomistic trajectories is automated as fully as possible, and where not possible, we provide details of the protocol used for manual intervention.

## 1 Introduction

Sugars, from simple monosaccharides to longer, branched oligosaccharides, are ubiquitous in biology. They are present in the headgroups of some lipids, as post-translational modifications to proteins, the backbone of nucleic acids, in the bacterial cell walls, and as individual saccharides in solution in many cellular and extracellular environments. Whether present by themselves or as part of glycosylated macromolecules, sugars serve a range of functional roles (e.g., signaling pathways,^1,2^ formation of binding sites,^3,4^ stabilization of proteins,^3,5,6^ and as osmolytes^7^); indeed many of their roles are yet to be determined.^8^ Inherent difficulties of resolving the structures of sugars attached to proteins have often resulted in their neglect of structure–function relationships. For example, during the recent pandemic, the first structures of the spike protein of the SARS-Cov2 virus (essential for viral-host membrane fusion and design of antiviral therapeutics) did not resolve the glycans; the latter were identified by mass spectrometry^9^ and then modeled by computational approaches.^10−12^ The crucial role played by these glycans was initially predicted by molecular dynamics (MD) simulations; only later did they show up in structural studies.

MD simulations are an established tool within the repertoire of chemists/molecular biochemists for the study of the time evolution of biomolecular systems. Frustratingly, the inherently computationally demanding nature of the algorithms does limit the time and length scales of the simulations such that they can often fall short of accessing phenomena of interest. These limitations may be overcome (in part) by algorithmic advancements, reducing the resolution of the models, or a combination of both philosophies.^13−16^ Reducing the resolution by sacrificing all-atom resolution for more coarse-grained (CG) models offers speed up in three ways: first, there are fewer calculations per integration time step; second, longer integration timesteps may be used given the particles are heavier than atoms; and third, owing to the smoother potential energy landscapes, the kinetics are faster. Perhaps the most widely used CG force field for biomolecular simulations is Martini.^17^ Many carbohydrate systems were parametrized for Martini 2.^18−23^ However, there were some issues of overaggregation^24^ and difficulties in combining different monosaccharides into longer glycans. The latest version of this force field, Martini 3,^25^ incorporates parameter sets for a number of lipids, proteins, and, more recently, also some sugars.^26^ However, sugar parameters have only been reported for a number of monosaccharides or for some complex glycan-based systems such as some glycolipids,^26−28^ cellulose,^29−31^ and lipopolysaccharide.^32,33^ At the time of this work, only three disaccharide models are available.^26^ Here, we report the first set of transferable parameters for glucose- and mannose-based disaccharides within the framework of Martini 3, which have been developed using a systematic approach that is automated as far as possible. This approach was chosen as an attempt to maintain consistency in terms of the parametrization of saccharides, namely, in creating the building blocks for a general parametrization of more complex saccharides. In total, we present parameters for 42 disaccharides, including details of the parametrization protocol. All of the bonded parameters were derived from reference atomistic trajectories, whereas the nonbonded parameters (bead type assignment) and mapping correspond to the already validated Martini 3 monosaccharides. 70% of the systematically built disaccharides were parametrized via a fully automated approach. Where full automation is not currently possible, we provide details of the manual parametrization protocol.

## 2 Material and Methods

### 2.1 All-Atom Simulations

Atomistic coordinates and parameters for all possible glycosidic linkages within mannose and glucose disaccharides were generated in LEaP using the GLYCAM-06j force field.^34^ The sugars were modeled initially in chair conformation.

The reference simulations were performed with the AMBER 20 suite (pmemd).^35^ A simple equilibration protocol was chosen with a maximum of 2000 steps of steepest descent energy minimization, followed by 20 ps of NVT and 5 ns of NPT equilibration at 300 K. A Langevin thermostat with collision frequency γ of 1 ps^–1^ was used to control the temperature. The production run was set to 1 μs molecular dynamics. The reference pressure was set to 1 bar, while the cutoff for nonbonded interactions was set to 1.1 nm. Bonds between hydrogen and heavy atoms were constrained with the SHAKE^36^ algorithm, enabling a 2 fs integration time step (d*t*). Three replicas were run for an example subset of disaccharides, which revealed close similarities in the distributions sampled within the replicas (Supporting Figures S1 and S2). All of the glucose–glucose disaccharides between either only α or β monomers were run for 1 μs also with the CHARMM36 force field in GROMACS^37,38^ to compare the bonded distributions between these popular force fields (Supporting Table S1, Figures S6 and S7). The protocol was the same as described above, except that a cutoff of 1.2 nm was used for short-range nonbonded interactions in the molecular dynamic simulation, and the LINCS^39^ algorithm was used to constrain bonds between hydrogen and heavy atoms. The Nosé–Hoover thermostat (time constant = 1 ps) was used with a Parrinello–Rahman barostat (time constant = 5.0 ps, compressibility = 4.5 × 10^–5^ bar^–1^).

### 2.2 Coarse-Grained Simulations

Coarse-grained simulations were run in GROMACS v2021.4^36,37^ with the Martini 3 force field^25^ and the newly obtained parameters in this work.

A simple protocol was used to test the newly obtained coarse-grained parameters for disaccharides: 500 steps of steepest descent minimization followed by 100 ns equilibration in the NVT ensemble and a 1 μs NPT production run (*T* = 300 K; pressure = 1 bar). The LINCS^39^ algorithm was used to constrain bonds in the ring moieties, enabling the use of a 20 fs time step unless otherwise stated. Electrostatic interactions were treated using the reaction-field approach with a cutoff of 1.1 nm and a dielectric constant of 15.^40^ The same cutoff was used for van der Waals interactions with the implementation of a shift function for the potential.

A softer equilibration protocol was required for some systems, for which flexible bonds were used within the ring moieties during the first steps of equilibration. Specifically, these systems were first energy minimized for 500 steps with steepest descent and flexible bonds, followed by three equilibration steps in NVT at 300 K: 25 ns with a time step of 10 fs, 50 ns with a time step of 20 fs, and 50 ns with a time step of 20 fs and constrained bonds in the ring moieties. The protocols for the production run were as previously described.

In some cases, alongside the distributions of either bonded terms or solvent-accessible surface area (SASA) for analysis, an overlap value in the graphs is reported to quantify the agreement among the distributions. An overlap value between two distributions is computed by summing up the minimum *y*-value between both histograms in each bin for all bins. The reference overlap corresponds to the fraction of the reference distribution (e.g., GLYCAM-06j or Martini 3 original) that was sampled by the new Martini 3 simulation. Values of “full” overlap without the need to define a reference system are reported alongside in Supporting Tables (i.e., 0 = no overlap between the 2 distributions; 1 = perfect overlap between 2 distributions).

### 2.3 Aggregation Tests

**Figure 1:**
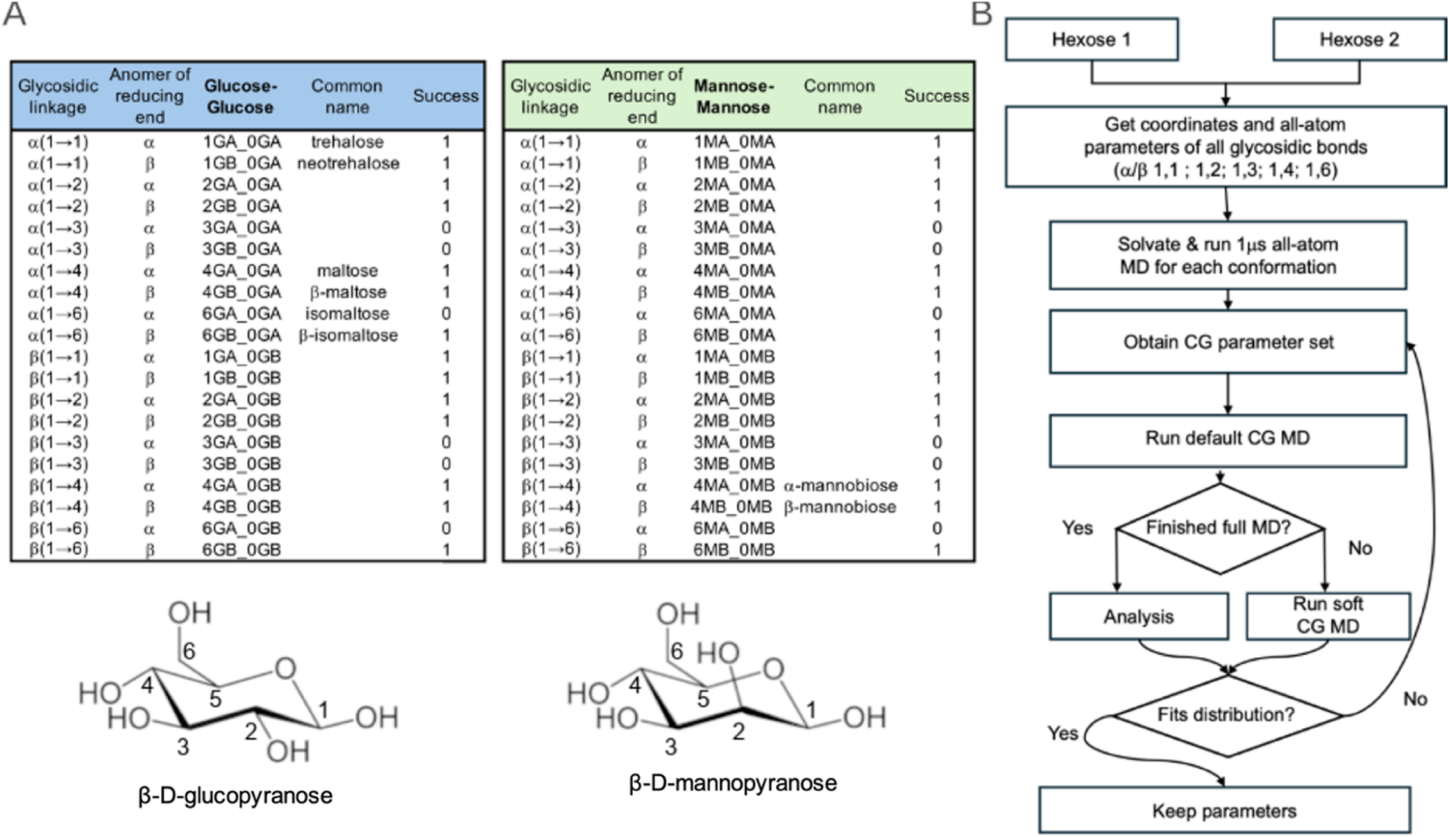
Parameterized disaccharide set and overall workflow. (A) List of all simulated combinations of glucose/mannose containing disaccharides, including the glycosidic bond information, anomeric state of the reducing end, and GLYCAM-based naming scheme. The last column shows the success rate in the automated pipeline (1 = successful, 0 = manual intervention was needed). At the bottom, an example two-dimensional (2D) depiction for β-d-glucopyranose/mannopyranose is shown with the carbon numbering to aid the reader. (B) Parametrization workflow, starting from the choice of two monosaccharides through the extraction of the final coarse-grained parameters after simulation and analyses.

Simulations of the aggregation of 8 of the disaccharides parametrized within this work were performed at the all-atom (GLYCAM-06j,^34^ CHARMM36^41,42^) and coarse-grained resolutions. Independent systems were constructed, and each comprised several copies of only one of the following disaccharides: 1GA_0GA, 1GB_0GB, 2MA_0MA, 3GB_0GB, 4MA_0MA, 6GB_0GB, 6MA_0MB, and 6MB_0MB (following the naming scheme provided in Figure 1). Of these, two disaccharides were chosen, for which the bonded term distributions fit well with atomistic data (1GA_0GA, 1GB_0GB), two for which the distributions deviated to an intermediate extent (2MA_0MA, 4MA_0MA), and two for which the distribution deviated to a slightly larger extent (6GB_0GB, 6MB_0MB). The Martini 3 parameters for all six of these disaccharides were generated by using the fully automated procedure. A further two disaccharides were chosen, for which the parameters were generated using the semiautomated procedure; of these, one disaccharide (3GB_0GB) fit well with atomistic distributions, and the other (6MA_0MB) deviated to a slightly larger extent. Each system was simulated in triplicate at both all-atom and coarse-grain resolutions. All simulations were prepared using the all-atom model of each disaccharide, with each replica containing 20 disaccharide molecules at a concentration of 50 g L^–1^, chosen to match the value used in a previous study of disaccharide aggregation in simulation.^43^ A previous experimental study reported that trehalose was soluble in water at concentrations up to 689 g L^–1^,^44^ and so the disaccharides presented in this work should be readily soluble at 50 g L^–1^. To ensure that the disaccharide concentration was comparable between all-atom and coarse-grained systems, the required ratio of disaccharides to water molecules was computed using the molecular weights of water and trehalose. This calculation indicated that to achieve a disaccharide concentration of 50 g L^–1^, a water box containing 7609 all-atom water molecules would require 20 disaccharides. Using the standard 4:1 mapping for all-atom water molecules to Martini water beads, we therefore solvated the coarse-grained systems with 1902 water beads.

To generate the initial all-atom coordinates, 20 disaccharides were randomly inserted into a cubic box with dimensions (61.45 × 61.45 × 61.45) of Å^3^ containing 7609 water molecules. The resulting all-atom disaccharide structures were mapped into the corresponding coarse-grained representation using our previously reported PyCGTool methodology^45^ to set up the starting configurations for the coarse-grained simulations. Each of the coarse-grained disaccharide systems was then solvated via the insertion of 1902 water beads. The aforementioned “soft” equilibration protocol was used for the systems containing the 2MA_0MA, 4MA_0MA, and 6MB_0MB disaccharides, with the remaining disaccharides treated using the simple protocol described previously. The final AA trajectories were then mapped into pseudo-CG trajectories using PyCGTool in order to standardize analysis across the two regimes. Finally, to compare the aggregation behavior with the Martini 2 model, an analogous coarse-grained system containing CG trehalose was prepared (1GA_0GA), using the already available Martini 2 parameters.^18,24^

### 2.4 Aggregation Behavior

Analysis of disaccharide aggregation was performed on the coarse-grained and pseudo-coarse-grained trajectories with bespoke scripts developed using MDAnalysis v2.7.0^46,47^ and NetworkX v3.3.^48^ For each simulation, network graphs were generated at every frame, with individual disaccharides comprising the nodes and the edges connecting each node defined by the minimum distance between the two disaccharides at the corresponding simulation frame.

A new network was then generated via filtering of the edges, excluding any connections between molecules for which the minimum distance was greater than 4.7 Å. The NetworkX connected components method was then used to obtain sets of disaccharides that remained connected after this filtering process, providing a measure of disaccharide aggregate sizes for each frame of the simulation. This data was then “binned” into aggregate size distributions calculated from frames extracted every 100 ps during the final 500 ns of all replicate simulations of each disaccharide. The explicit time evolution of the frame specific aggregate size distributions was calculated independently across each of the full 1 μs trajectories.

## 3 Results

### 3.1 Parametrization of Disaccharides

Recently, the Martini 3 force field was extended to include parameters for carbohydrates, including 12 monosaccharides and only three disaccharides (sucrose: α-d-glucopyranosyl-(1→2)-β-d-fructofuranoside; β-lactose: β-d-galactopyranosyl-(1→4)-β-d-glucopyranose; trehalose: α-d-glucopyranosyl-(1→1)-α-d-glucopyranoside).^26^ In this section, we present a systematic approach to generate the coordinates of disaccharides and the parameters to use within the Martini 3 force field. We focus on obtaining models for all of the possible disaccharides containing only one of the following hexoses: d-glucopyranose (d-glucose in a pyranose cyclic conformation) or d-mannopyranose (d-mannose in a pyranose cyclic conformation). The only difference between mannose and glucose is the orientation of the −OH group, which is covalently bound to the C2 carbon of the ring.

### 3.2 Fully Automated Parametrization

**Figure 2:**
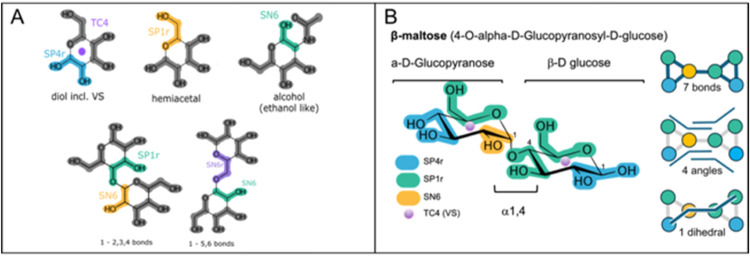
All-atom to Martini 3 mapping strategy. (A) Martini 3 bead types used here are shown mapped onto the chemical structure of the sugars. A proposed general mapping for the bead containing the glycosidic bond in any sugar type is shown for two possible cases in the bottom row (namely, α/β 1–2/3/4 bonds and α/β 1–5/6 bonds). VS refers to “virtual site” bead (adapted from ref (26)). (B) Example of β-maltose mapping (4GB_0GA in Figure 1). On the left, the mapped atoms with their corresponding bead types are shown. Some carbon numbers are shown to aid the reader in the identification of the anomeric carbon and the reducing end. A diagram of the connectivity of the bonded terms is presented on the right, where the lines in blue show the connection between beads for bond lengths (top), angles (middle), and dihedral (bottom). Adapted with permission from ref (26); Copyright 2022 American Chemical Society.

We first describe the fully automated pipeline and resultant CG data sets for mannose and glucose-containing disaccharides (Figure 1). The first step in the automated procedure involves generation of all of the different anomeric conformations of α/β-d-glucopyranose and glycosidic bonds (α/β 1,1; 1,2; 1,3; 1,4; 1,6), resulting in a total of 20 different unique disaccharide molecules. A molecular system containing each unique disaccharide is then set up (automatically) in a box of water and subjected to energy minimization, equilibration, and production MD simulations as per the protocols described in the Section 2. The code then extracts the equilibrium bond lengths, angles, and dihedrals as well as their associated force constants from the resulting atomistic data using our previously reported PyCGTool package.^45^ Concurrently, an all-atom to CG mapping protocol is applied (Figure 2), which is based on the standard Martini 3 rules and in line with the mapping optimized for monosaccharides.^25,26^

A coarse-grained bead will typically be mapped from the center of geometry of four heavy atoms and their bonded hydrogens of a functional group. Figure 2 shows the mapping scheme followed in this work for the chemical moieties present in the hexoses. This mapping strategy derives from previously published Martini 3 carbohydrate parametrization work^26^ with their associated bead type used to model the chemical properties in Martini 3. A particularity of the new Martini 3 model is the use of a virtual site in the middle of the ring moiety (purple sphere in Figure 2). Adding a virtual site (a massless hydrophobic bead) has been shown to help model interactions between cyclic groups (ring stacking). In this version of the disaccharide coarse-graining strategy, we have avoided the 15% expansion in bonds forming part of the ring (which has previously been reported ref (26)) in order to (i) make the comparison to all-atom models more straightforward and (ii) simplify the pipeline.

The CG particle types are then combined with the bonded parameters derived from the all-atom simulations to give a full CG description of the disaccharide. The newly parametrized CG disaccharide is then automatically solvated, the resulting system is energy minimized and equilibrated, and then a production run is performed (see Section 2).

We note here that to model a disaccharide using our procedure, a bead that includes the glycosidic bond is combined into one of the hexose units. This is required to represent the glycosidic link between the monosaccharides and leads to a slightly different mapping compared to the original individual monosaccharides reported by Grünewald et al.^26^ Consequently, the bond terms are also slightly different. We have compared the bonded terms from our trehalose model to that reported in the work of Grünewald et al.^26^ and achieved good agreement (more details are in the Section 3, Supporting Table S3 and Figure S3).

**Figure 3:**
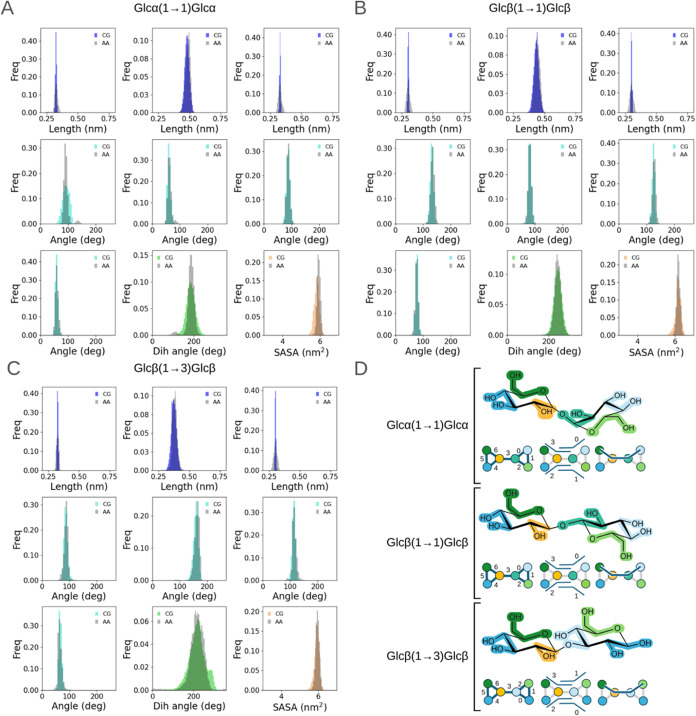
Comparative coarse-grained and all-atom distributions of bonded terms and SASA. Distributions obtained for three example disaccharides with good agreement with atomistic reference data: (A) Glcα(1→1)Glcα, (B) Glcβ(1→1)Glcβ, and (C) Glcβ(1→3)Glcβ (colored: CG, gray: AA) Glcβ(1→3)Glcβ was semiautomatically parametrized, and the other two disaccharides followed the fully automated process. For the sake of space, for each monosaccharide modeled as a ring, only 1 constraint (out of 3) between beads that define a ring is shown (first row of each panel—left and right). The harmonic bond between both monomers is presented (middle top row of each panel). All four defined angles (middle and bottom rows cyan: CG, gray AA), the unique dihedral (green: CG, gray: AA), and SASA (orange: CG, gray: AA) are also shown. (D) 2D depictions of the three disaccharides with their associated indexed bond, angle, and dihedral mappings. The coloring scheme follows the one in Figure 2, using a different hue of either blue or green in each monomer to help identify the components in the cases where one bead type is repeated on a single monomer (the bead type coloring is as follows: blue: SP4r, green: SP1r, yellow: SN6).

The next part of the procedure involves performing a set of standard analyses to assess the new parameter set, including evaluation of distribution of the bond lengths, angles, dihedrals, and solvent-accessible surface area (much of this is automated). Given space constraints, the data for 3 out of the 28 disaccharides that were parametrized using this fully automated procedure are shown in Figure 3, and all of the other data are available in the Supporting Information (SI).

Although here we use the bonded distributions from GLYCAM-06j all-atom simulations to infer the CG bonded parameters, we have decided to include a couple of examples of bonded distributions from CHARMM36, an equally popular all-atom force field for biomolecular simulations (Supporting Table S1, Figures S6 and S7)

### 3.3 Semiautomated Parametrization

In total, from the 40 mannose/mannose and glucose/glucose disaccharides simulated here, 12 required some manual intervention in the parametrization procedure, as the mapping could not be assigned fully in an automated manner. The protocol failed in the mapping procedure for 1–3, as well as some 1–6 linkages glycosidic linkages (annotated with a value of success = 0 in Figure 1). The failure occurred in the generation of the AA to CG mapping files directly after the all-atom trajectory analysis. Consequently, in these cases, the mapping files were generated manually. For the 1–3 linkages, some atoms were not automatically mapped, principally as a consequence of the graph growing in the wrong direction along the ring atoms after the assignment of the glycosidic bead. In the 1–6 cases where there were errors, these arose due to some atoms being wrongly assigned in the glycosidic region, resulting in a “split” unrealistic bead. Both of these scenarios are potentially the consequence of the mapping being based on the Amber atom naming scheme.

For these cases, it is recommended to use the manual assignment procedure, as shown in Figure 2. The mapped structure is then combined with the normal workflow as per the fully automated procedure to extract the bonded parameters from the atomistic simulations. The resulting coordinates and parameter set are then subjected to one of the equilibration procedures, as previously mentioned.

### 3.4 Analyses

While the full analyses for all disaccharides we have parametrized here are provided in the Supporting Information, here we focus on six cases, three of which give clear agreement with atomistic data and three of which differ slightly from the atomistic data. For each of the three disaccharides in their respective categories, two were parametrized via a fully automated approach, and one followed the semiautomated parametrization. The following metrics are discussed on a per disaccharide basis: distributions of bond lengths, angles, dihedral angles, and solvent-accessible surface area (SASA).

The full list of these six disaccharides and the extent of their discrepancy from reference atomistic data is shown in Table 1, ordered by categories (i.e., three disaccharides with excellent agreement, Glcα(1→1)Glcα, Glcβ(1→1)Glcβ, and Glcβ(1→3)Glcβ and three with slight disagreement, Glcβ(1→6)Glcβ, Manβ(1→6)Manβ, and Manβ(1→6)Manα, with atomistic distributions). In all of the modeled disaccharides, bond 0–2 and bond 3–6 (where the numbering follows bond order in their respective CG topology file) were modeled as constraints and show negligible deviation from atomistic reference data as a consequence of being a constraint.

**Table 1:** Bond Lengths, Angles, Dihedral Angles, and SASA for Six Example Cases^a^

disaccharide	resolution	bond0	bond1	bond2	bond3	bond4	bond5	bond6
Glcα(1→1)Glcα	CG	0.32 ± 0.00	0.36 ± 0.00	0.45 ± 0.00	0.48 ± 0.02	0.33 ± 0.00	0.36 ± 0.00	0.47 ± 0.00
	AA	0.32 ± 0.02	0.36 ± 0.02	0.45 ± 0.45	0.49 ± 0.02	0.33 ± 0.01	0.36 ± 0.01	0.47 ± 0.01
	% Δ(AA – CG)	0%	0%	0%	2.08%	0%	0%	0%
Glcβ(1→1)Glcβ	CG	0.3 ± 0.00	0.37 ± 0.00	0.45 ± 0.00	0.43 ± 0.02	0.30 ± 0.00	0.38 ± 0.00	0.47 ± 0.00
	AA	0.3 ± 0.01	0.37 ± 0.01	0.45 ± 0.01	0.44 ± 0.02	0.30 ± 0.01	0.38 ± 0.01	0.47 ± 0.01
	% Δ(AA – CG)	0%	0%	0%	2.33%	0%	0%	0%
Glcβ(1→3)Glcβ	CG	0.33 ± 0.00	0.45 ± 0.00	0.35 ± 0.00	0.37 ± 0.02	0.3 ± 0.00	0.37 ± 0.00	0.47 ± 0.00
	AA	0.33 ± 0.01	0.45 ± 0.01	0.35 ± 0.01	0.38 ± 0.02	0.3 ± 0.01	0.37 ± 0.02	0.47 ± 0.01
	% Δ(AA – CG)	0%	0%	0%	2.7%	0%	0%	0%
Glcβ(1→6)Glcβ	CG	0.43 ± 0.00	0.32 ± 0.00	0.35 ± 0.00	0.37 ± 0.02	0.3 ± 0.00	0.37 ± 0.00	0.47 ± 0.00
	AA	0.43 ± 0.01	0.32 ± 0.01	0.35 ± 0.01	0.38 ± 0.02	0.3 ± 0.01	0.37 ± 0.01	0.47 ± 0.01
	% Δ(AA – CG)	0%	0%	0%	2.7%	0%	0%	0%
Manβ(1→6)Manα	CG	0.48 ± 0.00	0.32 ± 0.00	0.42 ± 0.00	0.31 ± 0.02	0.29 ± 0.00	0.37 ± 0.00	0.44 ± 0.00
	AA	0.48 ± 0.02	0.32 ± 0.01	0.42 ± 0.02	0.31 ± 0.02	0.29 ± 0.01	0.37 ± 0.01	0.44 ± 0.02
	% Δ(AA – CG)	0%	0%	0%	0%	0%	0%	0%
Manβ(1→6)Manβ	CG	0.41 ± 0.00	0.31 ± 0.00	0.35 ± 0.00	0.37 ± 0.02	0.29 ± 0.00	0.37 ± 0.00	0.44 ± 0.00
	AA	0.41 ± 0.01	0.31 ± 0.01	0.35 ± 0.01	0.38 ± 0.02	0.29 ± 0.01	0.37 ± 0.01	0.44 ± 0.02
	% Δ(AA – CG)	0%	0%	0%	2.7%	0%	0%	0%

disaccharide	resolution	angle0	angle1	angle2	angle3	dihedral	SASA
Glcα(1→1)Glcα	CG	92.13 ± 11.87	60.75 ± 5.37	87.72 ± 6.26	58.31 ± 4.27	185.85 ± 20.99	5.83 ± 0.13
	AA	92.77 ± 10.72	62.78 ± 7.20	88.01 ± 5.87	59.22 ± 5.25	185.09 ± 20.34	5.94 ± 0.09
	% Δ(AA – CG)	0.69%	3.34%	0.33%	1.56%	–0.41%	1.89%
Glcβ(1→1)Glcβ	CG	131.58 ± 5.82	81.95 ± 5.55	124.99 ± 4.91	77.18 ± 4.78	244.00 ± 18.59	6.17 ± 0.15
	AA	133.64 ± 7.05	81.55 ± 6.08	127.27 ± 5.95	76.83 ± 5.30	243.01 ± 18.77	6.18 ± 0.09
	% Δ(AA – CG)	1.57%	–0.49%	1.82%	–0.45%	–0.41%	0.16%
Glcβ(1→3)Glcβ	CG	86.93 ± 6.70	158.69 ± 8.45	112.99 ± 5.82	66.32 ± 5.19	218.19 ± 39.55	5.92 ± 0.13
	AA	87.6 ± 7.62	161.78 ± 10.70	115.41 ± 7.79	65.09 ± 7.39	215.86 ± 36.39	5.97 ± 0.10
	% Δ(AA – CG)	0.77%	1.95%	2.14%	–1.85%	–1.07%	0.84%
Glcβ(1→6)Glcβ	CG	125.73 ± 17.77	115.2 ± 22.67	126.71 ± 5.44	78.84 ± 5.12	142.28 ± 111.87	5.9 ± 0.21
	AA	121.97 ± 17.47	114.85 ± 26.51	127.85 ± 6.68	77.02 ± 6.01	144.34 ± 63.58	6.1 ± 0.16
	% Δ(AA – CG)	–2.99%	–0.3%	0.9%	–2.31%	1.45%	3.39%
Manβ(1→6)Manα	CG	107.75 ± 14.10	97.62 ± 18.15	121.85 ± 5.29	74.68 ± 5.67	138.4 ± 46.12	5.9 ± 0.18
	AA	109.64 ± 14.30	103.41 ± 18.19	121.19 ± 5.47	71.47 ± 7.07	135.80 ± 39.13	5.96 ± 0.16
	% Δ(AA – CG)	1.75%	5.93%	–0.54%	–4.3%	–1.88%	1.02%
Manβ(1→6)Manβ	CG	110.09 ± 18.31	106.21 ± 20.30	133.34 ± 5.78	84.35 ± 6.89	133.26 ± 73.56	5.88 ± 0.22
	AA	112.28 ± 18.27	110.06 ± 21.34	133.75 ± 6.24	84.33 ± 8.12	132.14 ± 50.44	6.02 ± 0.13
	% Δ(AA – CG)	1.99%	3.62%	0.31%	–0.02%	–0.84%	2.38%

a Averages over the last 500 ns of each disaccharide trajectory and their associated standard deviations are reported. The percentage difference between the means calculated in equivalent all-atom and coarse-grained models is also provided. The three best-performing disaccharides (top) are separated from those that show slight disagreement to atomistic data (bottom) by a horizontal double line through the center of each table. The numbering for bonds and angles follows the order present in the CG topology file.

The disaccharide Glcα(1→1)Glcα (Figure 3A) that was parametrized in a fully automated manner yielded very good agreement with the all-atom reference simulation. The location of the maxima in each bonded term distribution of the coarse-grained model aligned well with the equivalent maxima obtained from the reference all-atom model. The difference in the mean of the all-atom and coarse-grained distributions spanned values from as little as 0.4% to a maximum of 3.4% for a one angle distribution. Extremely small populations present in the atomistic reference for the angle distributions are lost due to the coarse-graining process; however, they are neglectable in the CG framework. The use of constraints for the cycles instead of bonds effectively creates an extremely narrow distribution compared to the unconstrained equivalent atomistic bond; however, the maxima of those distributions matched well, and more importantly, the unconstrained bond between both monosaccharide moieties (central plot in upper row for each disaccharide distribution) has the same width as the one present in the all-atom simulation, showing excellent agreement. The dihedral distributions also matched well; however, an extremely small population is lost as the automated parametrization only checks for a normally distributed population and does not account for multimodality. Glcβ(1→1)Glcβ was also fully automatically parametrized and showed good agreement with atomistic reference data, as shown in Figure 3B.

Glcβ(1→3)Glcβ (Figure 3C) required semiautomated parametrization as follows: the all-atom to coarse-grained mapping and connectivity definition file (i.e., which coarse-grained beads will be connected via bonds, angles or dihedrals) had to be manually prepared following the Martini 3 suggested rules.

We decided to compare the results for the disaccharide Glcα(1→1)Glcα (trehalose) with the previously published Martini 3 trehalose parameters^26^ (Supporting Table S3, Figures S3 and S4). The modeled constraints (i.e., bonds that define a cycle) display a systematic shift between 0.02 and 0.03 nm toward larger values for the original Martini 3 parameters with respect to the new parameters due to the bond length scaling for the bonds that define the rings. The bond between both monomers also shows a clear shift between both models, with a ∼0.06 nm larger average value for the new Martini 3 with respect to the original one. Nonetheless, there was excellent agreement for the dihedral distribution (with a reference overlap of 0.93) and good agreement for all of the angle distributions (spanning overlap values of 0.63–0.88). Finally, the SASA distribution showed good agreement, with an overlap reference value of 0.6. Furthermore, we decided to compare the distributions of Glcα(1→1)Glcα and Glcα(1→1)Glcβ to assess the effect of the orientation of the OH group on the anomeric carbon. It is clear that in this case, where the glycosidic bond is established between two C1 atoms, the coarse-grained model is sensitive enough to discriminate between anomers, showing clear differences in the distributions of angles, the glycosidic bond, and the dihedral (Supporting Table S3, Figure S5).

**Figure 4:**
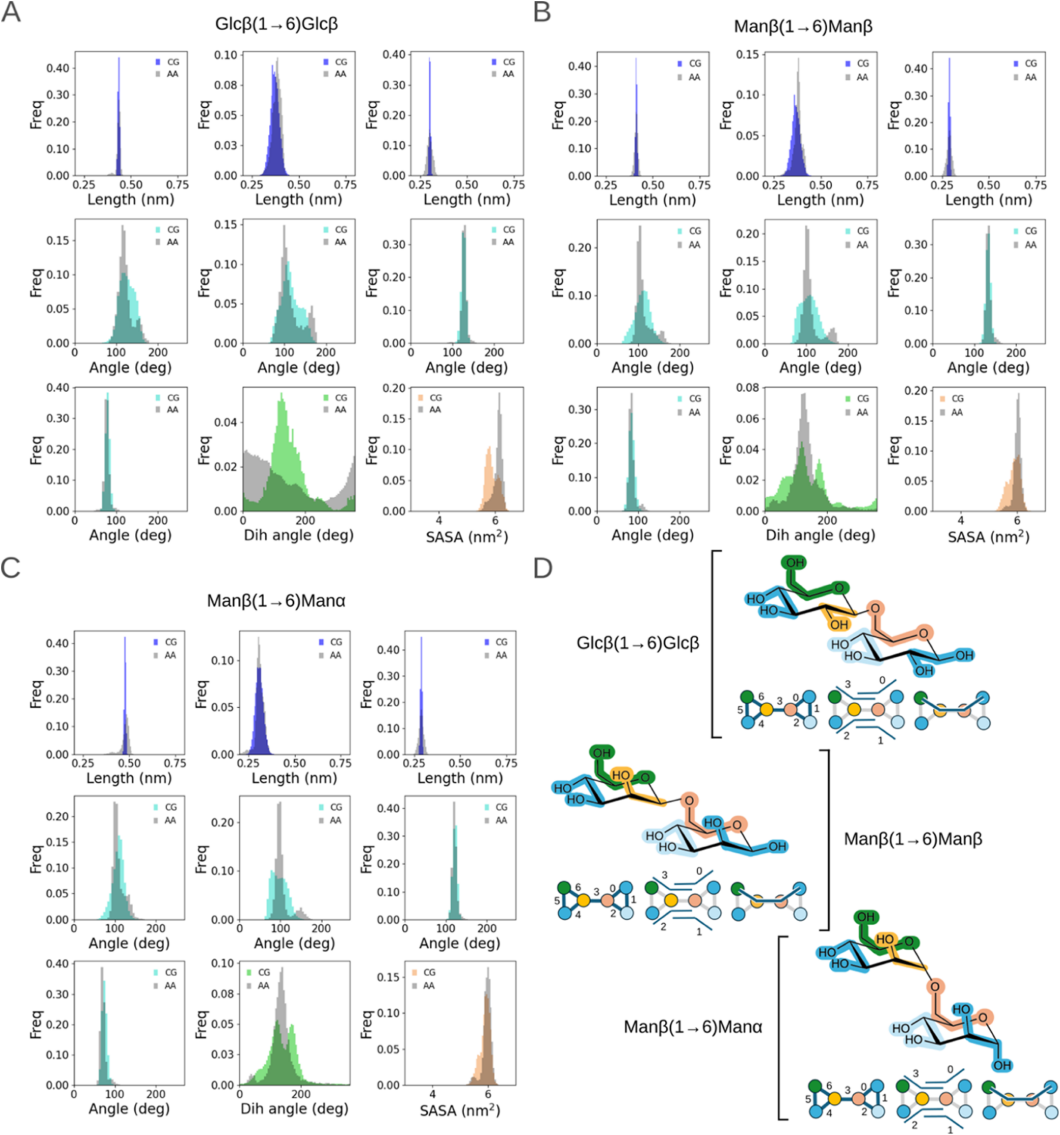
Comparative coarse-grained and all-atom distributions of bonded terms and SASA. Distributions obtained for three example disaccharides that show discrepancy with atomistic reference data: (A) Glcβ(1→6)Glcβ, (B) Manβ(1→6)Manβ, and (C) Manβ(1→6)Manα (colored: CG, gray: AA). Manβ(1→6)Manα was semiautomatically parametrized, and the other two disaccharides followed the fully automated process. For the sake of space, for each monosaccharide modeled as a ring, only 1 constraint (out of 3) between beads that define a ring is shown (first row of each panel—left and right). The harmonic bond between both monomers is presented (middle top row of each panel). All of the four defined angles (middle and bottom rows cyan: CG, gray AA), the unique dihedral (green: CG, gray: AA), and SASA (orange: CG, gray: AA) are plotted, too. (D) 2D depictions of the three disaccharides with their associated indexed bond, angle, and dihedral mappings. The coloring scheme follows the one in Figure 2, using a different hue of either blue or green in each monomer to help identify the components in the cases where one bead type is repeated on a single monomer (the bead type coloring is as follows: blue: SP4r, green: SP1r, yellow: SN6, pink: SN6r).

We next sought to analyze three cases where the quality of the fit showed greater discrepancy from the atomistic simulations for the properties analyzed here: Glcβ(1→6)Glcβ, Manβ(1→6)Manβ, and Manβ(1→6)Manα (Figure 4). The first two disaccharides were parametrized using a fully automated procedure, whereas the third required a semiautomated approach, as previously stated. For all of these three cases, the constraints and bonds are well modeled with the new coarse-grained parameters and give excellent agreement with the atomistic reference distributions (always below 3% deviation in the average values, as shown in Table 1). However, when looking at the angle and dihedral angle distributions, in some cases, it is evident that there is a larger dispersion in the distribution profile for the coarse-grained simulations with respect to their atomistic reference, as clearly seen for the first two reported angles of Glcβ(1→6)Glcβ and Manβ(1→6)Manβ in Figure 4. Interestingly, when looking at the difference in the averages for those two angles for each disaccharide, only the second angle of Manβ(1→6)Manβ shows a relative difference of 5.93%, whereas the others span values from 0.3% to a maximum 2.99% deviation. Similarly, for the dihedral angle distribution of Glcβ(1→6)Glcβ, a clearly different profile can be seen for coarse-grained and all-atom data; nonetheless, the difference in the means of both distributions is only 1.45%. This perhaps shows the importance of looking at the distribution profiles alongside mean values for a good qualitative and quantitative comparison.

### 3.5 Aggregation Studies

**Figure 5:**
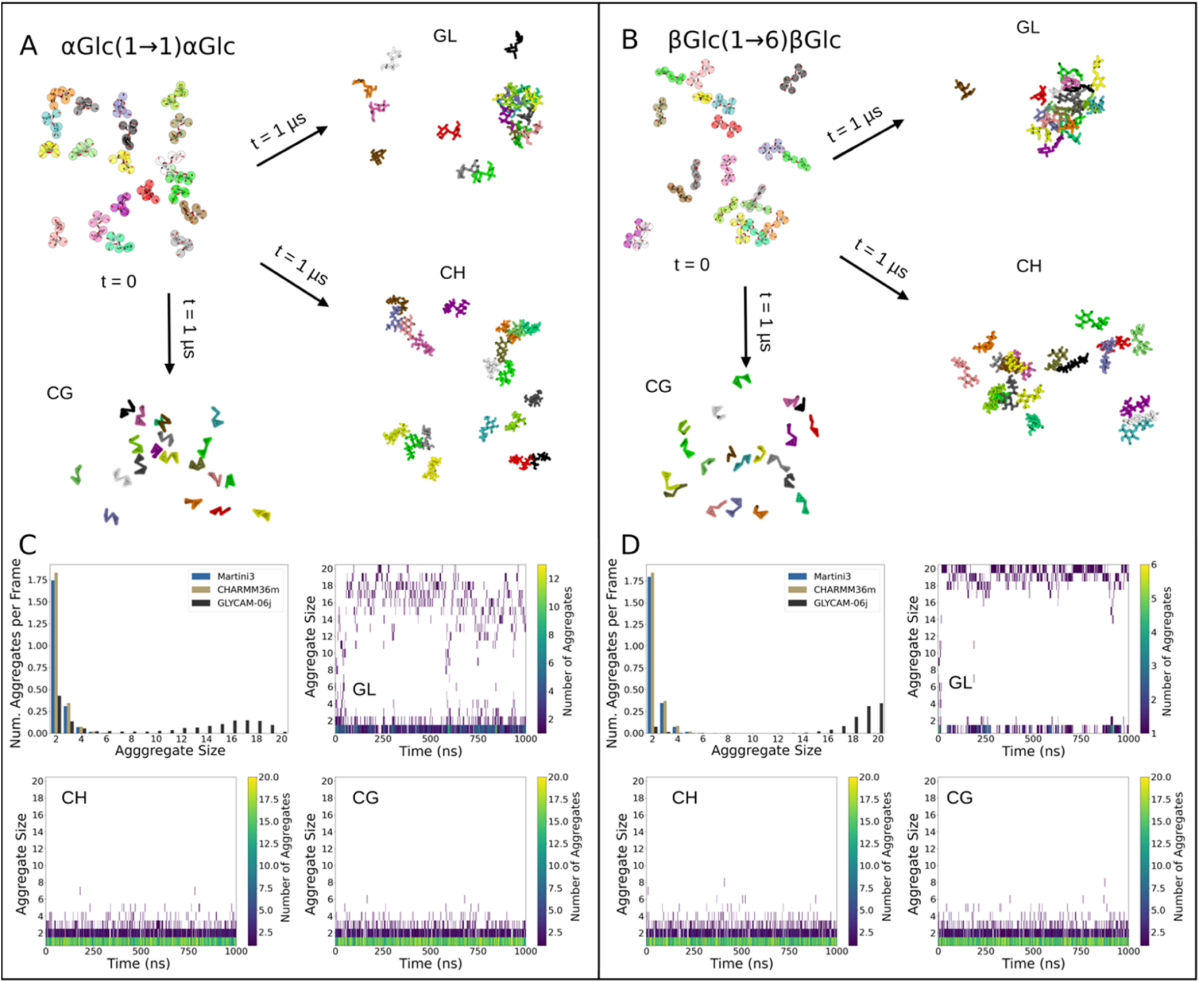
Comparative aggregation behavior in coarse-grained and all-atom simulations for two example systems. The boxes separate the information for the different disaccharides (left: Glcα(1→1)Glcα; right: Glcβ(1→6)Glcβ). (A, B) Molecular images of the initial configuration (*t* = 0) and final frame for the all-atom simulations (GL: GLYCAM-06j; CH: CHARMM36; *t* = 1 μs) and Martini 3 coarse-grained (CG, *t* = 1 μs) simulation. Individual disaccharides are depicted in unique colors. At *t* = 0, the coarse-grained beads are shown as spheres in their mapped positions from the all-atom coordinate representation in sticks. The box edges ∼ (64 × 64 × 64) Å^3^ and water molecules are omitted for clarity. (C, D) Histograms of aggregate sizes observed in the combined last 500 ns of all of the three replicates for either GLYCAM-06j (gray), CHARMM36 (tan), or CG (blue) and the time evolution of the formation of aggregates in one example replica for all-atom simulations (GL, CH) and CG. The Glycam06-j force field simulations of the Glcα(1→1)Glcα, Glcβ(1→1)Glcβ, Manα(1→2)Manα, Glcβ(1→3)Glcβ, Manα(1→4)Manα, Glcβ(1→6)Glcβ, Manβ(1→6)Manβ, and Manβ(1→6)Manα disaccharides were extended to 2 μs as an additional check that the bimodal distributions were not simply a function of simulations not being long enough, the data are presented in Supporting Figures S20–S29. The distributions are essentially unchanged.

Following the results from the parametrization, we investigated the aggregation of eight disaccharides using the new Martini 3 parameters and compared the results to atomistic control simulations performed using the GLYCAM-06j force field. We performed simulations comprising a homogeneous 50 g L^–1^ solution of the disaccharide of interest in water. At this concentration, we expect all of the disaccharides to be readily soluble in water^44^ (see Section 2 for further details). While the initial disaccharide positions were equivalent between each pair of replicate CG and AA simulations, the distribution in disaccharide aggregate sizes varied considerably between the two resolutions. There was a strong preference toward the formation of dimers across all CG disaccharide models, followed by trimers and tetramers. The largest aggregate was observed in one replica of the Glcβ(1→3)Glcβ model, comprising nine individual disaccharides. This unimodal distribution was consistent between disaccharides that were parametrized using either the fully automated or semiautomated protocols, with the second largest aggregates observed in the Manα(1→2)Manα and Glcβ(1→6)Glcβ models, comprising 8 individual disaccharides. In contrast to this, the AA GLYCAM-06j simulations each showed a bimodal distribution in aggregate sizes, with the primary (largest) peak corresponding to aggregate sizes between 18 and 20 and the secondary peak corresponding to aggregate sizes of 2. These data highlight that while disaccharide dimer formation was still present within the AA GLYCAM-06j simulations, each system exhibited a preference toward the formation of larger aggregates comprising most, if not all, disaccharide moieties within the system, contrary to what was expected according to the experimentally derived solubility of disaccharides at this concentration.^44^ For this reason, we decided to perform the same all-atom simulations using CHARMM36. Interestingly, for all of the disaccharides studied in the aggregation assays, Martini 3 showed excellent agreement with the CHARMM36 results for both the average number of aggregates per frame and aggregate dynamics (Figure 5 and Supporting Figures S20–S28). This is in line with the aggregation behavior of trehalose observed in experiments (i.e., soluble at the same concentration).

Moreover, even though there was agreement between the all-atom CHARMM36 aggregation behavior and the Martini 3 coarse-grained simulations, we decided to further compare the final Martini 3 model with its predecessor Martini 2. To this end, we performed three further replica simulations of the Glcα(1→1)Glcα system using the Martini 2 force field, utilizing the standard parameters for trehalose. Similar to the Martini 3 simulations, an unimodal distribution of aggregate sizes was observed in all three replicates, with a peak in aggregate sizes corresponding to disaccharide dimer formation. Notably, however, the largest aggregate observed in the Martini 2 simulations comprised 15 individual disaccharides, indicating that while both Martini force fields did not reproduce the bimodal distribution obtained in the GLYCAM-06j simulation, Martini 2 exhibited a greater tendency toward the formation of large disaccharide aggregates when compared to Martini 3. It has been reported previously that the nonbonded interactions between glycans are too attractive in Martini 2.^24,43^ The solution to this issue was a key target during the development of the Martini 3 force field.^17^ It is therefore reassuring that our results indicate that the disaccharides are readily soluble in both Martini models and that the Martini 3 force field exhibits a reduced extent of carbohydrate aggregation compared to Martini 2.

Further to this, analysis of the time evolution of the aggregate size distributions indicated that in the reference AA GLYCAM-06j simulations, large disaccharide aggregates formed rapidly (<50 ns) at the onset of production MD. The size of these large aggregates fluctuated over time to differing extents for each type of disaccharide, with the Glcβ(1→6)Glcβ system exhibiting the lowest volatility in aggregate size. In contrast, a high density of monomers and dimers was observed throughout the entire duration of each CG simulation, consistent across all disaccharide types, as well as in the CHARMM36 simulations. There was no evident trend toward the formation of larger aggregates throughout the time evolution of any CG simulation or CHARMM36 simulation; thus, the dearth of large aggregates within the CG simulations is likely not a result of insufficient sampling and is instead indicative of lesser aggregation of disaccharides within both the Martini 2 and Martini 3 force fields compared to the overly aggregated GLYCAM-06j simulation.

## 4 Discussion

In this paper, we present a full set of Martini 3 parameters of single disaccharides containing all anomeric forms of Glc–Glc and Man–Man combinations. Importantly, we present a generic strategy for parametrization of bonded terms of disaccharides from atomistic trajectories that is easily transferable to disaccharides composed of any monosaccharides.

To this end, we have used the GLYCAM-06j all-atom force field to simulate a single disaccharide in water and used it as a reference frame to infer the CG bonded parameters. The choice of GLYCAM-06j was primarily due to the simplicity and high flexibility for the user to create initial canonical structures without the need for a PDB structure. However, the choice of a particular force field to use as a reference will logically affect the sampling compared to other force fields in the case of observed differences in behavior. For this reason, and given that a full comparison between all-atom force fields is not the scope of this work, we have also computed the bonded distributions for simulations of a smaller subset of disaccharides run with CHARMM36. Although there are some minor shifts in a few properties, the total average overlap for all of the bonded terms distributions with respect to the reference GLYCAM-06j force field is 66%, from values ranging from 0 to 98% (Supporting Tables S1 and S2, Figures S6 and S7).

When the distributions from the reference atomistic simulations and the CG data are compared, some discrepancies can be observed. Most of the discrepancies here are connected to the bimodal distributions of bonded terms. Symmetrical periodic dihedrals can potentially be better fitted in GROMACS, exploring the different multiplicities, including combinations of proper cosine dihedrals, or by using the Ryckaert–Bellemans function. However, the accurate fitting of nonsymmetrical dihedrals, as presented in Figure 4B, can be challenging. Further refinement could be achieved via tabulated potentials in GROMACS or direct implementation of more complex functional forms directly in codes such as OpenMM.^49^

It could be argued that there is no need to have specific bond, angle, and dihedral terms for each possible combination of monosaccharides forming a disaccharide. When comparing the equilibrium values of bonded terms between equivalent glucose–glucose and glucose–mannose disaccharides (i.e Glcα(1→1)Glcα vs Manα(1→1)Manα; same glycosidic bond and anomeric conformation, where the only difference is the presence of mannose or glucose), 50% of the equilibrium values for the bonded terms of glucose–glucose dimers were within 10% of the equilibrium values from their analogous glucose–glucose dimers. However, the average values deviated by only ∼34% between α or β glycosidic bonds in glucose–glucose disaccharides and by ∼13% between mannose–mannose disaccharides. This opens the door to explore if some terms could be further simplified; however, given that it is not systematic, we kept the obtained values in the parameters presented in this work.

It is noteworthy that our aggregation studies show good agreement between our Martini 3 simulations, CHARMM36 all-atom simulations, and experimental studies but do not agree well with the GLYCAM-06j all-atom data. This could be rationalized by the fact that the aggregation behavior is mainly driven by the nonbonded interactions, and these were defined by the chemistry of moieties that were coarse-grained into one bead (i.e., the mapping of atoms into a CG bead with a specific bead type).

We pose the idea that models of oligosaccharides of higher order could be constructed based on combinations of simpler disaccharide models. Such an approach would avoid ad hoc parametrization of every individual type of oligosaccharide available, given the enormous variability in polysaccharides present in nature, although we note here that a minimal building block that represents a branching point (i.e., a branched trimer) may be required to better model oligo- and polysaccharides. The models we present here can be easily ported for use with POLYPLY^50^ to enable the systematic construction of molecular coordinate chains to further streamline the process of building models for more complex polysaccharides.

## Data Availability

The following data will become freely available for download upon publication of the present manuscript: (1) gro and itp files for all CG disaccharide models (file type: text files), (2) all of the scripts required to reproduce the models (files types: text files in the format of input files to run simulations in GROMACS, Python, and Bash scripts), (3) the all-atom and Martini 3 coarse-grained trajectories from the systems presented in Figures 3 and 4. It can be found at https://zenodo.org/records/14291060.

## Supporting Information Available

Three additional tables and 29 figures showing detailed analyses for all disaccharide CG models developed as part of this work (PDF)
One additional table containing the SMILES for all of the disaccharides and their associated name prefixes (PDF)
