# Supplementary material for: Systematic Approach to Parametrization of Disaccharides for the Martini 3 Coarse-Grained Force Field
Source: J Chem Inf Model. 2025 Jan 17;65(3):1537–48. doi: 10.1021/acs.jcim.4c01874 (PMC11815824; doi:10.1021/acs.jcim.4c01874)
Supplement: Supplementary file 1 — ci4c01874_si_001.pdf [file ci4c01874_si_001.pdf]

Supplementary Information

Systematic Approach to Parametrization of Disaccharides for the Martini 3 Coarse-Grained Force Field.

Astrid F. Brandner[1], Iain P. S. Smith[1], Siewert J. Marrink[2], Paulo C.T. Souza[3,4*], Syma Khalid[1*]*

1.Department of Biochemistry, University of Oxford, Oxford OX1 3QU

2. Groningen Biomolecular Sciences and Biotechnology Institute, University of Groningen, Nijenborgh 7, 9747 AG Groningen, The Netherlands.

3. Laboratoire de Biologie et Modélisation de la Cellule, CNRS, UMR 5239, Inserm, U1293, Université Claude Bernard Lyon 1, Ecole Normale Supérieure de Lyon, 46 Allée d'Italie, 69364, Lyon, France.

4. Centre Blaise Pascal de Simulation et de Modélisation Numérique, Ecole Normale Supérieure de Lyon, 46 Allée d'Italie, 69364, Lyon, France.

*** Corresponding Authors**

Siewert J. Marrink: s.j.marrink@rug.nl, Paulo C.T. Souza: paulo.telles_de_souza@ens-lyon.fr,

Syma Khalid: syma.khalid@bioch.ox.ac.uk.

Supplementary Material

Contents

Figure S1: Distribution of bonded terms for 3 replicas of 1 us each using the GLYCAM-06j forcefield after mapping it to a coarse-grained trajectory……………………………………………………………….……………4
Figure S2: Mean values of bonded terms for 3 replicas of 1 us each using the GLYCAM-06j forcefield after mapping to a coarse-grained trajetory…….…………………………………………………………...…...5
Table S1: Comparative analysis of all bonded terms using CHARMM36 or GLYCAM-06j force field after mapping to a coarse-grained trajectory in a subset of disaccharides...6
Table S2: Overlap in distributions for bonded terms using CHARMM36 , GLYCAM-06j and Martini 3…. .7
Figure S3: Distribution of bonded terms for trehalose Glc(α1→ 1)Glcα: 1GA_0GA)....................................8
Figure S4: SASA distribution for trehalose Glc(α1→ 1)Glcα: 1GA_0GA)...8
Table S3: Overlap in distributions for trehalose (Glc(α1→ 1)Glcα: 1GA_0GA) and Glc(α1→1)Glcβ (1GB_0GA) for Martini 3 and Martini 3 original...9
Figure S5: Distribution of bonded terms for trehalose(Glc(α1→ 1)Glcα) and for Glc(α1→ 1)Glcβ….........10
Figure S6: Comparative analysis of all bonded term distributions using CHARMM36 GLYCAM-06j or Martini 3 in a subset of disaccharides...11
Figure S7: Comparative analysis of all bonded term distributions using CHARMM36 GLYCAM-06j or Martini 3 in a subset of disaccharides...12
Figure S8: Comparative coarse-grained and all-atom distributions of bonded terms and SASA shown for 4 different disaccharides (part 1) ……………………………………………………………….…..............13
Figure S9: Comparative coarse-grained and all-atom distributions of bonded terms and SASA shown for 4 different disaccharides (part 2) ……………………………………………………….................................14
Figure S10: Comparative coarse-grained and all-atom distributions of bonded terms and SASA shown for 4 different disaccharides (part 3) ……………………………………………………………….…..............15
Figure S11: Comparative coarse-grained and all-atom distributions of bonded terms and SASA shown for 4 different disaccharides (part 4) ……………………………………………………………….…..............16
Figure S12: Comparative coarse-grained and all-atom distributions of bonded terms and SASA shown for 4 different disaccharides (part 5) ……………………………………………………………….…..............17
Figure S13: Comparative coarse-grained and all-atom distributions of bonded terms and SASA shown for 4 different disaccharides (part 6) ……………………………………………………………….…..............18
Figure S14: Comparative coarse-grained and all-atom distributions of bonded terms and SASA shown for 4 different disaccharides (part 7) ……………………………………………………………….…..............19
Figure S15: Comparative coarse-grained and all-atom distributions of bonded terms and SASA shown for 4 different disaccharides (part 8) ……………………………………………………………….…..............20
Figure S16: Comparative coarse-grained and all-atom distributions of bonded terms for all the different disaccharides (part 1)..21
Figure S17: Comparative coarse-grained and all-atom distributions of bonded terms for all the different disaccharides (part 2)..22
Figure S18: Comparative coarse-grained and all-atom distributions of bonded terms for all the different disaccharides (part 3)..23
Figure S19: Comparative coarse-grained and all-atom distributions of bonded terms for all the different disaccharides (part 4)..24
Figure S20: Aggregation data for the 1GA_0GA disaccharide using the Martini2 coarse-grained forcefield (CG)...25
Figure S21: Aggregation data for the 1GA_0GA disaccharide using the Martini 3 coarse-grained forcefield (CG)...26
Figure S22: Aggregation data for the 1GB_0GB disaccharide using the Martini 3 coarse-grained forcefield (CG)...27
Figure S23: Aggregation data for the 2MA_0MA disaccharide using the Martini 3 coarse-grained forcefield (CG)...28
Figure S24: Aggregation data for the 4MA_0MA disaccharide using the Martini 3 coarse-grained forcefield (CG)...29
Figure S25: Aggregation data for the 6GB_0GB disaccharide using the Martini 3 coarse-grained forcefield (CG)...30

Figure S26: Aggregation data for the 6MB_0MB disaccharide using the Martini 3 coarse-grained forcefield (CG)........31
Figure S27: Aggregation data for the 6MA_0MB disaccharide using the Martini 3 coarse-grained forcefield (CG)........32
Figure S28: Aggregation data for the 3GB_0GB disaccharide using the Martini 3 coarse-grained forcefield (CG)........33
Figure S29: Additional aggregation data from extension of the Glycam-06j trajectories to 2 microseconds........34

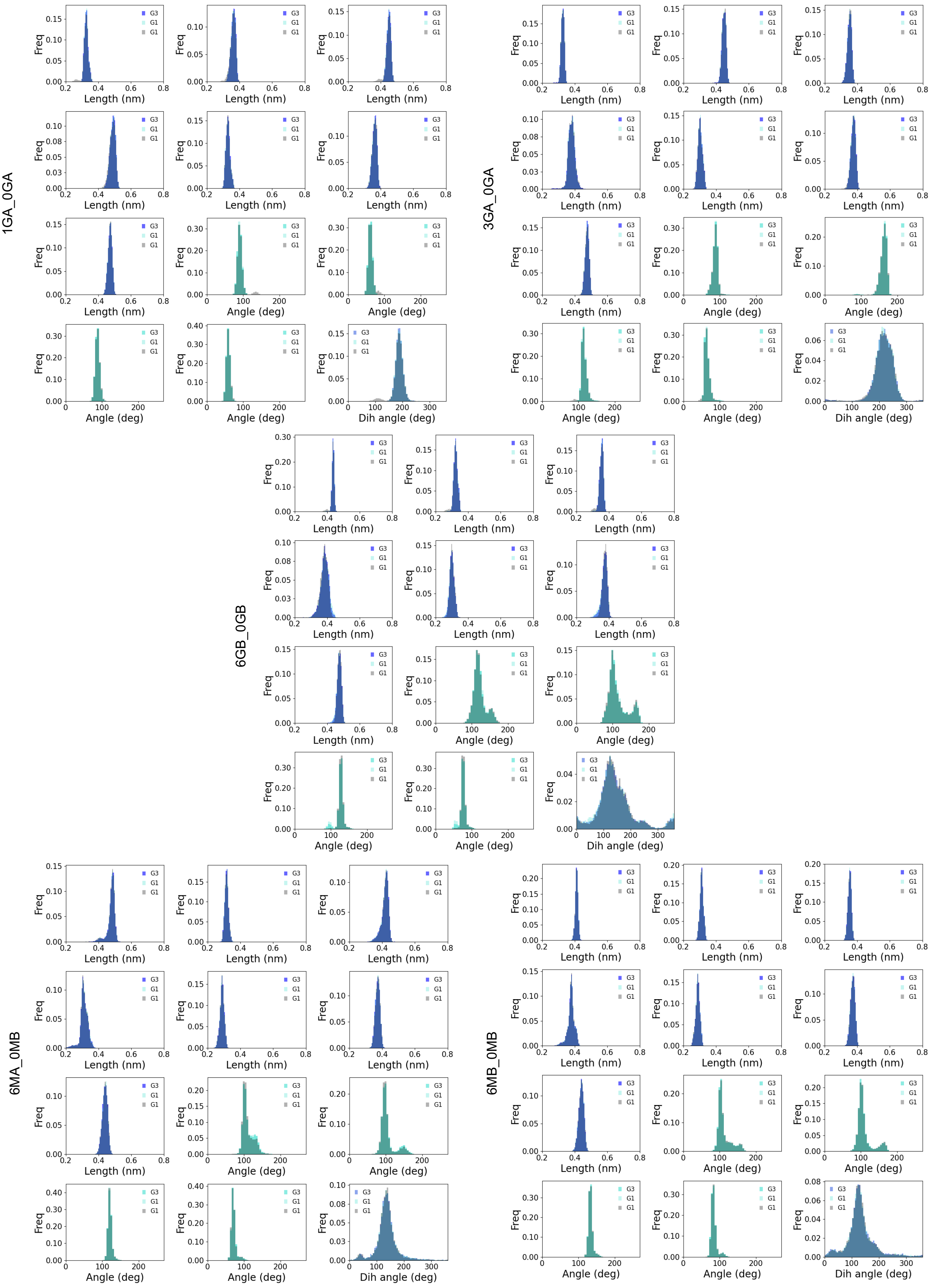

Figure S1: Distribution of bonded terms for 3 replicas of 1 us each using the GLYCAM-06j forcefield after mapping it to a coarse-grained trajectory. Distributions from each replica are shown in different colours. All the 7 present bonds in each disaccharide are plotted for each replica (G1-G3, first 7 plots). The harmonic bond between both monomers is presented (middle top row of each panel). All the four defined angles, and the unique dihedral (defining the glycosidic bond) are plotted too.

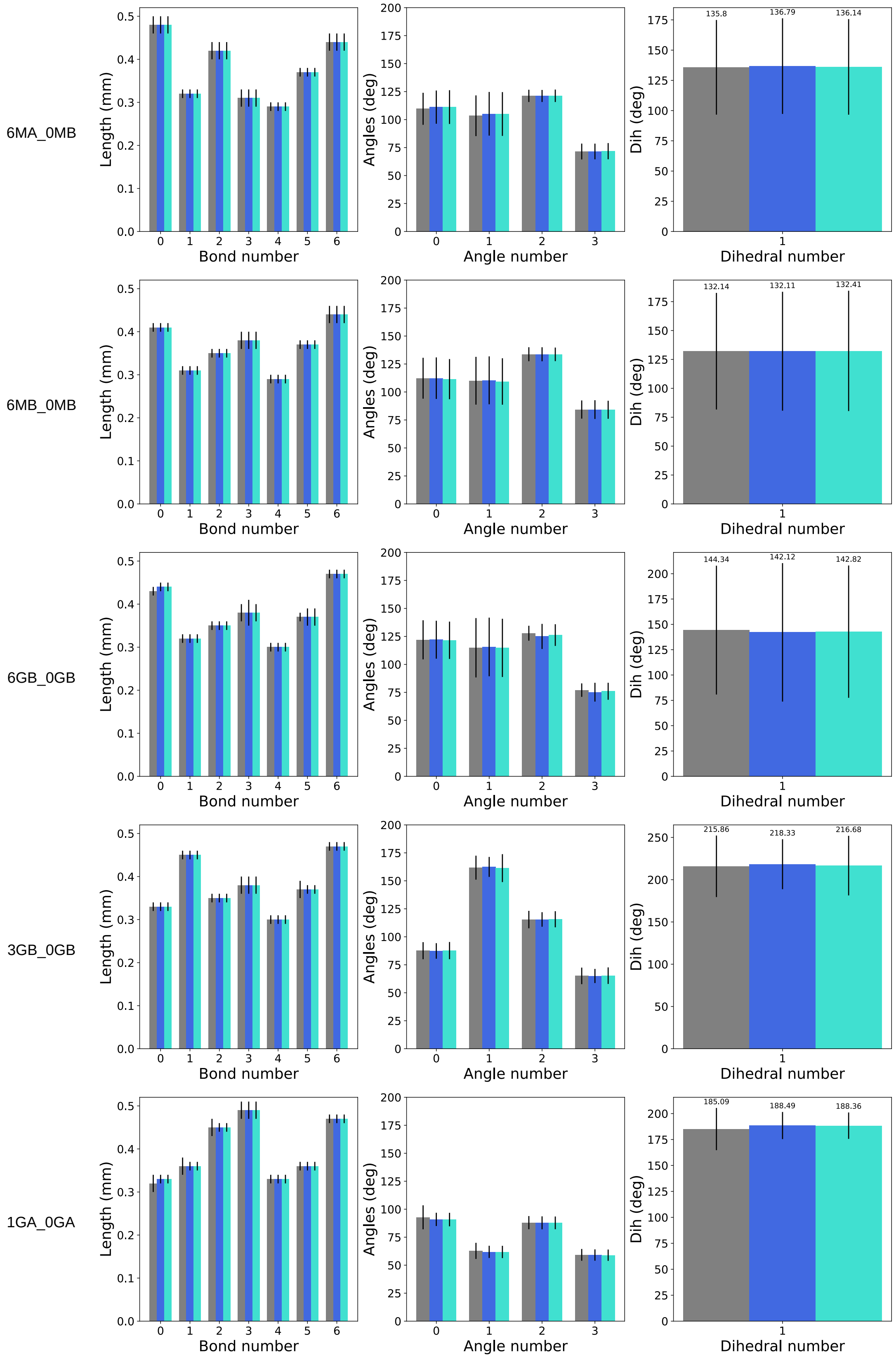

Figure S2: Mean values of bonded terms for 3 replicas of 1 us each using the GLYCAM-06j forcefield after mapping to a coarse-grained trajectory. Mean values for every bonded term of each replica are shown in different coloured bars with their associated standard deviation. All the 7 present bonds in each disaccharide are plotted for each replica (bond number 0-6). For reference, the harmonic bond between both monomers has bond number 3. All the four defined angles, and the unique dihedral (defining the glycosidic bond) are plotted too. The mean dihedral values are reported in the barplot.

α 1→1	Charmm36m avg	±	std	GLYCAM-06j avg	±	std	Diff	Diff_error	% deviation
BOND0 (nm)	0.32	±	0.01	**0.32**	±	**0.01**	*0.00*	*0.01*	*0.00*
BOND1 (nm)	0.35	±	0.01	**0.36**	±	**0.02**	*0.01*	*0.02*	*2.78*
BOND2 (nm)	0.43	±	0.01	**0.41**	±	**0.01**	*-0.02*	*0.01*	*-4.88*
BOND3 (nm)	0.45	±	0.02	**0.37**	±	**0.02**	*-0.08*	*0.03*	*-21.62*
BOND4 (nm)	0.32	±	0.01	**0.33**	±	**0.01**	*0.01*	*0.01*	*3.03*
BOND5 (nm)	0.35	±	0.01	**0.36**	±	**0.01**	*0.01*	*0.01*	*2.78*
BOND6 (nm)	0.45	±	0.01	**0.47**	±	**0.01**	*0.02*	*0.01*	*4.26*
ANG0 (deg)	91.43	±	5.68	**122.7**	±	**8.86**	*31.27*	*10.52*	*25.48*
ANG1 (deg)	62.12	±	5.16	**165.63**	±	**8.98**	*103.51*	*10.36*	*62.49*
ANG2 (deg)	83.83	±	5.46	**106.91**	±	**6.32**	*23.08*	*8.35*	*21.59*
ANG3 (deg)	57.26	±	4.64	**72.71**	±	**6.81**	*15.45*	*8.24*	*21.25*
DIH (deg)	183.73	±	13.07	**245.48**	±	**108.28**	*61.75*	*109.07*	*25.15*

α 1→2	Charmm36m avg	±	std	GLYCAM-06j avg	±	std	Diff	Diff_error	% deviation
BOND0 (nm)	0.33	±	0.01	**0.32**	±	**0.01**	*-0.01*	*0.01*	*-3.13*
BOND1 (nm)	0.35	±	0.01	**0.36**	±	**0.02**	*0.01*	*0.02*	*2.78*
BOND2 (nm)	0.4	±	0.01	**0.41**	±	**0.01**	*0.01*	*0.01*	*2.44*
BOND3 (nm)	0.34	±	0.02	**0.37**	±	**0.02**	*0.03*	*0.03*	*8.11*
BOND4 (nm)	0.32	±	0.01	**0.33**	±	**0.01**	*0.01*	*0.01*	*3.03*
BOND5 (nm)	0.35	±	0.01	**0.36**	±	**0.01**	*0.01*	*0.01*	*2.78*
BOND6 (nm)	0.45	±	0.01	**0.47**	±	**0.01**	*0.02*	*0.01*	*4.26*
ANG0 (deg)	123.44	±	9.28	**122.7**	±	**8.86**	*-0.74*	*12.83*	*-0.60*
ANG1 (deg)	167.24	±	8.21	**165.63**	±	**8.98**	*-1.61*	*12.17*	*-0.97*
ANG2 (deg)	108.04	±	7.06	**106.91**	±	**6.32**	*-1.13*	*9.48*	*-1.06*
ANG3 (deg)	74.19	±	8.02	**72.71**	±	**6.81**	*-1.48*	*10.52*	*-2.04*
DIH (deg)	279.96	±	83.57	**245.48**	±	**108.28**	*-34.48*	*136.78*	*-14.05*

α1→3	Charmm36m avg	±	std	GLYCAM-06j avg	±	std	Diff	Diff_error	% deviation
BOND0 (nm)	0.34	±	0.01	**0.33**	±	**0.01**	*-0.01*	*0.01*	*-3.03*
BOND1 (nm)	0.43	±	0.01	**0.43**	±	**0.01**	*0.00*	*0.01*	*0.00*
BOND2 (nm)	0.32	±	0.01	**0.33**	±	**0.01**	*0.01*	*0.01*	*3.03*
BOND3 (nm)	0.34	±	0.03	**0.36**	±	**0.03**	*0.02*	*0.04*	*5.56*
BOND4 (nm)	0.32	±	0.01	**0.33**	±	**0.01**	*0.01*	*0.01*	*3.03*
BOND5 (nm)	0.35	±	0.01	**0.36**	±	**0.01**	*0.01*	*0.01*	*2.78*
BOND6 (nm)	0.45	±	0.01	**0.47**	±	**0.01**	*0.02*	*0.01*	*4.26*
ANG0 (deg)	110.02	±	11.29	**108.2**	±	**9.71**	*-1.82*	*14.89*	*-1.68*
ANG1 (deg)	160.51	±	8.52	**160.74**	±	**9.76**	*0.23*	*12.96*	*0.14*
ANG2 (deg)	112.77	±	10.14	**112.85**	±	**9.19**	*0.08*	*13.68*	*0.07*
ANG3 (deg)	82.89	±	10.48	**82.41**	±	**8.01**	*-0.48*	*13.19*	*-0.58*
DIH (deg)	224.89	±	144.66	**208.42**	±	**141.99**	*-16.47*	*202.70*	*-7.90*

α 1→4	Charmm36m avg	±	std	GLYCAM-06j avg	±	std	Diff	Diff_error	% deviation
BOND0 (nm)	0.31	±	0.01	**0.3**	±	**0.01**	*-0.01*	*0.01*	*-3.33*
BOND1 (nm)	0.43	±	0.01	**0.43**	±	**0.02**	*0.00*	*0.02*	*0.00*
BOND2 (nm)	0.36	±	0.01	**0.36**	±	**0.02**	*0.00*	*0.02*	*0.00*
BOND3 (nm)	0.32	±	0.03	**0.35**	±	**0.03**	*0.03*	*0.04*	*8.57*
BOND4 (nm)	0.32	±	0.01	**0.32**	±	**0.02**	*0.00*	*0.02*	*0.00*
BOND5 (nm)	0.35	±	0.01	**0.36**	±	**0.02**	*0.01*	*0.02*	*2.78*
BOND6 (nm)	0.45	±	0.01	**0.47**	±	**0.02**	*0.02*	*0.02*	*4.26*
ANG0 (deg)	159.64	±	11.44	**154.88**	±	**19.16**	*-4.76*	*22.32*	*-3.07*
ANG1 (deg)	105.57	±	12.61	**104.76**	±	**9.18**	*-0.81*	*15.60*	*-0.77*
ANG2 (deg)	112.02	±	9.94	**115.62**	±	**8.16**	*3.60*	*12.86*	*3.11*
ANG3 (deg)	80.31	±	11.85	**78.5**	±	**8.68**	*-1.81*	*14.69*	*-2.31*
DIH (deg)	189.22	±	38.17	**207.66**	±	**35.35**	*18.44*	*52.02*	*8.88*

α 1→6	Charmm36m avg	±	std	GLYCAM-06j avg	±	std	Diff	Diff_error	% deviation
BOND0 (nm)	0.45	±	0.01	**0.42**	±	**0.01**	*-0.03*	*0.01*	*-7.14*
BOND1 (nm)	0.33	±	0.01	**0.33**	±	**0.01**	*0.00*	*0.01*	*0.00*
BOND2 (nm)	0.35	±	0.01	**0.34**	±	**0.01**	*-0.01*	*0.01*	*-2.94*
BOND3 (nm)	0.38	±	0.02	**0.41**	±	**0.02**	*0.03*	*0.03*	*7.32*
BOND4 (nm)	0.32	±	0.01	**0.33**	±	**0.01**	*0.01*	*0.01*	*3.03*
BOND5 (nm)	0.35	±	0.01	**0.36**	±	**0.01**	*0.01*	*0.01*	*2.78*
BOND6 (nm)	0.45	±	0.01	**0.47**	±	**0.01**	*0.02*	*0.01*	*4.26*
ANG0 (deg)	115.19	±	13.87	**120.52**	±	**16.69**	*5.33*	*21.70*	*4.42*
ANG1 (deg)	127.64	±	22.8	**115.66**	±	**15.71**	*-11.98*	*27.69*	*-10.36*
ANG2 (deg)	95.22	±	5.54	**97.87**	±	**6.03**	*2.65*	*8.19*	*2.71*
ANG3 (deg)	61.13	±	5.07	**66.46**	±	**5.53**	*5.33*	*7.50*	*8.02*
DIH (deg)	232.2	±	37.09	**231.77**	±	**40.89**	*-0.43*	*55.21*	*-0.19*

β 1→1	Charmm36m avg	±	std	GLYCAM-06j avg	±	std	Diff	Diff_error	% deviation
BOND0 (nm)	0.33	±	0.01	**0.3**	±	**0.01**	*-0.03*	*0.01*	*-10.00*
BOND1 (nm)	0.35	±	0.01	**0.37**	±	**0.01**	*0.02*	*0.01*	*5.41*
BOND2 (nm)	0.44	±	0.01	**0.45**	±	**0.01**	*0.01*	*0.01*	*2.22*
BOND3 (nm)	0.4	±	0.02	**0.44**	±	**0.02**	*0.04*	*0.03*	*9.09*
BOND4 (nm)	0.31	±	0.01	**0.3**	±	**0.01**	*-0.01*	*0.01*	*-3.33*
BOND5 (nm)	0.35	±	0.01	**0.38**	±	**0.01**	*0.03*	*0.01*	*7.89*
BOND6 (nm)	0.45	±	0.01	**0.47**	±	**0.01**	*0.02*	*0.01*	*4.26*
ANG0 (deg)	134.56	±	6.41	**133.64**	±	**7.05**	*-0.92*	*9.53*	*-0.69*
ANG1 (deg)	83.58	±	6.07	**81.55**	±	**6.08**	*-2.03*	*8.59*	*-2.49*
ANG2 (deg)	128.14	±	5.86	**127.27**	±	**5.95**	*-0.87*	*8.35*	*-0.68*
ANG3 (deg)	77.85	±	5.46	**76.83**	±	**5.3**	*-1.02*	*7.61*	*-1.33*
DIH (deg)	260.11	±	18.89	**243.01**	±	**18.77**	*-17.10*	*26.63*	*-7.04*

β 1→2	Charmm36m avg	±	std	GLYCAM-06j avg	±	std	Diff	Diff_error	% deviation
BOND0 (nm)	0.35	±	0.01	**0.32**	±	**0.01**	*-0.03*	*0.01*	*-9.37*
BOND1 (nm)	0.35	±	0.01	**0.37**	±	**0.01**	*0.02*	*0.01*	*5.41*
BOND2 (nm)	0.41	±	0.01	**0.42**	±	**0.01**	*0.01*	*0.01*	*2.38*
BOND3 (nm)	0.37	±	0.02	**0.39**	±	**0.02**	*0.02*	*0.03*	*5.13*
BOND4 (nm)	0.31	±	0.01	**0.3**	±	**0.01**	*-0.01*	*0.01*	*-3.33*
BOND5 (nm)	0.35	±	0.01	**0.37**	±	**0.01**	*0.02*	*0.01*	*5.41*
BOND6 (nm)	0.45	±	0.01	**0.47**	±	**0.01**	*0.02*	*0.01*	*4.26*
ANG0 (deg)	91.18	±	6.99	**86.87**	±	**8.84**	*-4.31*	*11.27*	*-4.96*
ANG1 (deg)	144.03	±	6.67	**142.13**	±	**8.23**	*-1.90*	*10.59*	*-1.34*
ANG2 (deg)	115.72	±	5.65	**123.19**	±	**6.65**	*7.47*	*8.73*	*6.06*
ANG3 (deg)	65.1	±	5.64	**72.42**	±	**6.36**	*7.32*	*8.50*	*10.11*
DIH (deg)	228.98	±	36.63	**222.03**	±	**49.12**	*-6.95*	*61.27*	*-3.13*

β 1→3	Charmm36m avg	±	std	GLYCAM-06j avg	±	std	Diff	Diff_error	% deviation
BOND0 (nm)	0.35	±	0.01	**0.33**	±	**0.01**	*-0.02*	*0.01*	*-6.06*
BOND1 (nm)	0.39	±	0.04	**0.45**	±	**0.01**	*0.06*	*0.04*	*13.33*
BOND2 (nm)	0.12	±	0.03	**0.35**	±	**0.01**	*0.23*	*0.03*	*65.71*
BOND3 (nm)	0.39	±	0.02	**0.38**	±	**0.02**	*-0.01*	*0.03*	*-2.63*
BOND4 (nm)	0.31	±	0.01	**0.3**	±	**0.01**	*-0.01*	*0.01*	*-3.33*
BOND5 (nm)	0.35	±	0.01	**0.37**	±	**0.02**	*0.02*	*0.02*	*5.41*
BOND6 (nm)	0.45	±	0.01	**0.47**	±	**0.01**	*0.02*	*0.01*	*4.26*
ANG0 (deg)	83.7	±	7.11	**87.6**	±	**7.62**	*3.90*	*10.42*	*4.45*
ANG1 (deg)	141.14	±	13.75	**161.78**	±	**10.7**	*20.64*	*17.42*	*12.76*
ANG2 (deg)	115.07	±	6.99	**115.41**	±	**7.79**	*0.34*	*10.47*	*0.29*
ANG3 (deg)	64.88	±	7.3	**65.09**	±	**7.39**	*0.21*	*10.39*	*0.32*
DIH (deg)	226.04	±	42.26	**215.86**	±	**36.39**	*-10.18*	*55.77*	*-4.72*

β 1→4	Charmm36m avg	±	std	GLYCAM-06j avg	±	std	Diff	Diff_error	% deviation
BOND0 (nm)	0.33	±	0.01	**0.31**	±	**0.01**	*-0.02*	*0.01*	*-6.45*
BOND1 (nm)	0.43	±	0.01	**0.45**	±	**0.01**	*0.02*	*0.01*	*4.44*
BOND2 (nm)	0.35	±	0.01	**0.38**	±	**0.01**	*0.03*	*0.01*	*7.89*
BOND3 (nm)	0.36	±	0.02	**0.4**	±	**0.02**	*0.04*	*0.03*	*10.00*
BOND4 (nm)	0.31	±	0.01	**0.3**	±	**0.01**	*-0.01*	*0.01*	*-3.33*
BOND5 (nm)	0.35	±	0.01	**0.37**	±	**0.01**	*0.02*	*0.01*	*5.41*
BOND6 (nm)	0.45	±	0.01	**0.47**	±	**0.01**	*0.02*	*0.01*	*4.26*
ANG0 (deg)	150.69	±	5.51	**143.86**	±	**5.79**	*-6.83*	*7.99*	*-4.75*
ANG1 (deg)	74.15	±	6.21	**65**	±	**6.13**	*-9.15*	*8.73*	*-14.08*
ANG2 (deg)	112.43	±	5.04	**113.45**	±	**6.71**	*1.02*	*8.39*	*0.90*
ANG3 (deg)	61.78	±	5.26	**63.14**	±	**6.64**	*1.36*	*8.47*	*2.15*
DIH (deg)	202.29	±	40.63	**205.39**	±	**28.85**	*3.10*	*49.83*	*1.51*

β 1→6	Charmm36m	±	std	GLYCAM-06j avg	±	std	Diff	Diff_error	% deviation
BOND0 (nm)	0.46	±	0.01	**0.43**	±	**0.01**	*-0.03*	*0.01*	*-6.98*
BOND1 (nm)	0.35	±	0.01	**0.32**	±	**0.01**	*-0.03*	*0.01*	*-9.37*
BOND2 (nm)	0.35	±	0.01	**0.35**	±	**0.01**	*0.00*	*0.01*	*0.00*
BOND3 (nm)	0.36	±	0.02	**0.38**	±	**0.02**	*0.02*	*0.03*	*5.26*
BOND4 (nm)	0.31	±	0.01	**0.3**	±	**0.01**	*-0.01*	*0.01*	*-3.33*
BOND5 (nm)	0.35	±	0.01	**0.37**	±	**0.01**	*0.02*	*0.01*	*5.41*
BOND6 (nm)	0.45	±	0.01	**0.47**	±	**0.01**	*0.02*	*0.01*	*4.26*
ANG0 (deg)	112.15	±	9.05	**121.97**	±	**17.47**	*9.82*	*19.67*	*8.05*
ANG1 (deg)	129.63	±	33.11	**114.85**	±	**26.51**	*-14.78*	*42.42*	*-12.87*
ANG2 (deg)	120.98	±	4.14	**127.85**	±	**6.68**	*6.87*	*7.86*	*5.37*
ANG3 (deg)	70.22	±	4.42	**77.02**	±	**6.01**	*6.80*	*7.46*	*8.83*
DIH (deg)	146.89	±	43.54	**144.34**	±	**63.58**	*-2.55*	*77.06*	*-1.77*

Difference: GLYCAM-06j - Charmm36m
% deviation: (Diff/GLYCAM-06j)*100

Table S1: Comparative analysis of all bonded terms using CHARMM36 or GLYCAM-06j force field after mapping to a coarse-grained trajectory in a subset of disaccharides.

Average values and their associated standard deviations were computed for all the bonded terms for each1 us-long all-atom simulation. The values coming from simulations used for parameterization are highlighted in bold. The difference between the GLYCAM-06j average values with its CHARMM36 counterpart are reported in the column ”Diff”. The cases where the average values from both forcefield almost agree (i.e same values within the error), are highlighted in yellow. Wherever there is a disagreement they are highlighted in orange. The non-highlighted lines correspond to the averages that match within the error.

	α 1→1						α 1→2						α1→3					
	All-atoms			CG / GLYCAM-06j			All-atoms			CG / GLYCAM-06j			All-atoms			CG / GLYCAM-06j		
	full	ref		full	ref		full	ref		full	ref		full	ref		full	ref	
BOND0_overlap	0.62	0.765	TRUE	0.351	0.52	FALSE	0.508	0.673	TRUE	0.508	0.674	TRUE	0.508	0.673	TRUE	0.508	0.674	TRUE
BOND1_overlap	0.609	0.757	TRUE	0.253	0.404	FALSE	0.617	0.763	TRUE	0.281	0.439	FALSE	0.617	0.763	TRUE	0.281	0.439	FALSE
BOND2_overlap	0.303	0.465	FALSE	0.296	0.457	FALSE	0.667	0.8	TRUE	0.326	0.492	FALSE	0.667	0.8	TRUE	0.326	0.492	FALSE
BOND3_overlap	0.261	0.414	**FALSE**	0.721	0.838	**TRUE**	0.42	0.591	**FALSE**	0.739	0.85	**TRUE**	0.42	0.591	**FALSE**	0.739	0.85	**TRUE**
BOND4_overlap	0.603	0.753	TRUE	0.353	0.522	FALSE	0.638	0.779	TRUE	0.35	0.518	FALSE	0.638	0.779	TRUE	0.35	0.518	FALSE
BOND5_overlap	0.603	0.752	TRUE	0.281	0.438	FALSE	0.582	0.736	TRUE	0.283	0.441	FALSE	0.582	0.736	TRUE	0.283	0.441	FALSE
BOND6_overlap	0.322	0.487	FALSE	0.335	0.502	FALSE	0.314	0.478	FALSE	0.327	0.493	FALSE	0.314	0.478	FALSE	0.327	0.493	FALSE
ANG0_overlap	0.875	0.934	TRUE	0.457	0.627	TRUE	0.721	0.838	TRUE	0.735	0.847	TRUE	0.721	0.838	TRUE	0.735	0.847	TRUE
ANG1_overlap	0.885	0.939	TRUE	0.769	0.869	TRUE	0.82	0.901	TRUE	0.673	0.805	TRUE	0.82	0.901	TRUE	0.673	0.805	TRUE
ANG2_overlap	0.562	0.72	TRUE	0.926	0.962	TRUE	0.821	0.902	TRUE	0.83	0.907	TRUE	0.821	0.902	TRUE	0.83	0.907	TRUE
ANG3_overlap	0.762	0.865	TRUE	0.787	0.881	TRUE	0.787	0.881	TRUE	0.744	0.853	TRUE	0.787	0.881	TRUE	0.744	0.853	TRUE
DIH_overlap	0.716	0.834	**TRUE**	0.62	0.765	**TRUE**	0.652	0.789	**TRUE**	0.492	0.659	**TRUE**	0.652	0.789	**TRUE**	0.492	0.659	**TRUE**
			9			6			10			7			10			7

	α 1→4						α 1→6					
	All-atom			CG / GLYCAM-06j			All-atoms			CG / GLYCAM-06j		
	full	ref		full	ref		full	ref		full	ref	
BOND0_overlap	0.498	0.665	TRUE	0.435	0.607	TRUE	0.036	0.069	FALSE	0.707	0.828	TRUE
BOND1_overlap	0.606	0.755	TRUE	0.273	0.429	FALSE	0.676	0.807	TRUE	0.421	0.592	FALSE
BOND2_overlap	0.651	0.789	TRUE	0.288	0.448	FALSE	0.601	0.751	TRUE	0.511	0.676	TRUE
BOND3_overlap	0.383	0.554	**FALSE**	0.603	0.752	**TRUE**	0.312	0.476	**FALSE**	0.707	0.829	**TRUE**
BOND4_overlap	0.607	0.756	TRUE	0.315	0.479	FALSE	0.656	0.792	TRUE	0.356	0.526	FALSE
BOND5_overlap	0.671	0.803	TRUE	0.26	0.413	FALSE	0.613	0.76	TRUE	0.288	0.447	FALSE
BOND6_overlap	0.36	0.529	FALSE	0.278	0.435	FALSE	0.305	0.468	FALSE	0.33	0.496	FALSE
ANG0_overlap	0.79	0.883	TRUE	0.468	0.638	TRUE	0.571	0.727	TRUE	0.621	0.766	TRUE
ANG1_overlap	0.634	0.776	TRUE	0.792	0.884	TRUE	0.641	0.781	TRUE	0.615	0.761	TRUE
ANG2_overlap	0.737	0.849	TRUE	0.863	0.926	TRUE	0.689	0.816	TRUE	0.843	0.915	TRUE
ANG3_overlap	0.518	0.683	TRUE	0.785	0.879	TRUE	0.471	0.64	TRUE	0.758	0.862	TRUE
DIH_overlap	0.648	0.787	**TRUE**	0.697	0.82	**TRUE**	0.76	0.863	**TRUE**	0.604	0.753	**TRUE**
			10			7			9			8

	β 1→1						β 1→2						β1→3					
	All-atoms			CG / GLYCAM-06j			All-atoms			CG / GLYCAM-06j			All-atoms			CG / GLYCAM-06j		
	full	ref		full	ref		full	ref		full	ref		full	ref		full	ref	
BOND0_overlap	0.185	0.312	FALSE	0.357	0.527	FALSE	0.112	0.202	FALSE	0.461	0.631	TRUE	0.122	0.218	FALSE	0.423	0.594	FALSE
BOND1_overlap	0.296	0.457	FALSE	0.289	0.448	FALSE	0.32	0.485	FALSE	0.299	0.46	FALSE	0.117	0.209	FALSE	0.349	0.518	FALSE
BOND2_overlap	0.321	0.486	FALSE	0.357	0.526	FALSE	0.587	0.739	TRUE	0.337	0.504	FALSE	0	0	FALSE	0.345	0.513	FALSE
BOND3_overlap	0.191	0.321	**FALSE**	0.76	0.864	**TRUE**	0.375	0.545	**FALSE**	0.616	0.762	**TRUE**	0.757	0.862	**TRUE**	0.747	0.855	**TRUE**
BOND4_overlap	0.776	0.874	TRUE	0.36	0.53	FALSE	0.797	0.887	TRUE	0.32	0.485	FALSE	0.802	0.89	TRUE	0.317	0.482	FALSE
BOND5_overlap	0.278	0.435	FALSE	0.291	0.451	FALSE	0.299	0.46	FALSE	0.29	0.449	FALSE	0.305	0.468	FALSE	0.292	0.451	FALSE
BOND6_overlap	0.142	0.248	FALSE	0.344	0.512	FALSE	0.144	0.252	FALSE	0.363	0.532	FALSE	0.139	0.244	FALSE	0.359	0.529	FALSE
ANG0_overlap	0.91	0.953	TRUE	0.755	0.861	TRUE	0.542	0.703	TRUE	0.838	0.912	TRUE	0.561	0.719	TRUE	0.729	0.843	TRUE
ANG1_overlap	0.77	0.87	TRUE	0.956	0.978	TRUE	0.777	0.874	TRUE	0.72	0.837	TRUE	0.204	0.339	FALSE	0.664	0.798	TRUE
ANG2_overlap	0.894	0.944	TRUE	0.685	0.813	TRUE	0.374	0.544	FALSE	0.805	0.892	TRUE	0.861	0.925	TRUE	0.775	0.873	TRUE
ANG3_overlap	0.859	0.924	TRUE	0.937	0.967	TRUE	0.353	0.522	FALSE	0.788	0.881	TRUE	0.837	0.911	TRUE	0.693	0.819	TRUE
DIH_overlap	0.464	0.634	**TRUE**	0.832	0.908	**TRUE**	0.775	0.873	**TRUE**	0.484	0.652	**TRUE**	0.566	0.723	**TRUE**	0.744	0.853	**TRUE**
			6			6			5			7			6			6

	β1→4						αβ1→6					
	All-atoms			CG / GLYCAM-06j			All-atom			CG / GLYCAM-06j		
	full	ref		full	ref		full	ref		full	ref	
BOND0_overlap	0.216	0.356	FALSE	0.382	0.553	FALSE	0.111	0.199	FALSE	0.568	0.724	TRUE
BOND1_overlap	0.422	0.593	FALSE	0.342	0.509	FALSE	0.173	0.295	FALSE	0.388	0.559	FALSE
BOND2_overlap	0.203	0.338	FALSE	0.375	0.545	FALSE	0.618	0.764	TRUE	0.355	0.524	FALSE
BOND3_overlap	0.19	0.32	**FALSE**	0.863	0.927	**TRUE**	0.358	0.527	**FALSE**	0.64	0.781	**TRUE**
BOND4_overlap	0.815	0.898	TRUE	0.334	0.501	FALSE	0.783	0.878	TRUE	0.341	0.509	FALSE
BOND5_overlap	0.304	0.467	FALSE	0.29	0.449	FALSE	0.293	0.454	FALSE	0.297	0.458	FALSE
BOND6_overlap	0.146	0.255	FALSE	0.348	0.516	FALSE	0.139	0.244	FALSE	0.348	0.517	FALSE
ANG0_overlap	0.36	0.53	FALSE	0.875	0.933	TRUE	0.557	0.715	TRUE	0.634	0.776	TRUE
ANG1_overlap	0.29	0.45	FALSE	0.586	0.739	TRUE	0.45	0.621	TRUE	0.587	0.74	TRUE
ANG2_overlap	0.841	0.914	TRUE	0.734	0.847	TRUE	0.302	0.464	FALSE	0.835	0.91	TRUE
ANG3_overlap	0.822	0.902	TRUE	0.783	0.878	TRUE	0.309	0.472	FALSE	0.704	0.826	TRUE
DIH_overlap	0.91	0.953	**TRUE**	0.734	0.847	**TRUE**	0.691	0.818	**TRUE**	0.368	0.538	**FALSE**
			4			6			5			6

Table S2: Overlap in distributions for all bonded terms using CHARMM36 , GLYCAM-06j and Martini 3. Two types of overlaps are reported: complete overlap between the 2 distributions (full), or overlapped ratio of GLYCAM-06j (ref). These were computed between CHARMM36 and GLYCAM-06j (Labeled All-atoms) or between Martini 3 and GLYCAM-06j (CG/GLYCAM-06j). The existence of an overlap was computed if the reference overlap is larger than 0.6 (TRUE) and the total incidences reported below.

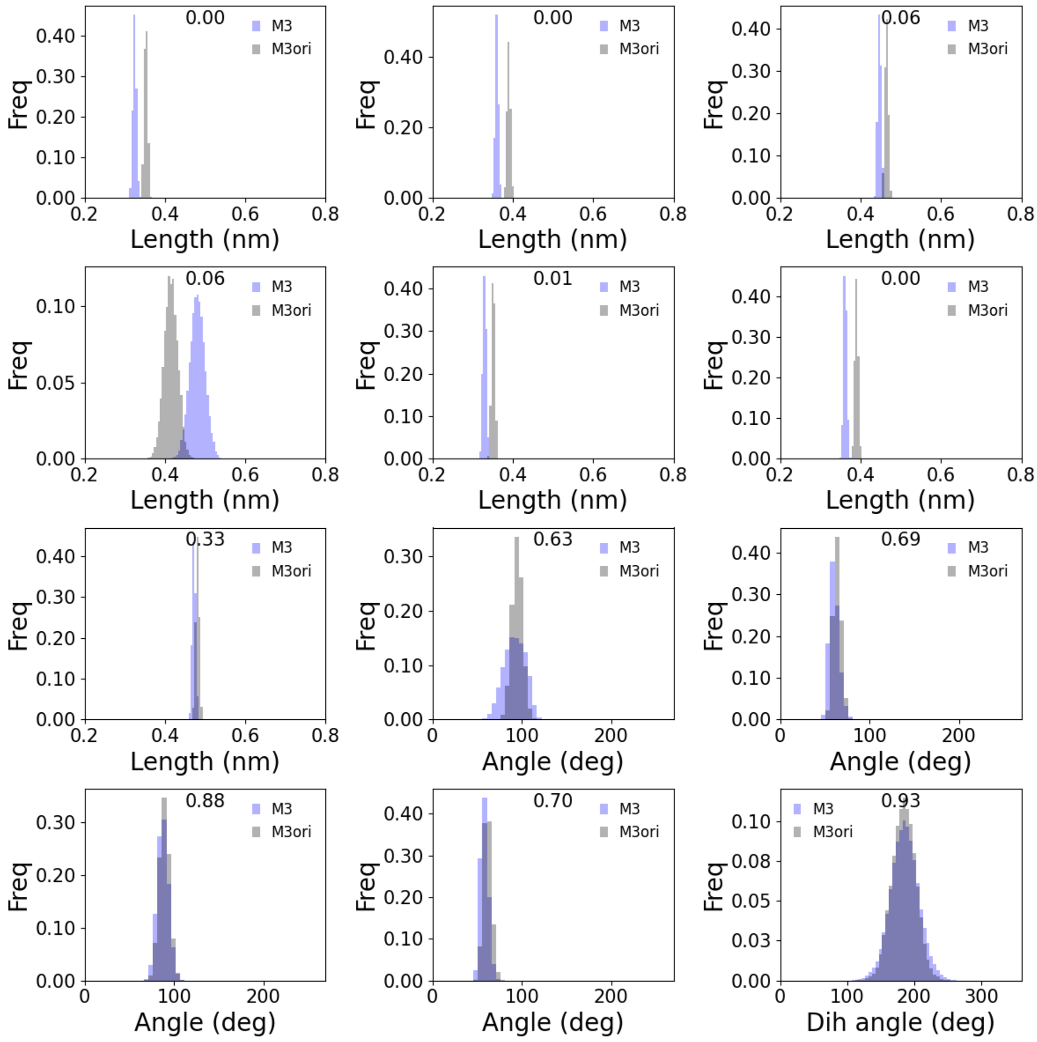

Figure S3: Distribution of bonded terms for trehalose Glc(α1→ 1)Glcα: 1GA_0GA)
Distributions obtained from simulations using the Martini 3 original trehalose paramaeters (grey, M3ori) and the automated parameters (light blue, M3). The value on the top centre of each panel corresponds to the reference overlap value.

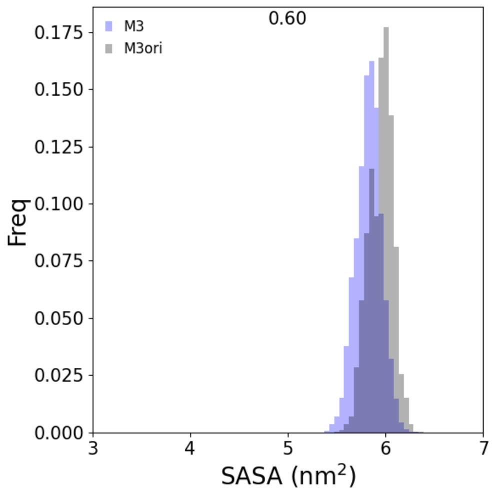

Figure S4: SASA distribution for trehalose Glc(α1→ 1)Glcα: 1GA_0GA)
Distribution obtained from simulations using the Martini 3 original trehalose parameters (grey, M3ori) and the automated parameters (light blue, M3). The value on the top centre of each panel corresponds to the reference overlap value.

	1GA_0GA					
	Martini 3 original		Martini 3		Overlaps	
	AVG	STD	AVG	STD	full	ref
BOND0 (nm)	0.35	0	0.32	0	0	0
BOND1 (nm)	0.39	0	0.36	0	0	0
BOND2 (nm)	0.47	0	0.45	0	0.03	0.059
BOND3 (nm)	*0.41*	*0.02*	*0.48*	*0.02*	*0.032*	*0.062*
BOND4 (nm)	0.35	0	0.33	0	0.004	0.008
BOND5 (nm)	0.39	0	0.36	0	0	0
BOND6 (nm)	0.48	0	0.47	0	0.195	0.327
ANG0 (deg)	*95.06*	*5.82*	*92.13*	*11.87*	*0.461*	***0.631***
ANG1(deg)	*63.98*	*4.36*	*60.75*	*5.37*	*0.531*	***0.694***
ANG2 (deg)	*89.34*	*5.7*	*87.72*	*6.26*	*0.79*	***0.883***
ANG3 (deg)	*61.83*	*4.34*	*58.31*	*4.27*	*0.543*	***0.704***
DIH (deg)	*184.63*	*17.85*	*185.85*	*20.99*	*0.866*	***0.928***
SASA (nm2)	5.83	0.13	5.95	0.12	0.43	**0.6**

	1GB_0GA					
	Martini 3 original		Martini 3		Overlaps	
	AVG	STD	AVG	STD	full	ref
BOND0 (nm)	0.35	0	0.3	0	0	0
BOND1 (nm)	0.39	0	0.37	0	0.026	0.05
BOND2 (nm)	0.47	0	0.45	0	0.08	0.149
BOND3 (nm)	0.41	0.02	0.42	0.02	0.66	0.795
BOND4 (nm)	0.35	0	0.33	0	0.004	0.007
BOND5 (nm)	0.39	0	0.36	0	0	0
BOND6 (nm)	0.48	0	0.47	0	0.185	0.312
ANG0 (deg)	95.06	5.82	150.69	6.62	0	0
ANG1(deg)	63.98	4.36	102.53	6.19	0	0
ANG2 (deg)	89.34	5.7	105.01	8.34	0.149	0.26
ANG3 (deg)	61.83	4.34	76.41	6.27	0.118	0.211
DIH (deg)	184.63	17.85	129.49	129.2	0.019	0.036
SASA (nm2)	4.47	0.18	5.95	0.12	0	0

Table S3: Overlap in distributions for trehalose (Glc(α1→ 1)Glcα: 1GA_0GA) and Glc(α1→ 1)Glcβ (1GB_0GA) for Martini 3 and Martini 3 original.

Average and standard deviation values are reported for each of the analysed properties. Two types of overlaps are reported: complete overlap between the 2 distributions (full), or overlapped ratio of Martini 3 original (ref). The existence of an overlap was computed if the reference overlap is larger than 0.6 (TRUE) and the total incidences reported below.

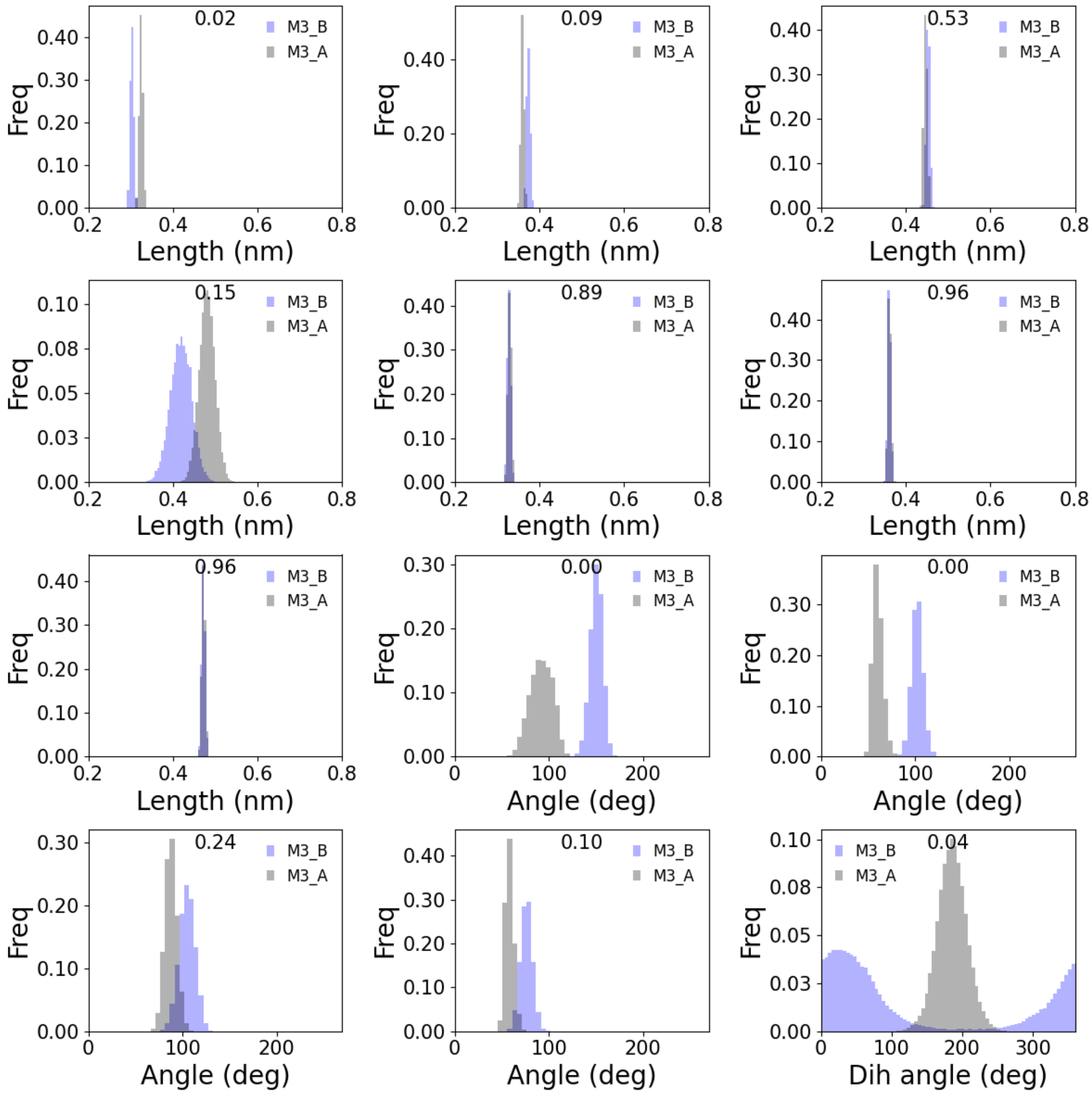

Figure S5: Distribution of bonded terms for trehalose(Glc(α1→ 1)Glcα) and for Glc(α1→ 1)Glcβ
Distributions obtained from simulations of (grey, M3_A) and with the anomeric -OH group oriented in beta conformation (light blue, M3_B). The value on the top centre of each panel corresponds to the reference overlap value respect to the alpha anomer.

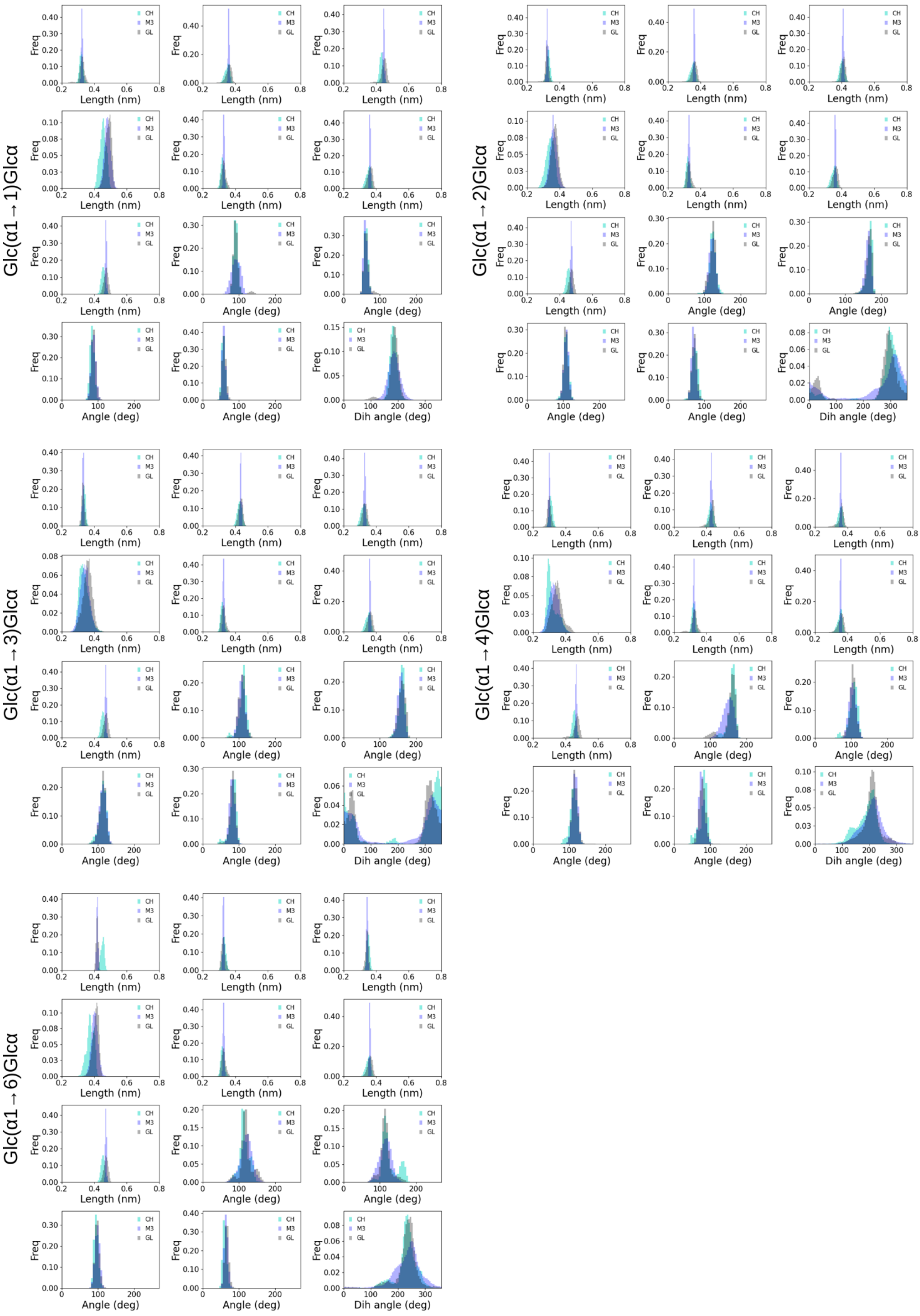

Figure S6: Comparative analysis of all bonded term distributions using CHARMM36 GLYCAM-06j or Martini 3 in a subset of disaccharides. The data from these plots is the data used to create Table S1 for the dimers between glucoses that have an alpha glycosidic bond. Labels are as follow: CH: CHARMM36, M3: Martini 3, GL: GLYCAM-06j

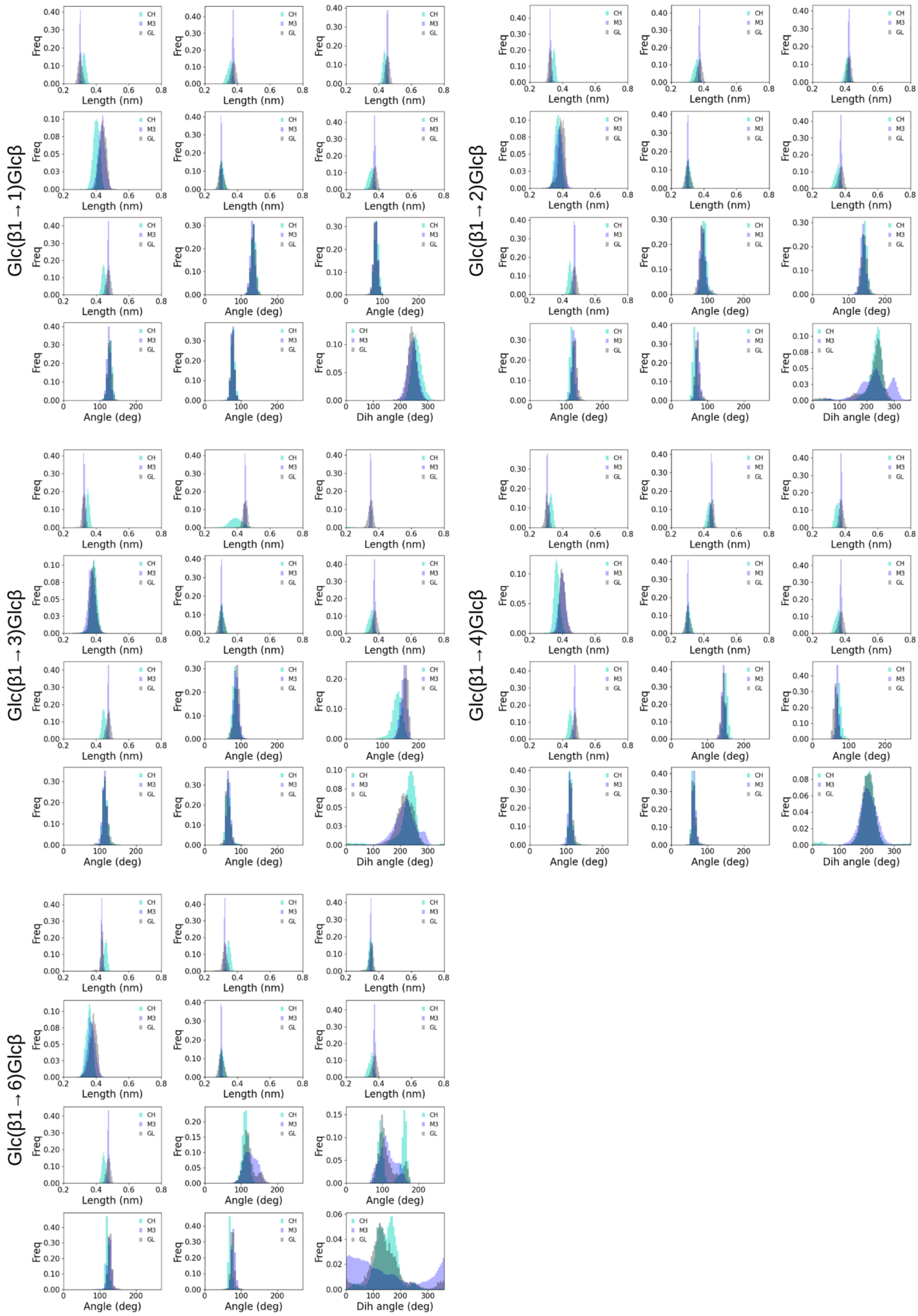

Figure S7: Comparative analysis of all bonded term distributions using CHARMM36 GLYCAM-06j or Martini 3 in a subset of disaccharides. The data from these plots is the data used to create Table S1 for the dimers between glucoses that have a beta glycosidic bond. Labels are as follow: CH: CHARMM36, M3: Martini 3, GL: GLYCAM-06j

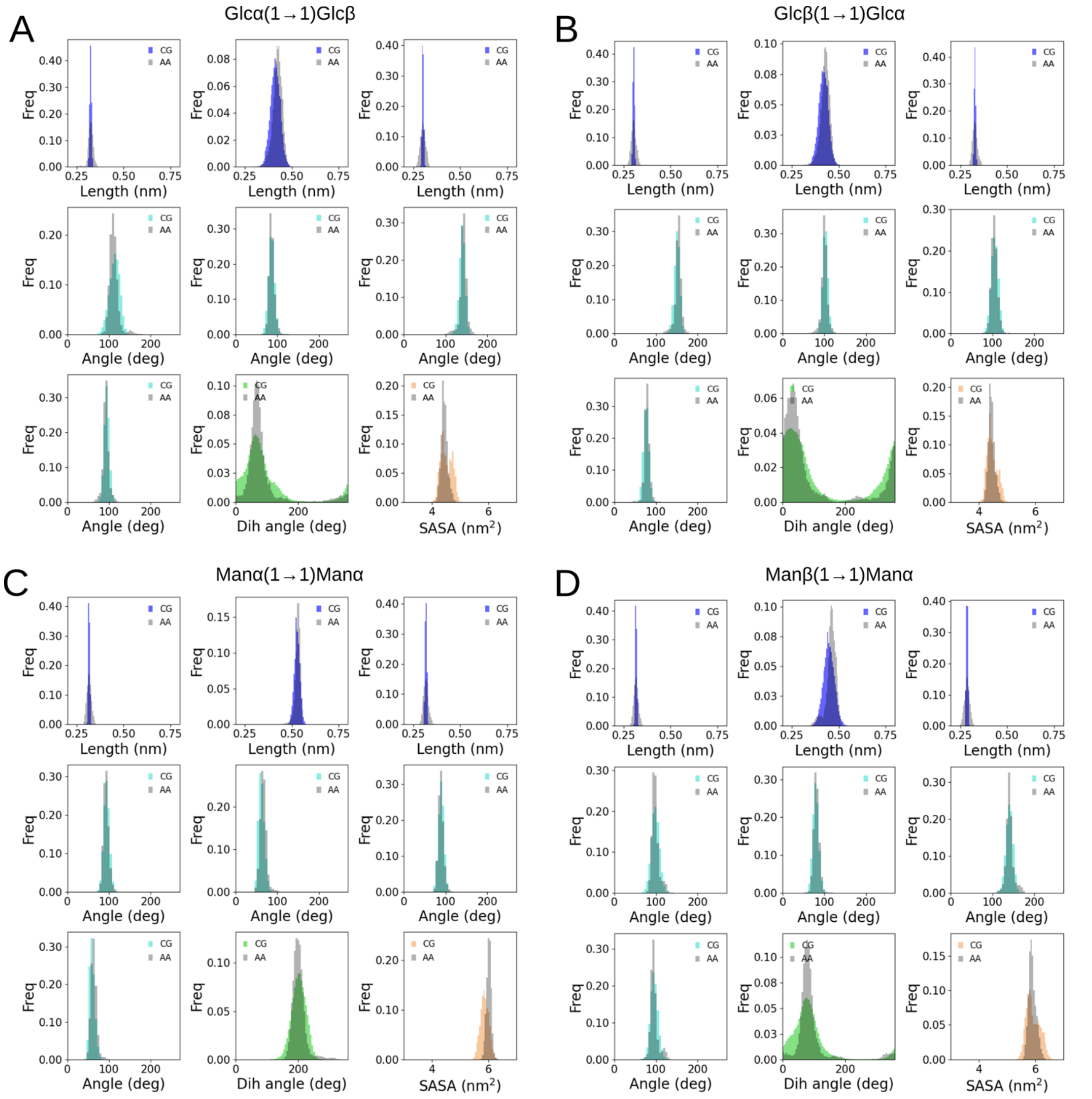

Figure S8: Comparative coarse-grained and all-atom distributions of bonded terms and SASA shown for 4 different disaccharides(A-D). Distributions from CG simulations are shown in colours and the ones coming from all-atom (GLYCAM-06j) simulations are shown in grey. For the sake of space, for each monosaccharide modelled as a ring, only 1 constraint (out of 3) between beads that define a ring is shown (first row of each panel- left and right). The elastic bond between both monomers is presented (middle top row of each panel). All the four defined angles (middle and bottom rows cyan: CG, grey AA), the unique dihedral (green: CG, grey: AA) and SASA (orange: CG, grey: AA) are plotted too.

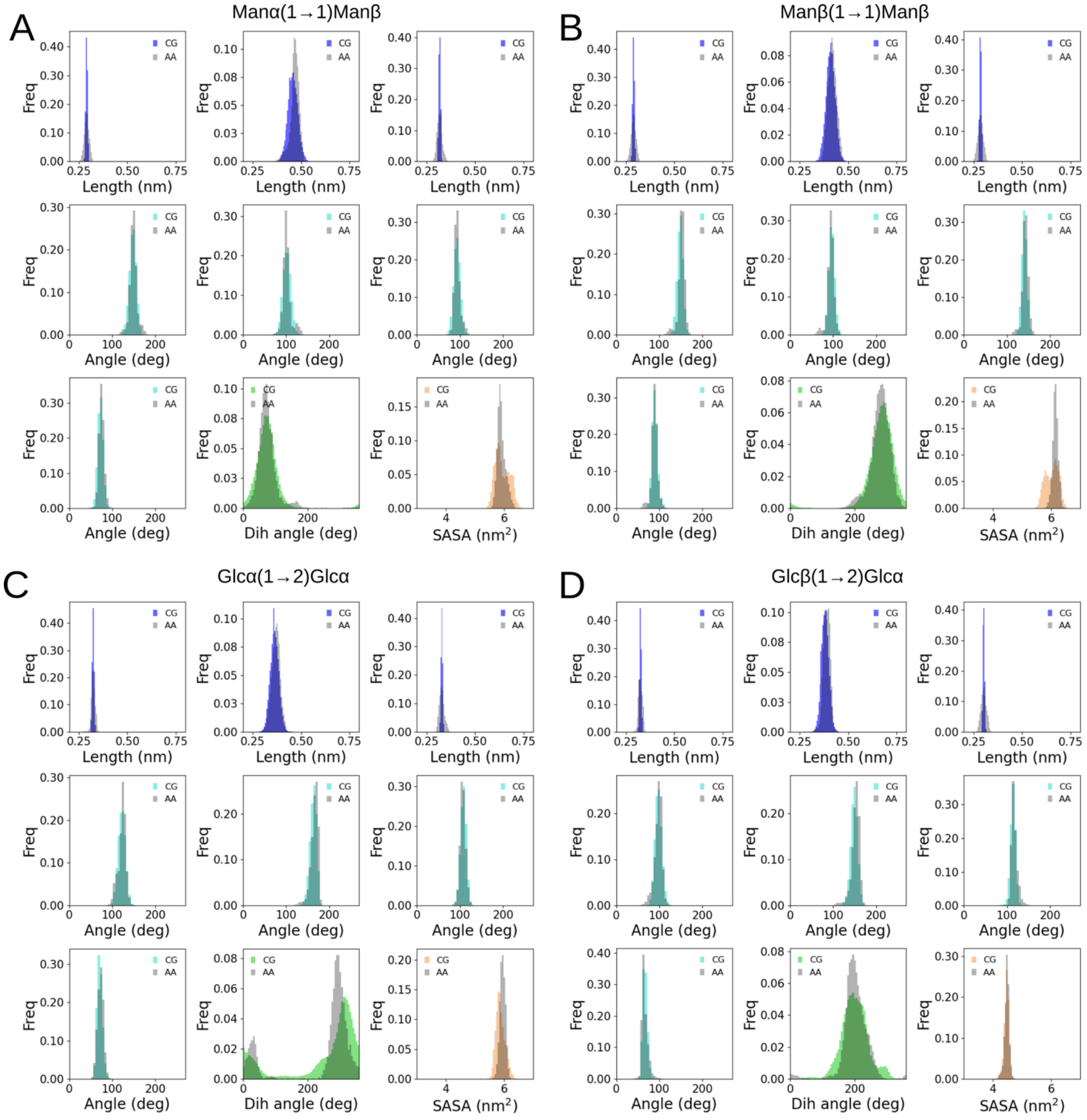

Figure S9: Comparative coarse-grained and all-atom distributions of bonded terms and SASA shown for 4 different disaccharides(A-D). Distributions from CG simulations are shown in colours and the ones coming from all-atom (GLYCAM-06j) simulations are shown in grey. For the sake of space, for each monosaccharide modelled as a ring, only 1 constraint (out of 3) between beads that define a ring is shown (first row of each panel- left and right). The elastic bond between both monomers is presented (middle top row of each panel). All the four defined angles (middle and bottom rows cyan: CG, grey AA), the unique dihedral (green: CG, grey: AA) and SASA (orange: CG, grey: AA) are plotted too.

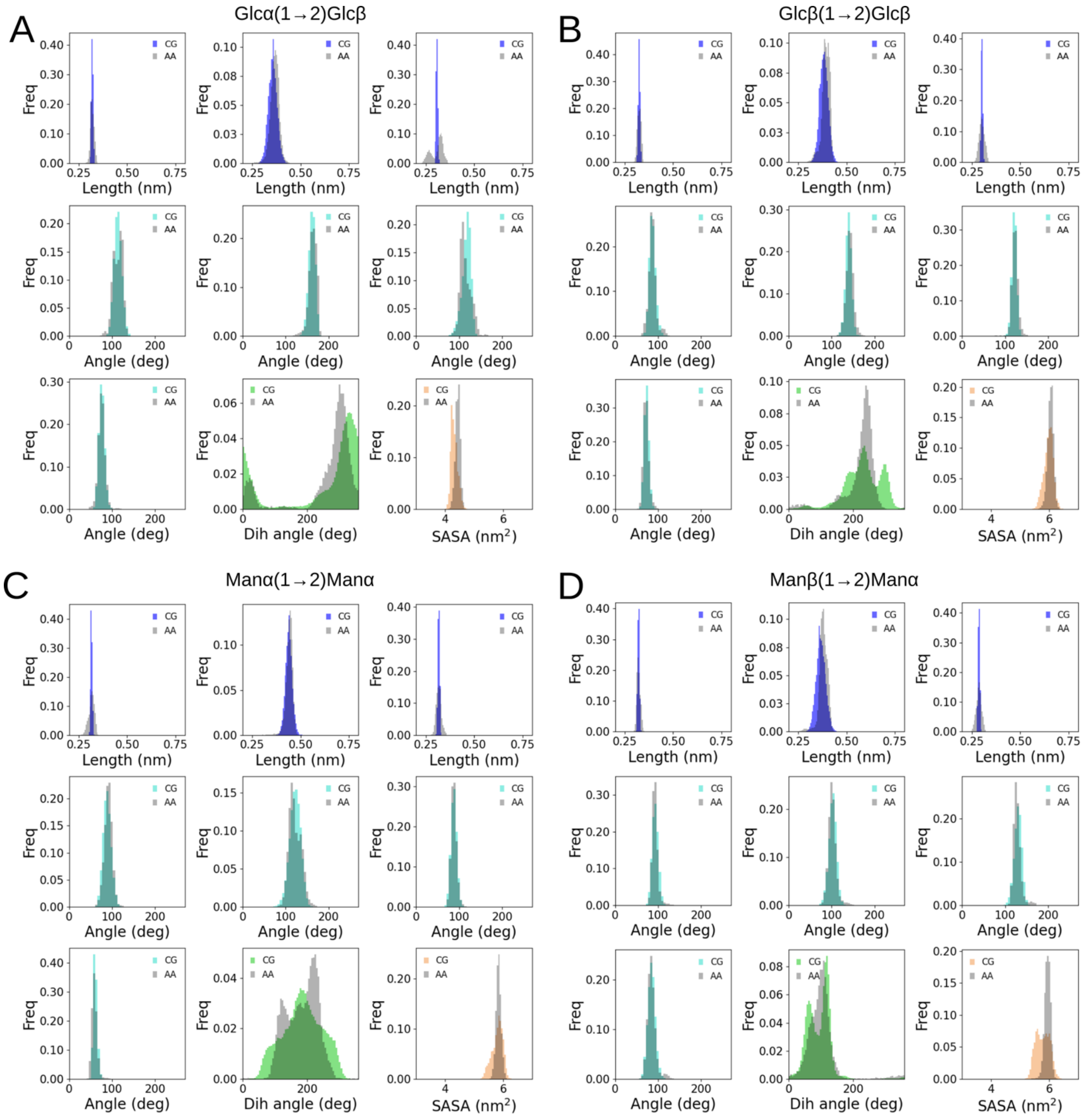

Figure S10: Comparative coarse-grained and all-atom distributions of bonded terms and SASA shown for 4 different disaccharides(A-D). Distributions from CG simulations are shown in colours and the ones coming from all-atom (GLYCAM-06j) simulations are shown in grey. For the sake of space, for each monosaccharide modelled as a ring, only 1 constraint (out of 3) between beads that define a ring is shown (first row of each panel- left and right). The elastic bond between both monomers is presented (middle top row of each panel). All the four defined angles (middle and bottom rows cyan: CG, grey AA), the unique dihedral (green: CG, grey: AA) and SASA (orange: CG, grey: AA) are plotted too.

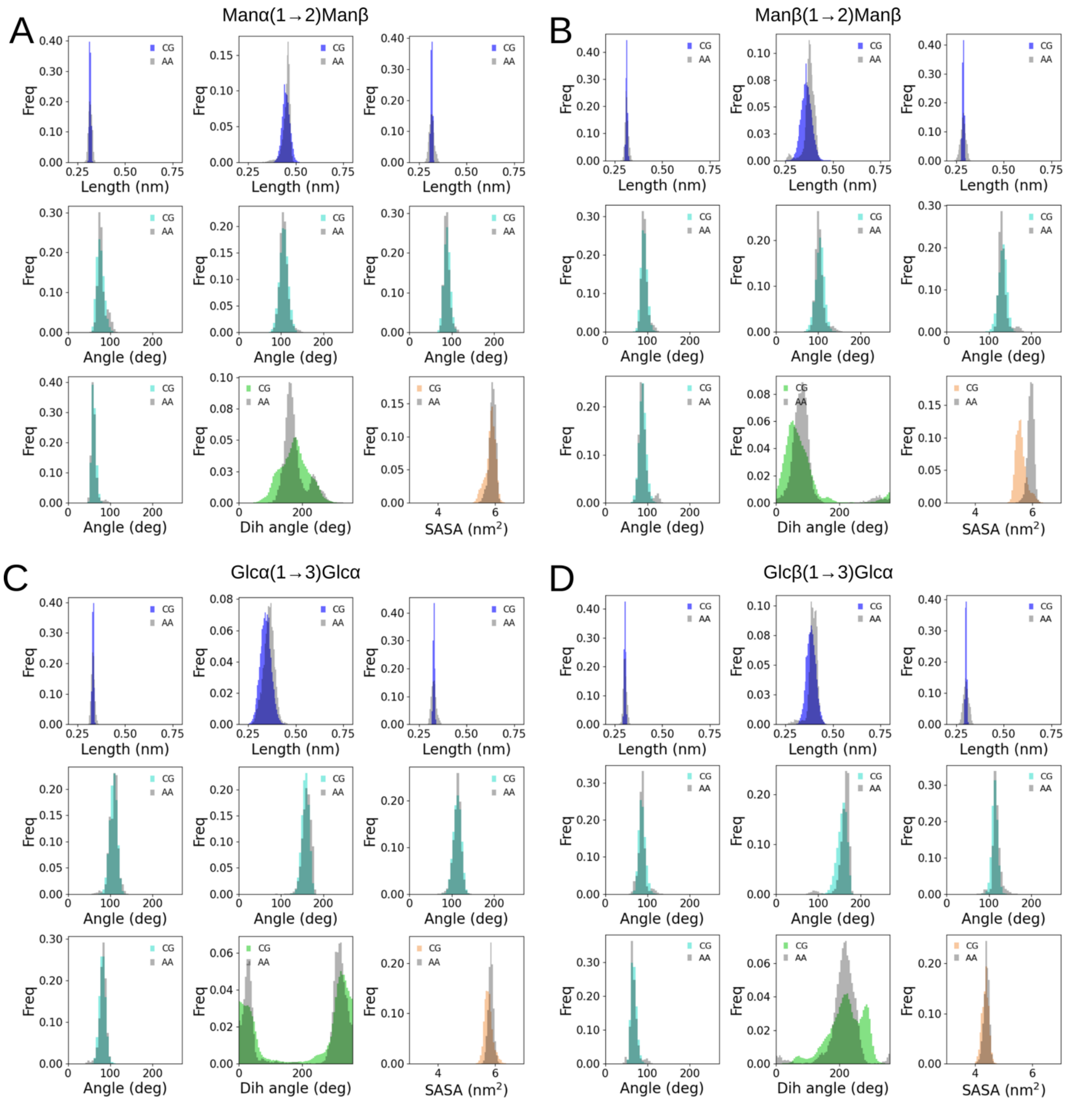

Figure S11: Comparative coarse-grained and all-atom distributions of bonded terms and SASA shown for 4 different disaccharides(A-D). Distributions from CG simulations are shown in colours and the ones coming from all-atom (GLYCAM-06j) simulations are shown in grey. For the sake of space, for each monosaccharide modelled as a ring, only 1 constraint (out of 3) between beads that define a ring is shown (first row of each panel- left and right). The elastic bond between both monomers is presented (middle top row of each panel). All the four defined angles (middle and bottom rows cyan: CG, grey AA), the unique dihedral (green: CG, grey: AA) and SASA (orange: CG, grey: AA) are plotted too.

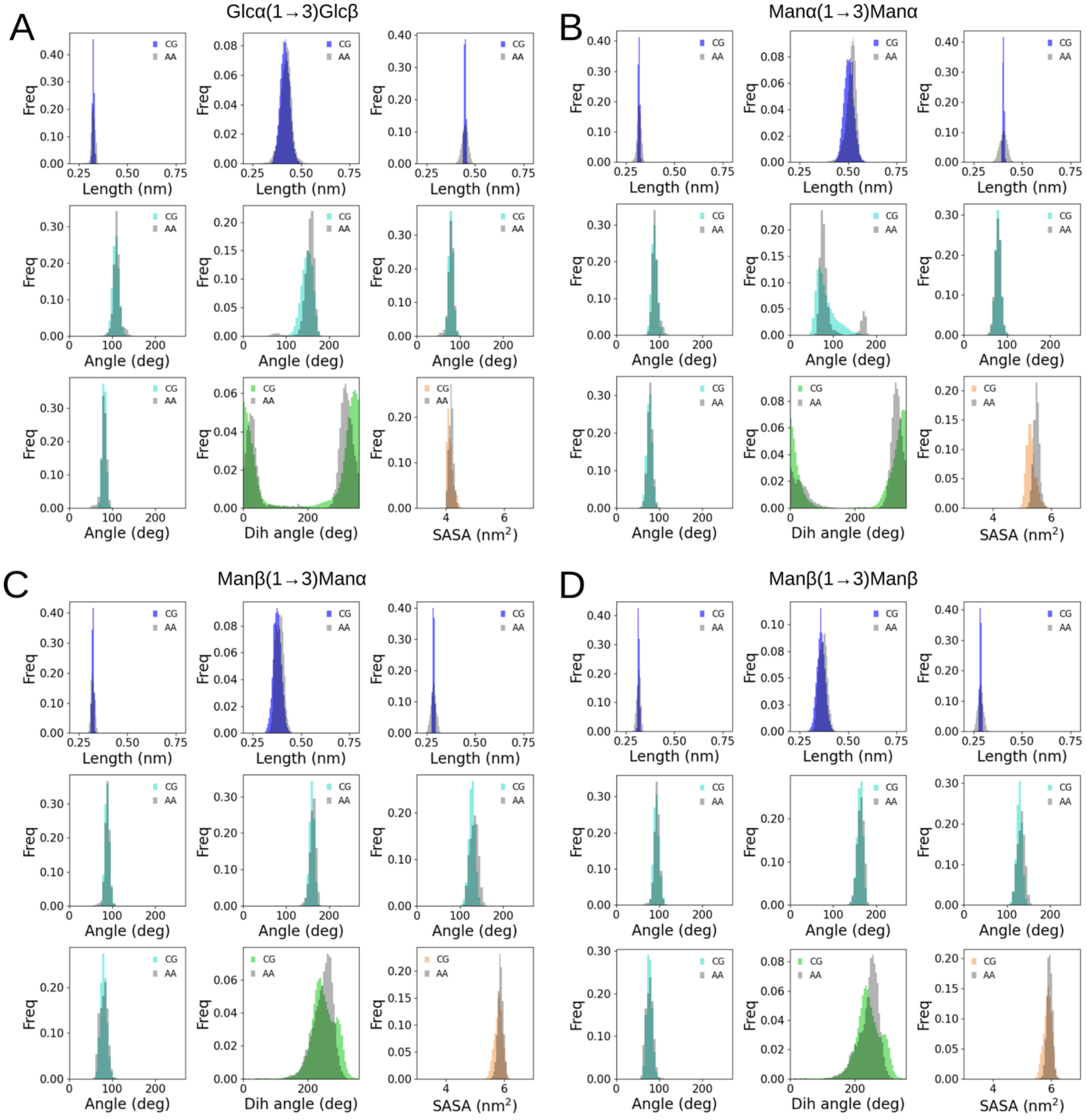

Figure S12: Comparative coarse-grained and all-atom distributions of bonded terms and SASA shown for 4 different disaccharides(A-D). Distributions from CG simulations are shown in colours and the ones coming from all-atom (GLYCAM-06j) simulations are shown in grey. For the sake of space, for each monosaccharide modelled as a ring, only 1 constraint (out of 3) between beads that define a ring is shown (first row of each panel- left and right). The elastic bond between both monomers is presented (middle top row of each panel). All the four defined angles (middle and bottom rows cyan: CG, grey AA), the unique dihedral (green: CG, grey: AA) and SASA (orange: CG, grey: AA) are plotted too.

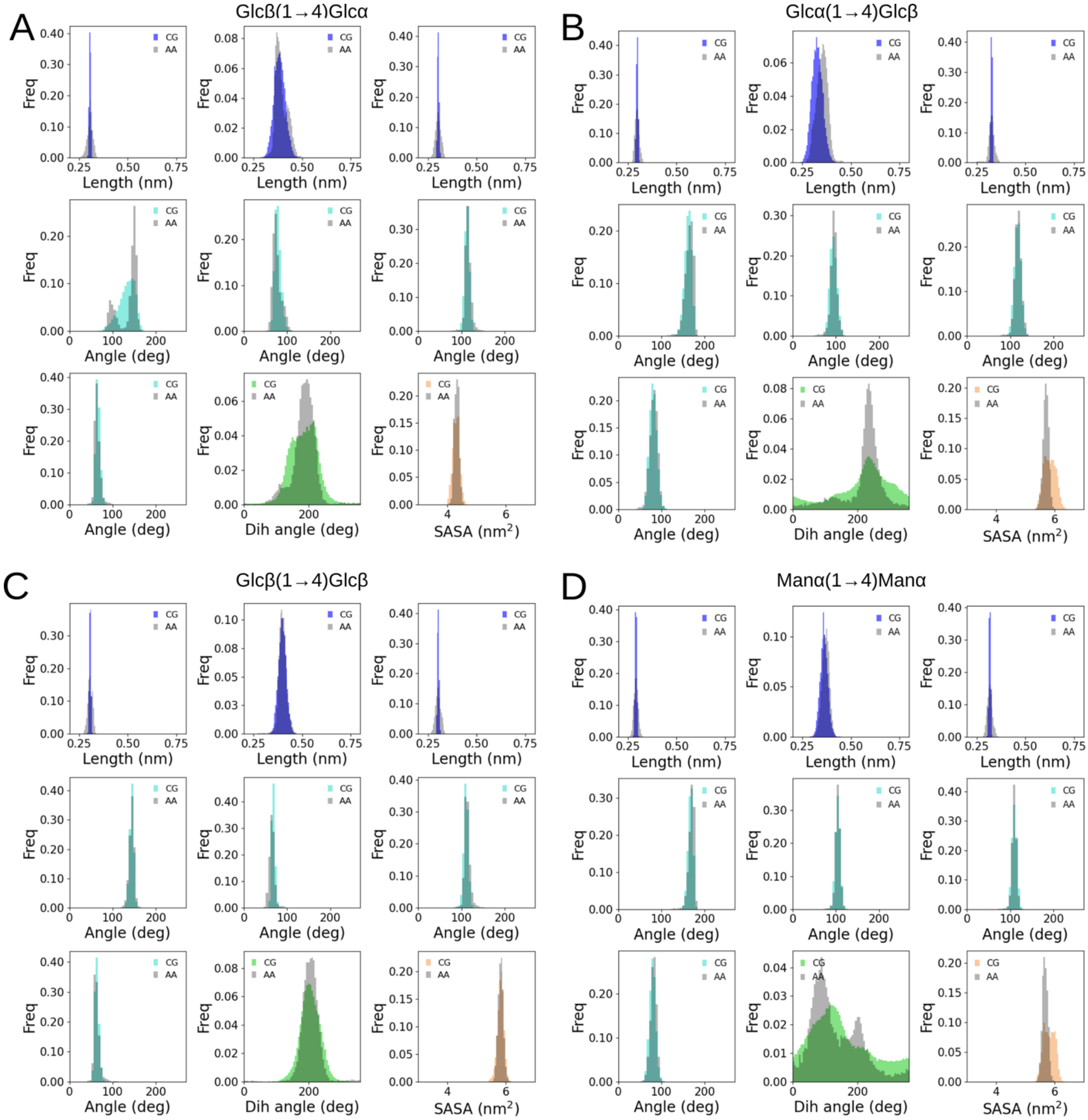

Figure S13: Comparative coarse-grained and all-atom distributions of bonded terms and SASA shown for 4 different disaccharides(A-D). Distributions from CG simulations are shown in colours and the ones coming from all-atom (GLYCAM-06j) simulations are shown in grey. For the sake of space, for each monosaccharide modelled as a ring, only 1 constraint (out of 3) between beads that define a ring is shown (first row of each panel- left and right). The elastic bond between both monomers is presented (middle top row of each panel). All the four defined angles (middle and bottom rows cyan: CG, grey AA), the unique dihedral (green: CG, grey: AA) and SASA (orange: CG, grey: AA) are plotted too.

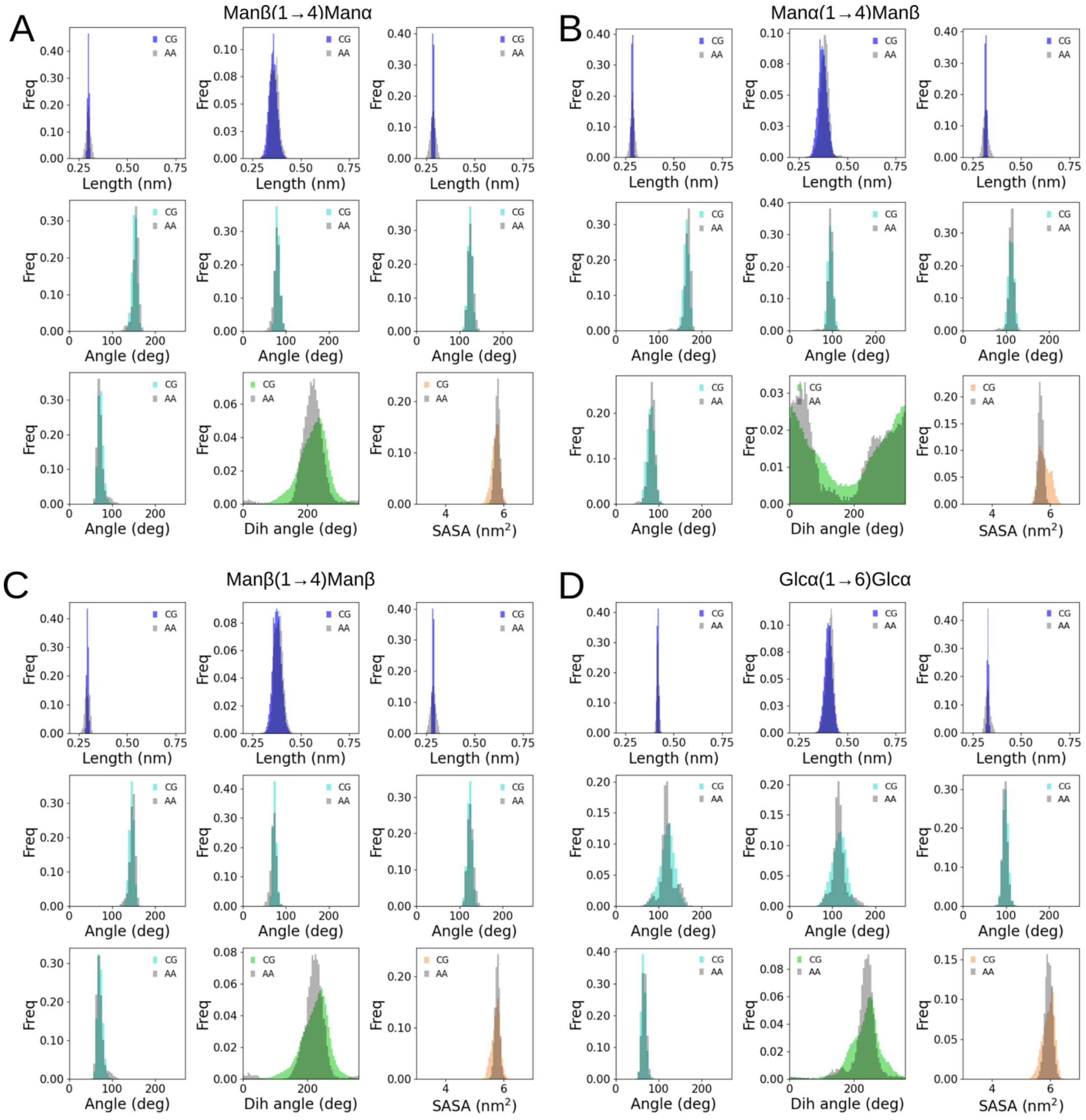

Figure S14: Comparative coarse-grained and all-atom distributions of bonded terms and SASA shown for 4 different disaccharides(A-D). Distributions from CG simulations are shown in colours and the ones coming from all-atom (GLYCAM-06j) simulations are shown in grey. For the sake of space, for each monosaccharide modelled as a ring, only 1 constraint (out of 3) between beads that define a ring is shown (first row of each panel- left and right). The elastic bond between both monomers is presented (middle top row of each panel). All the four defined angles (middle and bottom rows cyan: CG, grey AA), the unique dihedral (green: CG, grey: AA) and SASA (orange: CG, grey: AA) are plotted too.

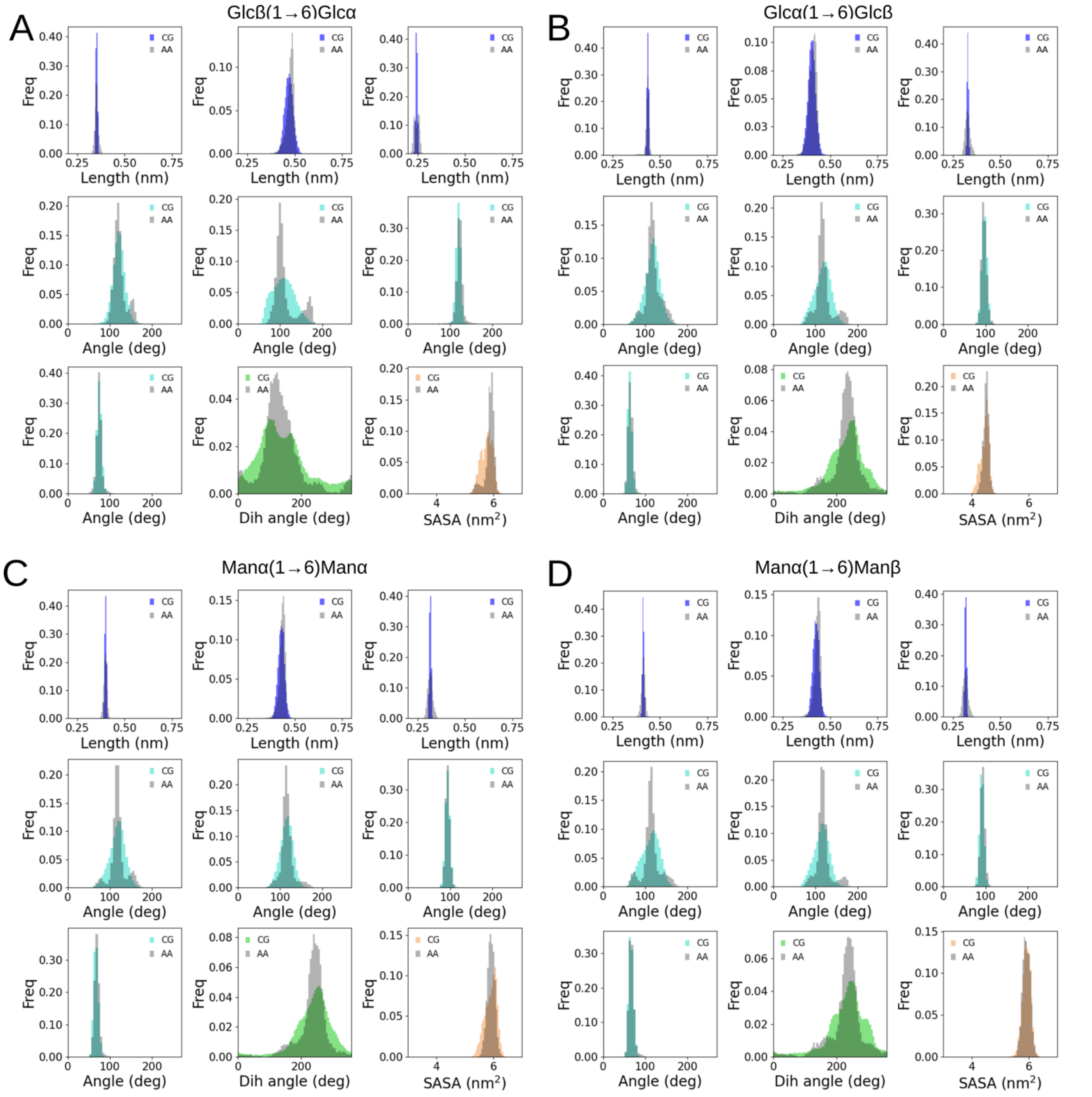

Figure S15: Comparative coarse-grained and all-atom distributions of bonded terms and SASA shown for 4 different disaccharides(A-D). Distributions from CG simulations are shown in colours and the ones coming from all-atom (GLYCAM-06j) simulations are shown in grey. For the sake of space, for each monosaccharide modelled as a ring, only 1 constraint (out of 3) between beads that define a ring is shown (first row of each panel- left and right). The elastic bond between both monomers is presented (middle top row of each panel). All the four defined angles (middle and bottom rows cyan: CG, grey AA), the unique dihedral (green: CG, grey: AA) and SASA (orange: CG, grey: AA) are plotted too.

	1GA_0GA						1GB_0GA					
	GLYCAM-06j		Martini 3		Overlaps		GLYCAM-06j		Martini 3		Overlaps	
	AVG	STD	AVG	STD	full	ref	AVG	STD	AVG	STD	full	ref
BOND0	0.32	0.02	0.32	0	0.374	0.544	0.3	0.01	0.3	0	0.347	0.515
BOND1	0.36	0.02	0.36	0	0.264	0.417	0.37	0.01	0.37	0	0.29	0.45
BOND2	0.45	0.02	0.45	0	0.305	0.467	0.45	0.01	0.45	0	0.361	0.531
BOND3	*0.42*	*0.02*	*0.41*	*0.03*	*0.656*	*0.792*	*0.43*	*0.02*	*0.42*	*0.02*	*0.688*	*0.815*
BOND4	0.3	0.01	0.3	0	0.342	0.51	0.33	0.01	0.33	0	0.361	0.531
BOND5	0.37	0.02	0.37	0	0.313	0.477	0.36	0.01	0.36	0	0.29	0.449
BOND6	0.47	0.01	0.47	0	0.357	0.526	0.47	0.01	0.47	0	0.327	0.493
ANG0	*111.57*	*11.13*	*113.37*	*12.42*	*0.575*	***0.73***	*153.19*	*7.95*	*150.69*	*6.62*	*0.655*	***0.791***
ANG1	*86.73*	*6.7*	*85.99*	*6.95*	*0.832*	***0.908***	*100.57*	*7.32*	*102.53*	*6.19*	*0.748*	***0.856***
ANG2	*142.47*	*8.58*	*140.13*	*6.8*	*0.684*	***0.812***	*104.43*	*7.79*	*105.01*	*8.34*	*0.804*	***0.891***
ANG3	*92.52*	*7.15*	*94.63*	*6.09*	*0.743*	***0.852***	*78.45*	*6.12*	*76.41*	*6.27*	*0.751*	***0.858***
DIH	*86.45*	*67.06*	*89.49*	*76.1*	*0.606*	***0.754***	*97.79*	*117.26*	*129.49*	*129.2*	*0.696*	***0.821***

	2GA_0GA						2GB_0GA					
	GLYCAM-06j		Martini 3		Overlaps		GLYCAM-06j		Martini 3		Overlaps	
	AVG	STD	AVG	STD	full	ref	AVG	STD	AVG	STD	full	ref
BOND0	0.32	0.01	0.32	0	0.508	0.674	0.32	0.01	0.32	0	0.49	0.658
BOND1	0.36	0.02	0.36	0	0.281	0.439	0.38	0.01	0.38	0	0.285	0.443
BOND2	0.41	0.01	0.41	0	0.326	0.492	0.42	0.01	0.42	0	0.333	0.499
BOND3	*0.37*	*0.02*	*0.36*	*0.02*	*0.739*	*0.85*	*0.37*	*0.02*	*0.36*	*0.02*	*0.646*	*0.785*
BOND4	0.33	0.01	0.33	0	0.35	0.518	0.31	0.03	0.31	0	0.083	0.153
BOND5	0.36	0.01	0.36	0	0.283	0.441	0.35	0.02	0.35	0	0.153	0.265
BOND6	0.47	0.01	0.47	0	0.327	0.493	0.45	0.03	0.45	0	0.075	0.139
ANG0	*122.7*	*8.86*	*122.01*	*8.4*	*0.735*	***0.847***	*113.69*	*10.74*	*114.01*	*8.87*	*0.701*	***0.824***
ANG1	*165.63*	*8.98*	*163.1*	*7.73*	*0.673*	***0.805***	*163.16*	*9.36*	*161.34*	*7.59*	*0.739*	***0.85***
ANG2	*106.91*	*6.32*	*108.02*	*6.46*	*0.83*	***0.907***	*115.11*	*12.95*	*116.94*	*10.45*	*0.516*	***0.681***
ANG3	*72.71*	*6.81*	*71.02*	*5.95*	*0.744*	***0.853***	*75.99*	*8.25*	*76.11*	*6.5*	*0.888*	***0.941***
DIH	*245.48*	*108.28*	*251.06*	*109.94*	*0.492*	***0.659***	*261.69*	*95.19*	*248.84*	*122.5*	*0.539*	***0.7***

	3GA_0GA						3GB_0GA					
	GLYCAM-06j		Martini 3		Overlaps		GLYCAM-06j		Martini 3		Overlaps	
	AVG	STD	AVG	STD	full	ref	AVG	STD	AVG	STD	full	ref
BOND0	0.33	0.01	0.33	0	0.53	0.692	0.32	0.01	0.32	0	0.461	0.631
BOND1	0.43	0.01	0.43	0	0.357	0.526	0.45	0.01	0.45	0	0.307	0.47
BOND2	0.33	0.01	0.33	0	0.296	0.457	0.34	0.01	0.34	0	0.296	0.457
BOND3	*0.36*	*0.03*	*0.34*	*0.03*	*0.595*	*0.746*	*0.42*	*0.03*	*0.42*	*0.02*	*0.764*	*0.866*
BOND4	0.33	0.01	0.33	0	0.351	0.519	0.45	0.02	0.45	0	0.298	0.459
BOND5	0.36	0.01	0.36	0	0.287	0.445	0.23	0.01	0.23	0	0.383	0.559
BOND6	0.47	0.01	0.47	0	0.328	0.493	0.23	0.01	0.23	0	0.284	0.445
ANG0	*108.2*	*9.71*	*107.42*	*8.65*	*0.837*	***0.911***	*110.5*	*8.03*	*108.38*	*7.22*	*0.743*	***0.852***
ANG1	*160.74*	*9.76*	*157.72*	*8.61*	*0.718*	***0.836***	*153.24*	*14.58*	*148.26*	*12.8*	*0.604*	***0.753***
ANG2	*112.85*	*9.19*	*113.19*	*9.2*	*0.86*	***0.925***	*79.15*	*6.77*	*79.38*	*5.23*	*0.863*	***0.927***
ANG3	*82.41*	*8.01*	*81.62*	*7.28*	*0.807*	***0.893***	*80.72*	*6.49*	*81.11*	*4.62*	*0.815*	***0.898***
DIH	*208.42*	*141.99*	*209.69*	*140.49*	*0.674*	***0.805***	*207.69*	*142.69*	*206.9*	*150.01*	*0.573*	***0.728***

Figure S16: Comparative coarse-grained and all-atom distributions of bonded terms for all the different disaccharides (part 1) Average values (AVG) are reported with their associated standard deviation (STD) for the data from reference all-atom simulations (GLYCAM-06j) and the Martini 3 parameters obtained in this work (Martini 3). Overlap values are reported for the full or reference overlap as explained in the Methods section. The reference overlap (ref) corresponds to the fraction of reference distribution that is sampled with the Martini 3 CG model. The names follow the nomenclature presented in Figure 1 from the main text. The numbers highlighted in grey correspond to the terms that are used to model a disaccharide that were not defined for a monosaccharide.

	1GA_0GB						1GB_0GB					
	GLYCAM-06j		Martini 3		Overlaps		GLYCAM-06j		Martini 3		Overlaps	
	AVG	STD	AVG	STD	full	ref	AVG	STD	AVG	STD	full	ref
BOND0	0.32	0.02	0.32	0	0.374	0.544	0.3	0.01	0.3	0	0.357	0.527
BOND1	0.36	0.02	0.36	0	0.264	0.417	0.37	0.01	0.37	0	0.289	0.448
BOND2	0.45	0.02	0.45	0	0.305	0.467	0.45	0.01	0.45	0	0.357	0.526
BOND3	*0.42*	*0.02*	*0.41*	*0.03*	*0.656*	*0.792*	*0.44*	*0.02*	*0.43*	*0.02*	*0.76*	*0.864*
BOND4	0.3	0.01	0.3	0	0.342	0.51	0.3	0.01	0.3	0	0.36	0.53
BOND5	0.37	0.02	0.37	0	0.313	0.477	0.38	0.01	0.38	0	0.291	0.451
BOND6	0.47	0.01	0.47	0	0.357	0.526	0.47	0.01	0.47	0	0.344	0.512
ANG0	*111.57*	*11.13*	*113.37*	*12.42*	*0.575*	***0.73***	*133.64*	*7.05*	*131.58*	*5.82*	*0.755*	***0.861***
ANG1	*86.73*	*6.7*	*85.99*	*6.95*	*0.832*	***0.908***	*81.55*	*6.08*	*81.95*	*5.55*	*0.956*	***0.978***
ANG2	*142.47*	*8.58*	*140.13*	*6.8*	*0.684*	***0.812***	*127.27*	*5.95*	*124.99*	*4.91*	*0.685*	***0.813***
ANG3	*92.52*	*7.15*	*94.63*	*6.09*	*0.743*	***0.852***	*76.83*	*5.3*	*77.18*	*4.78*	*0.937*	***0.967***
DIH	*86.45*	*67.06*	*89.49*	*76.1*	*0.606*	***0.754***	*243.01*	*18.77*	*244*	*18.59*	*0.832*	***0.908***

	2GA_0GB						2GB_0GB					
	GLYCAM-06j		Martini 3		Overlaps		GLYCAM-06j		Martini 3		Overlaps	
	AVG	STD	AVG	STD	full	ref	AVG	STD	AVG	STD	full	ref
BOND0	0.32	0.01	0.32	0	0.424	0.595	0.32	0.01	0.32	0	0.461	0.631
BOND1	0.36	0.02	0.36	0	0.266	0.42	0.37	0.01	0.37	0	0.299	0.46
BOND2	0.41	0.01	0.41	0	0.334	0.5	0.42	0.01	0.42	0	0.337	0.504
BOND3	*0.39*	*0.02*	*0.38*	*0.02*	*0.708*	*0.829*	*0.39*	*0.02*	*0.38*	*0.02*	*0.616*	*0.762*
BOND4	0.3	0.01	0.3	0	0.316	0.48	0.3	0.01	0.3	0	0.32	0.485
BOND5	0.37	0.02	0.37	0	0.29	0.45	0.37	0.01	0.37	0	0.29	0.449
BOND6	0.47	0.01	0.47	0	0.358	0.528	0.47	0.01	0.47	0	0.363	0.532
ANG0	*97.43*	*9.05*	*98*	*7.84*	*0.769*	***0.87***	*86.87*	*8.84*	*87.05*	*7.34*	*0.838*	***0.912***
ANG1	*151.64*	*9.85*	*148.68*	*7.99*	*0.618*	***0.764***	*142.13*	*8.23*	*139.37*	*6.85*	*0.72*	***0.837***
ANG2	*117.28*	*7.48*	*114.12*	*5.7*	*0.752*	***0.859***	*123.19*	*6.65*	*121.45*	*5.34*	*0.805*	***0.892***
ANG3	*66.49*	*7.06*	*66.92*	*5.12*	*0.707*	***0.828***	*72.42*	*6.36*	*73.3*	*5.31*	*0.788*	***0.881***
DIH	*201.27*	*38.42*	*200.95*	*40.79*	*0.696*	***0.821***	*222.03*	*49.12*	*230.86*	*56.52*	*0.484*	***0.652***

	3GA_0GB						3GB_0GB					
	GLYCAM-06j		Martini 3		Overlaps		GLYCAM-06j		Martini 3		Overlaps	
	AVG	STD	AVG	STD	full	ref	AVG	STD	AVG	STD	full	ref
BOND0	0.3	0.01	0.3	0	0.518	0.682	0.33	0.01	0.33	0	0.423	0.594
BOND1	0.43	0.01	0.43	0	0.324	0.49	0.45	0.01	0.45	0	0.349	0.518
BOND2	0.33	0.01	0.33	0	0.378	0.548	0.35	0.01	0.35	0	0.345	0.513
BOND3	*0.39*	*0.02*	*0.39*	*0.02*	*0.573*	*0.728*	*0.38*	*0.02*	*0.37*	*0.02*	*0.747*	*0.855*
BOND4	0.3	0.01	0.3	0	0.321	0.487	0.3	0.01	0.3	0	0.317	0.482
BOND5	0.37	0.02	0.37	0	0.298	0.459	0.37	0.02	0.37	0	0.292	0.451
BOND6	0.47	0.01	0.47	0	0.364	0.533	0.47	0.01	0.47	0	0.359	0.529
ANG0	*87*	*8.78*	*87.19*	*7.75*	*0.693*	***0.819***	*87.6*	*7.62*	*86.93*	*6.7*	*0.729*	***0.843***
ANG1	*161.93*	*14.41*	*156.24*	*11.35*	*0.527*	***0.69***	*161.78*	*10.7*	*158.69*	*8.45*	*0.664*	***0.798***
ANG2	*117.9*	*8.94*	*113.86*	*6.68*	*0.634*	***0.776***	*115.41*	*7.79*	*112.99*	*5.82*	*0.775*	***0.873***
ANG3	*67.93*	*8.95*	*67.88*	*6.53*	*0.779*	***0.876***	*65.09*	*7.39*	*66.32*	*5.19*	*0.693*	***0.819***
DIH	*212.52*	*46.62*	*220.95*	*55.04*	*0.504*	***0.67***	*215.86*	*36.39*	*218.19*	*39.55*	*0.744*	***0.853***

Figure S17: Comparative coarse-grained and all-atom distributions of bonded terms for all the different disaccharides (part 2) Average values (AVG) are reported with their associated standard deviation (STD) for the data from reference all-atom simulations (GLYCAM-06j) and the Martini 3 parameters obtained in this work (Martini 3). Overlap values are reported for the full or reference overlap as explained in the Methods section. The reference overlap (ref) corresponds to the fraction of reference distribution that is sampled with the Martini 3 CG model. The names follow the nomenclature presented in Figure 1 from the main text. The numbers highlighted in grey correspond to the terms that are used to model a disaccharide that were not defined for a monosaccharide.

	1MA_0MA						1MB_0MA					
	GLYCAM-06j		Martini 3		Overlaps		GLYCAM-06j		Martini 3		Overlaps	
	AVG	STD	AVG	STD	full	ref	AVG	STD	AVG	STD	full	ref
BOND0	0.32	0.01	0.32	0	0.403	0.575	0.29	0.01	0.29	0	0.378	0.549
BOND1	0.36	0.01	0.36	0	0.289	0.448	0.37	0.01	0.37	0	0.319	0.484
BOND2	0.43	0.02	0.43	0	0.302	0.464	0.42	0.01	0.42	0	0.305	0.468
BOND3	*0.53*	*0.01*	*0.53*	*0.01*	*0.681*	*0.811*	*0.47*	*0.02*	*0.46*	*0.03*	*0.586*	*0.739*
BOND4	0.32	0.01	0.32	0	0.354	0.523	0.32	0.01	0.32	0	0.369	0.539
BOND5	0.36	0.01	0.36	0	0.296	0.457	0.36	0.01	0.36	0	0.283	0.441
BOND6	0.45	0.02	0.45	0	0.252	0.403	0.45	0.02	0.45	0	0.268	0.423
ANG0	*93.21*	*6.48*	*94.02*	*6.9*	*0.812*	***0.896***	*148.92*	*8.71*	*147.8*	*7.91*	*0.792*	***0.884***
ANG1	*66.35*	*7.53*	*62.29*	*6.92*	*0.616*	***0.762***	*101.64*	*9.82*	*101.88*	*9.08*	*0.657*	***0.793***
ANG2	*88.97*	*6.14*	*89.59*	*6.42*	*0.834*	***0.91***	*93.68*	*6.88*	*93.31*	*7.61*	*0.727*	***0.842***
ANG3	*63.28*	*6.86*	*59.71*	*6.12*	*0.613*	***0.76***	*74.97*	*5.88*	*72.74*	*6.08*	*0.737*	***0.849***
DIH	*202.93*	*22*	*202.78*	*22.87*	*0.653*	***0.79***	*75.16*	*31.28*	*75.39*	*39.44*	*0.743*	***0.853***

	2MA_0MA						2MB_0MA					
	GLYCAM-06j		Martini 3		Overlaps		GLYCAM-06j		Martini 3		Overlaps	
	AVG	STD	AVG	STD	full	ref	AVG	STD	AVG	STD	full	ref
BOND0	0.32	0.01	0.32	0	0.314	0.478	0.32	0.01	0.32	0	0.463	0.633
BOND1	0.35	0.02	0.35	0	0.169	0.289	0.37	0.01	0.37	0	0.322	0.487
BOND2	0.4	0.02	0.4	0	0.215	0.354	0.42	0.01	0.42	0	0.321	0.486
BOND3	*0.44*	*0.02*	*0.44*	*0.02*	*0.748*	*0.856*	*0.45*	*0.02*	*0.45*	*0.02*	*0.566*	*0.723*
BOND4	0.32	0.01	0.32	0	0.371	0.541	0.32	0.01	0.32	0	0.373	0.544
BOND5	0.36	0.01	0.36	0	0.289	0.449	0.36	0.01	0.36	0	0.287	0.446
BOND6	0.44	0.02	0.44	0	0.272	0.428	0.44	0.02	0.44	0	0.257	0.409
ANG0	*91.12*	*9.02*	*88.7*	*9.23*	*0.763*	***0.866***	*79.63*	*9.81*	*76.74*	*8.64*	*0.64*	***0.781***
ANG1	*122.26*	*14.07*	*121.91*	*13.01*	*0.752*	***0.859***	*106.62*	*9.76*	*106.66*	*10.16*	*0.826*	***0.905***
ANG2	*88.03*	*6.35*	*87.93*	*6.6*	*0.882*	***0.937***	*88.47*	*7.07*	*89.04*	*7.54*	*0.776*	***0.874***
ANG3	*59.2*	*6.12*	*61.11*	*4.31*	*0.653*	***0.79***	*61.12*	*6.71*	*61.28*	*4.95*	*0.865*	***0.928***
DIH	*184.4*	*47.56*	*185.39*	*61.04*	*0.621*	***0.766***	*178.42*	*36.35*	*174.89*	*43.66*	*0.55*	***0.71***

	3MA_0MA						3MB_0MA					
	GLYCAM-06j		Martini 3		Overlaps		GLYCAM-06j		Martini 3		Overlaps	
	AVG	STD	AVG	STD	full	ref	AVG	STD	AVG	STD	full	ref
BOND0	0.32	0.01	0.32	0	0.44	0.611	0.35	0.01	0.35	0	0.332	0.498
BOND1	0.4	0.02	0.4	0	0.306	0.469	0.42	0.01	0.42	0	0.302	0.463
BOND2	0.32	0.01	0.32	0	0.317	0.482	0.32	0.01	0.32	0	0.456	0.626
BOND3	*0.43*	*0.02*	*0.78*	*0.01*	*0*	*0*	*0.39*	*0.02*	*0.38*	*0.02*	*0.623*	*0.768*
BOND4	0.44	0.02	0.4	0	0.02	0.039	0.32	0.01	0.32	0	0.369	0.539
BOND5	0.37	0.01	0.3	0	0	0	0.36	0.01	0.36	0	0.284	0.443
BOND6	0.3	0.01	0.15	0	0	0	0.45	0.02	0.45	0	0.249	0.399
ANG0	*96.11*	*7.12*	*106.31*	*5.22*	*0.216*	***0.355***	*164.76*	*8.65*	*161.9*	*6.91*	*0.629*	***0.772***
ANG1	*90.42*	*30.5*	*159.73*	*8.7*	*0.067*	***0.126***	*110.28*	*9.15*	*110.74*	*7.47*	*0.687*	***0.815***
ANG2	*78.67*	*7.91*	*119.13*	*3.75*	*0.001*	***0.002***	*102.49*	*6.21*	*104.01*	*6.66*	*0.826*	***0.904***
ANG3	*110.32*	*6.79*	*121.64*	*4.67*	*0.178*	***0.302***	*79.85*	*5.41*	*77.98*	*5.06*	*0.732*	***0.845***
DIH	*227.84*	*142.12*	*236.42*	*125.28*	*0.546*	***0.706***	*153.71*	*51.65*	*160.42*	*54.31*	*0.702*	***0.825***

Figure S18: Comparative coarse-grained and all-atom distributions of bonded terms for all the different disaccharides (part 3) Average values (AVG) are reported with their associated standard deviation (STD) for the data from reference all-atom simulations (GLYCAM-06j) and the Martini 3 parameters obtained in this work (Martini 3). Overlap values are reported for the full or reference overlap as explained in the Methods section. The reference overlap (ref) corresponds to the fraction of reference distribution that is sampled with the Martini 3 CG model. The names follow the nomenclature presented in Figure 1 from the main text. The numbers highlighted in grey correspond to the terms that are used to model a disaccharide that were not defined for a monosaccharide.

	1MA_0MB						1MB_0MB					
	GLYCAM-06j		Martini 3		Overlaps		GLYCAM-06j		Martini 3		Overlaps	
	AVG	STD	AVG	STD	full	ref	AVG	STD	AVG	STD	full	ref
BOND0	0.32	0.01	0.32	0	0.404	0.576	0.29	0.01	0.29	0	0.362	0.532
BOND1	0.36	0.01	0.36	0	0.286	0.445	0.37	0.01	0.37	0	0.316	0.48
BOND2	0.43	0.01	0.43	0	0.311	0.475	0.43	0.01	0.43	0	0.289	0.448
BOND3	*0.46*	*0.03*	*0.45*	*0.03*	*0.541*	*0.702*	*0.42*	*0.02*	*0.41*	*0.02*	*0.76*	*0.863*
BOND4	0.29	0.01	0.29	0	0.37	0.54	0.29	0.01	0.29	0	0.348	0.516
BOND5	0.37	0.01	0.37	0	0.315	0.48	0.37	0.01	0.37	0	0.315	0.479
BOND6	0.44	0.02	0.44	0	0.267	0.421	0.44	0.02	0.44	0	0.245	0.394
ANG0	*98.64*	*8.57*	*98.23*	*9.41*	*0.643*	***0.783***	*151.63*	*7.92*	*148.54*	*6.7*	*0.595*	***0.746***
ANG1	*81.27*	*6.5*	*79.47*	*6.78*	*0.806*	***0.893***	*94.45*	*8.18*	*96.7*	*6.86*	*0.825*	***0.904***
ANG2	*141.33*	*9.16*	*140.55*	*8.25*	*0.733*	***0.846***	*142.75*	*7.01*	*140.47*	*5.98*	*0.688*	***0.815***
ANG3	*95.64*	*9.52*	*95.84*	*8.61*	*0.669*	***0.801***	*89.07*	*7.4*	*89.59*	*6.35*	*0.89*	***0.942***
DIH	*94.15*	*62.04*	*86.91*	*64.01*	*0.562*	***0.72***	*278.54*	*31.48*	*281.19*	*42.85*	*0.785*	***0.88***

	2MA_0MB						2MB_0MB					
	GLYCAM-06j		Martini 3		Overlaps		GLYCAM-06j		Martini 3		Overlaps	
	AVG	STD	AVG	STD	full	ref	AVG	STD	AVG	STD	full	ref
BOND0	0.32	0.01	0.32	0	0.503	0.669	0.31	0.01	0.31	0	0.554	0.713
BOND1	0.36	0.01	0.36	0	0.286	0.445	0.37	0.01	0.37	0	0.311	0.474
BOND2	0.41	0.01	0.41	0	0.358	0.527	0.42	0.01	0.42	0	0.347	0.515
BOND3	*0.38*	*0.02*	*0.37*	*0.02*	*0.549*	*0.709*	*0.38*	*0.03*	*0.36*	*0.03*	*0.438*	*0.61*
BOND4	0.29	0.01	0.29	0	0.368	0.538	0.29	0.01	0.29	0	0.357	0.526
BOND5	0.37	0.01	0.37	0	0.33	0.497	0.37	0.01	0.37	0	0.334	0.501
BOND6	0.44	0.02	0.44	0	0.271	0.426	0.44	0.02	0.44	0	0.258	0.411
ANG0	*92.97*	*6.84*	*93.47*	*7*	*0.748*	***0.856***	*92.95*	*7.45*	*92.01*	*7.12*	*0.781*	***0.877***
ANG1	*101.73*	*9.46*	*102.36*	*8.85*	*0.789*	***0.882***	*102.73*	*10.22*	*103.93*	*10.16*	*0.652*	***0.789***
ANG2	*128.34*	*8.97*	*130.12*	*8.74*	*0.642*	***0.782***	*132.07*	*10.31*	*132.22*	*9.58*	*0.621*	***0.766***
ANG3	*85.07*	*10.02*	*84.97*	*8.63*	*0.854*	***0.921***	*89.28*	*10.63*	*88.66*	*8.31*	*0.809*	***0.894***
DIH	*101.16*	*45.29*	*94.27*	*37.02*	*0.654*	***0.791***	*89.92*	*57.78*	*77.74*	*61.87*	*0.522*	***0.686***

	3MA_0MB						3MB_0MB					
	GLYCAM-06j		Martini 3		Overlaps		GLYCAM-06j		Martini 3		Overlaps	
	AVG	STD	AVG	STD	full	ref	AVG	STD	AVG	STD	full	ref
BOND0	0.32	0.01	0.32	0	0.432	0.603	0.32	0.01	0.32	0	0.421	0.592
BOND1	0.41	0.01	0.41	0	0.3	0.461	0.42	0.01	0.42	0	0.31	0.474
BOND2	0.33	0.01	0.33	0	0.338	0.506	0.35	0.01	0.35	0	0.319	0.484
BOND3	*0.39*	*0.02*	*0.38*	*0.02*	*0.69*	*0.816*	*0.37*	*0.02*	*0.36*	*0.02*	*0.665*	*0.799*
BOND4	0.29	0.01	0.29	0	0.354	0.523	0.29	0.01	0.29	0	0.346	0.514
BOND5	0.37	0.01	0.34	0	0.043	0.083	0.37	0.01	0.37	0	0.303	0.465
BOND6	0.44	0.02	0.42	0	0.139	0.245	0.44	0.02	0.44	0	0.255	0.406
ANG0	*88.44*	*6*	*88.19*	*5.34*	*0.86*	***0.924***	*94.56*	*6.56*	*93.62*	*6.31*	*0.78*	***0.876***
ANG1	*162.08*	*7.33*	*159.98*	*5.86*	*0.675*	***0.806***	*163.44*	*7.79*	*162.29*	*7.31*	*0.806*	***0.893***
ANG2	*132.75*	*9.34*	*128*	*7.3*	*0.591*	***0.743***	*132.62*	*8.67*	*128.84*	*6.64*	*0.616*	***0.763***
ANG3	*77.27*	*8.87*	*80.24*	*7.35*	*0.697*	***0.821***	*77.34*	*8.06*	*77.27*	*6.5*	*0.769*	***0.87***
DIH	*241.6*	*32.69*	*249.96*	*37.72*	*0.643*	***0.783***	*242.52*	*33.59*	*243.99*	*38.87*	*0.621*	***0.766***

Figure S19: Comparative coarse-grained and all-atom distributions of bonded terms for all the different disaccharides (part 4) Average values (AVG) are reported with their associated standard deviation (STD) for the data from reference all-atom simulations (GLYCAM-06j) and the Martini 3 parameters obtained in this work (Martini 3). Overlap values are reported for the full or reference overlap as explained in the Methods section. The reference overlap (ref) corresponds to the fraction of reference distribution that is sampled with the Martini 3 CG model. The names follow the nomenclature presented in Figure 1 from the main text. The numbers highlighted in grey correspond to the terms that are used to model a disaccharide that were not defined for a monosaccharide.

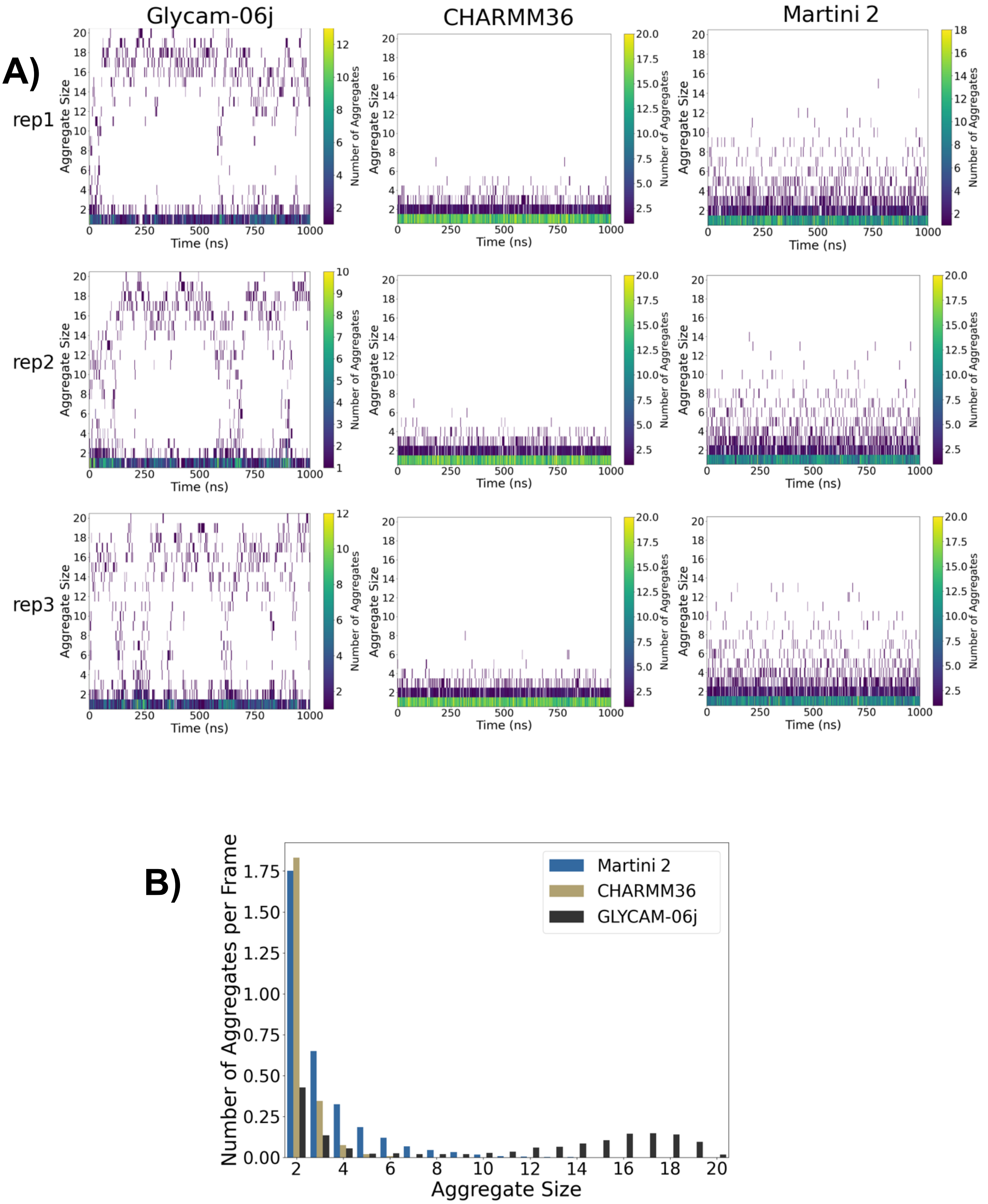

Figure S20: **Aggregation data for the 1GA_0GA disaccharide using the Martini2 coarse-grained forcefield (CG).** Reference data is also shown from all-atom simulations using the GLYCAM-06j forcefield and CHARMM36. **(A)** Size of aggregates present at each frame of each replica (rep) trajectory, the number of aggregates is represented by the colour map. Data is provided for each replica trajectory individually. **(B)** Distribution of aggregate sizes during the last 500 ns of all three replica trajectories.

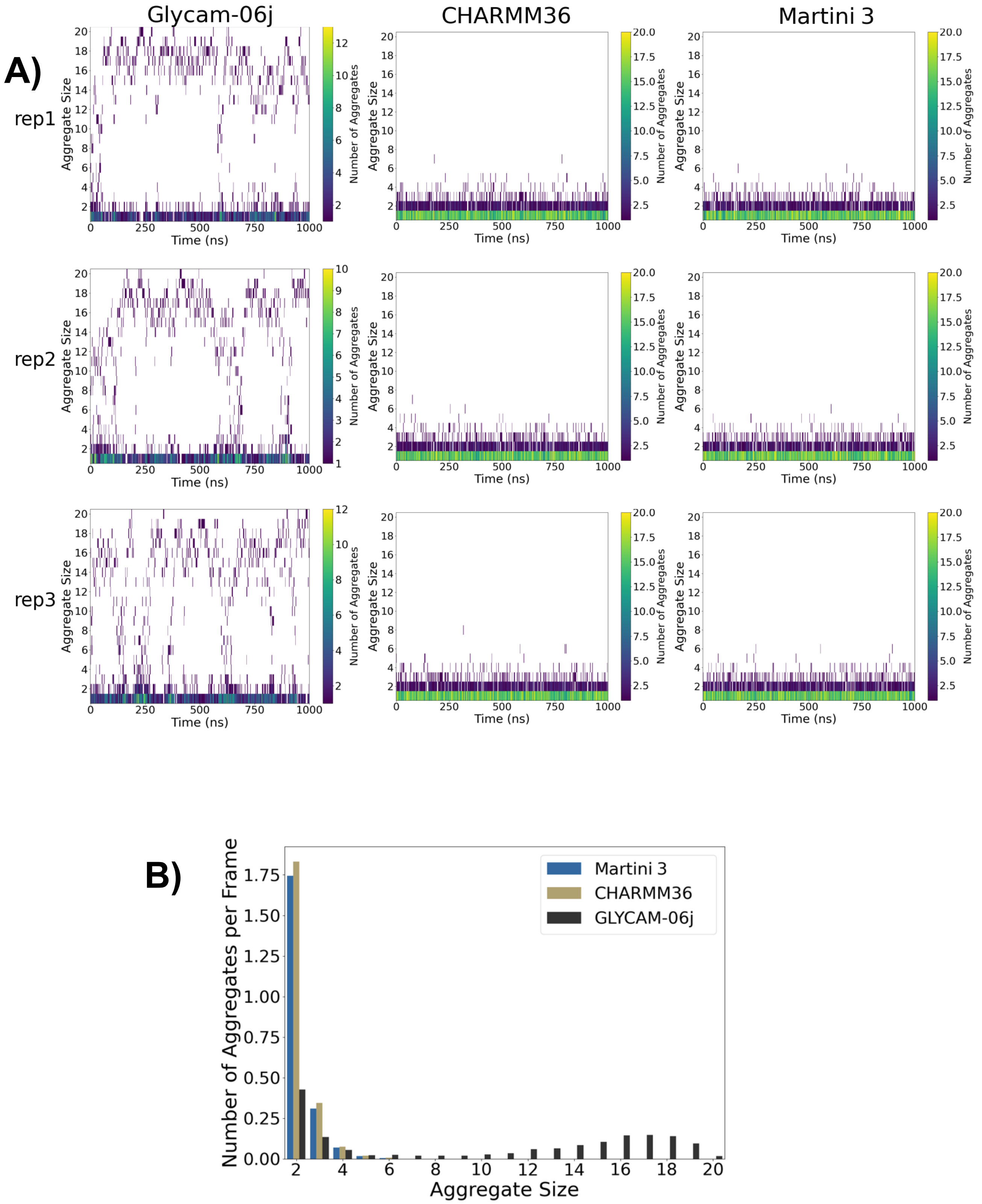

Figure S21: Aggregation data for the 1GA_0GA disaccharide using the Martini 3 coarse-grained forcefield (CG). Reference data is also shown from all-atom simulations using the GLYCAM-06j forcefield and CHARMM36. (**A)** Size of aggregates present at each frame of each replica (rep) trajectory, the number of aggregates is represented by the colour map. Data is provided for each replica trajectory individually. **(B)** Distribution of aggregate sizes during the last 500 ns of all three replica trajectories.

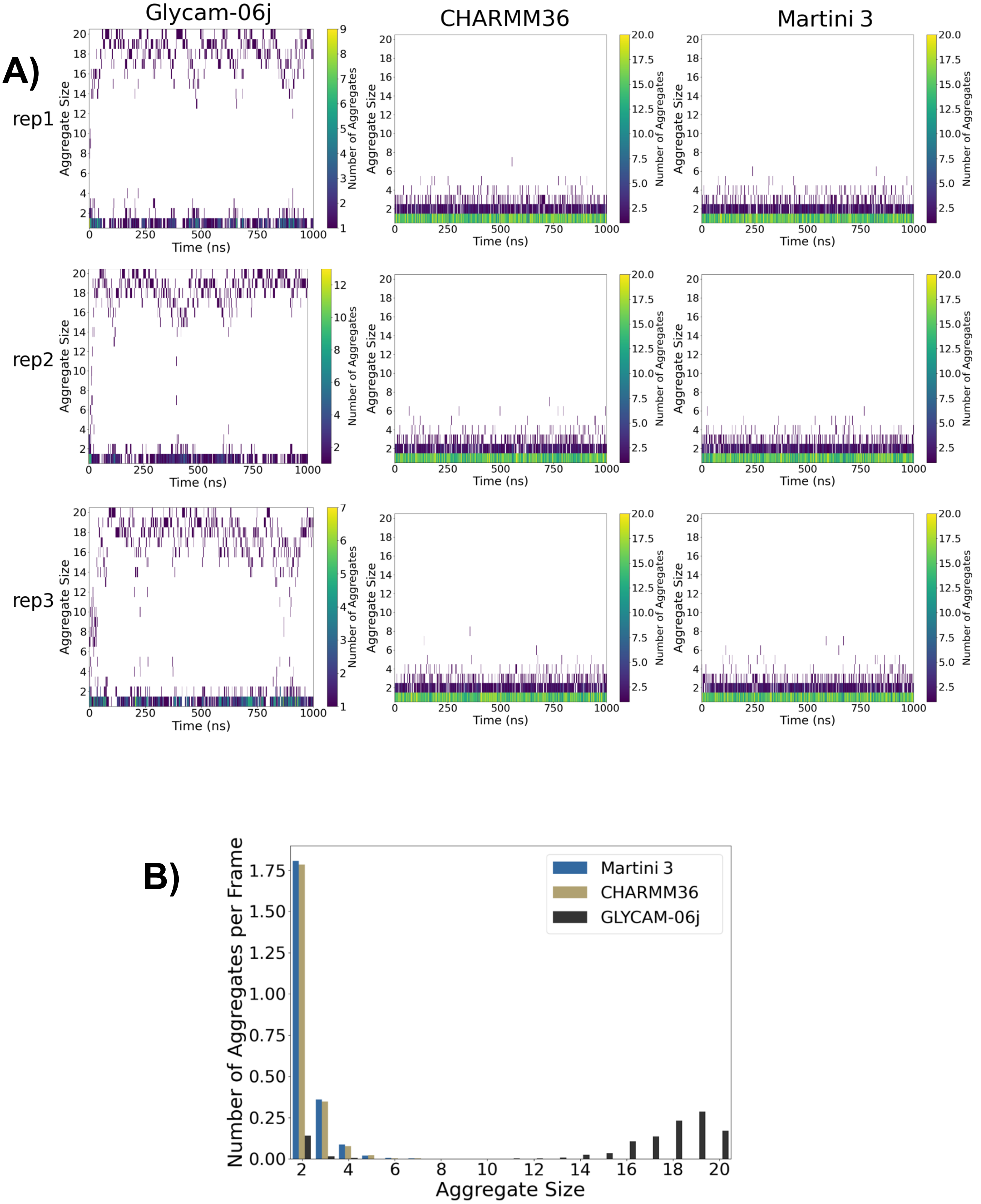

Figure S22: Aggregation data for the 1GB_0GB disaccharide using the Martini 3 coarse-grained forcefield (CG). Reference data is also shown from all-atom simulations using the GLYCAM-06j forcefield and CHARMM36. (**A**) Size of aggregates present at each frame of each replica (rep) trajectory, the number of aggregates is represented by the colour map. Data is provided for each replica trajectory individually. **(B)** Distribution of aggregate sizes during the last 500 ns of all three replica trajectories.

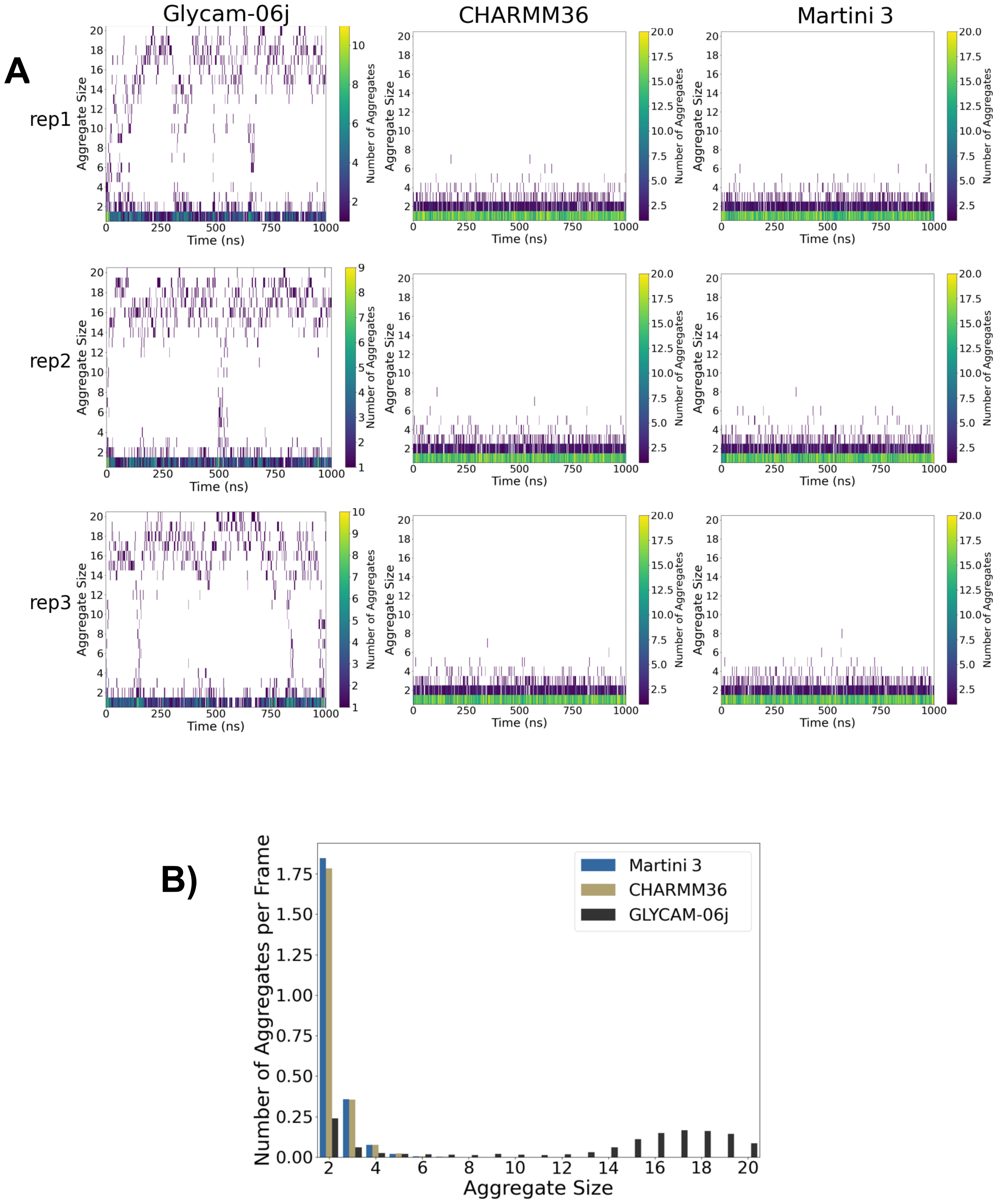

Figure S23: Aggregation data for the 2MA_0MA disaccharide using the Martini 3 coarse-grained forcefield (CG). Reference data is also shown from all-atom simulations using the GLYCAM-06j forcefield and CHARMM36. **(A)** Size of aggregates present at each frame of each replica (rep) trajectory, the number of aggregates is represented by the colour map. Data is provided for each replica trajectory individually. **(B)** Distribution of aggregate sizes during the last 500 ns of all three replica trajectories.

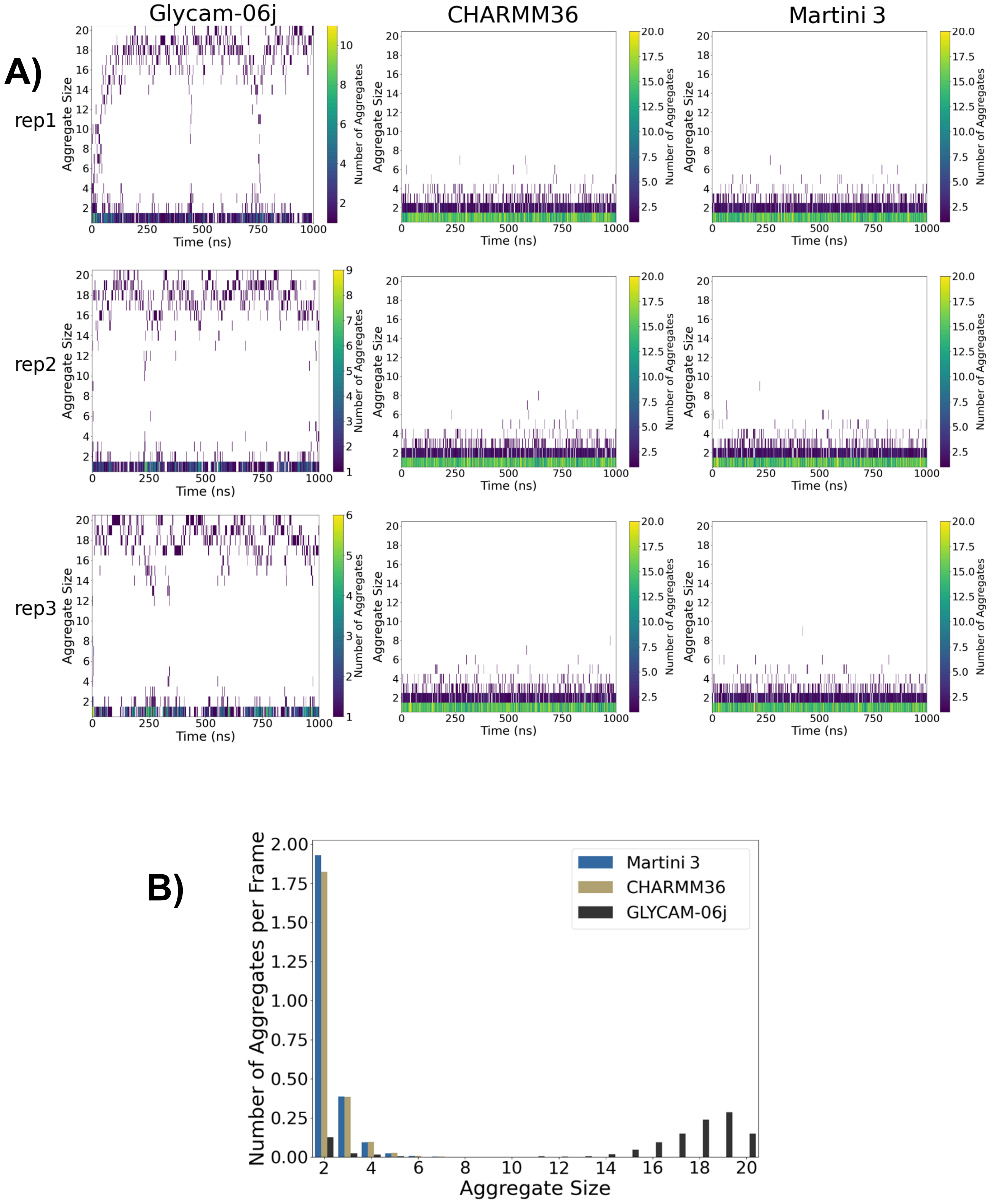

Figure S24: Aggregation data for the 4MA_0MA disaccharide using the Martini 3 coarse-grained forcefield (CG). Reference data is also shown from all-atom simulations using the GLYCAM-06j forcefield and CHARMM36. (**A**) Size of aggregates present at each frame of each replica (rep) trajectory, the number of aggregates is represented by the colour map. Data is provided for each replica trajectory individually. **(B)** Distribution of aggregate sizes during the last 500 ns of all three replica trajectories.

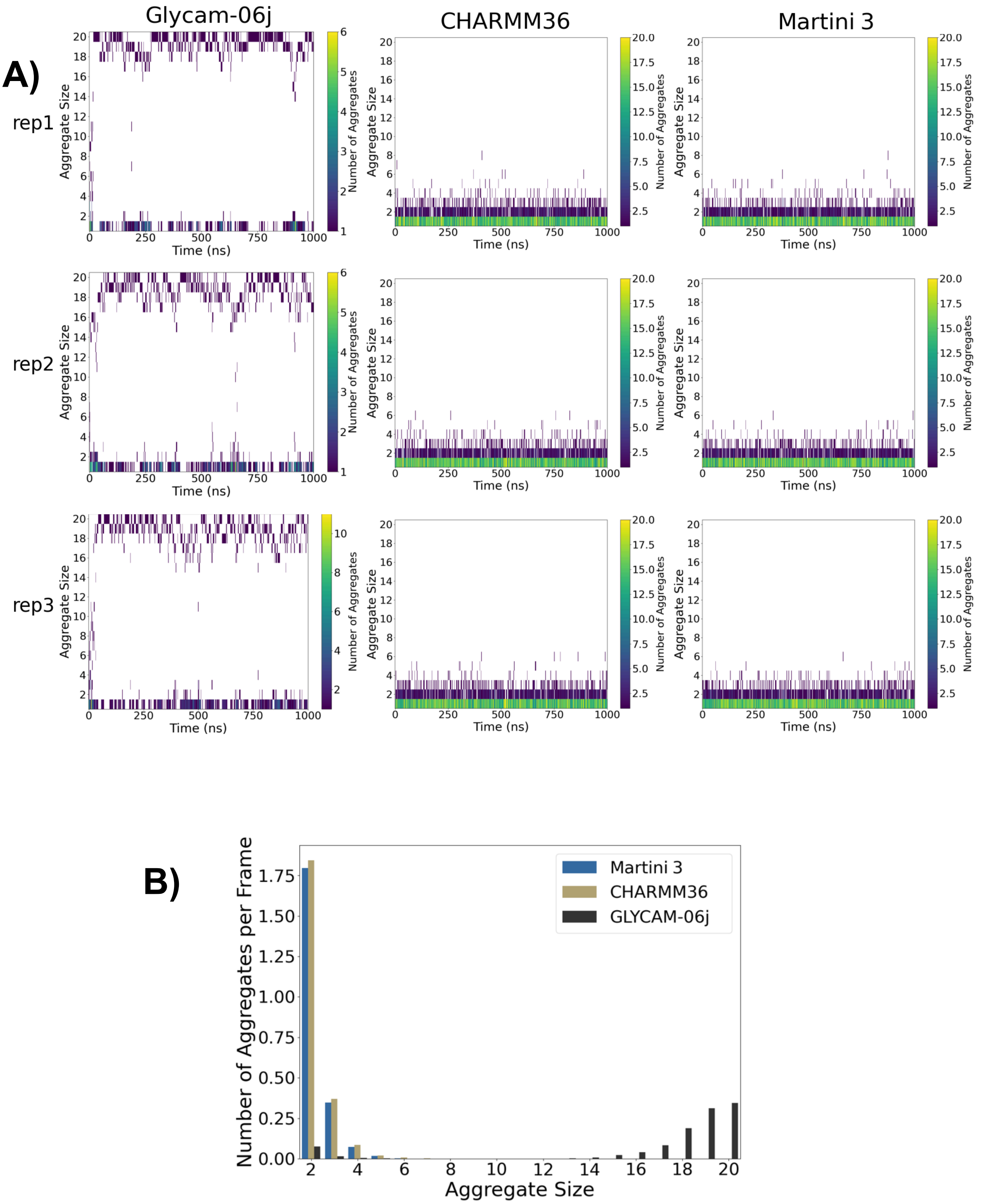

Figure S25: Aggregation data for the 6GB_0GB disaccharide using the Martini 3 coarse-grained forcefield (CG). Reference data is also shown from all-atom simulations using the GLYCAM-06j forcefield and CHARMM36. (**A**) Size of aggregates present at each frame of each replica (rep) trajectory, the number of aggregates is represented by the colour map. Data is provided for each replica trajectory individually. **(B)** Distribution of aggregate sizes during the last 500 ns of all three replica trajectories.

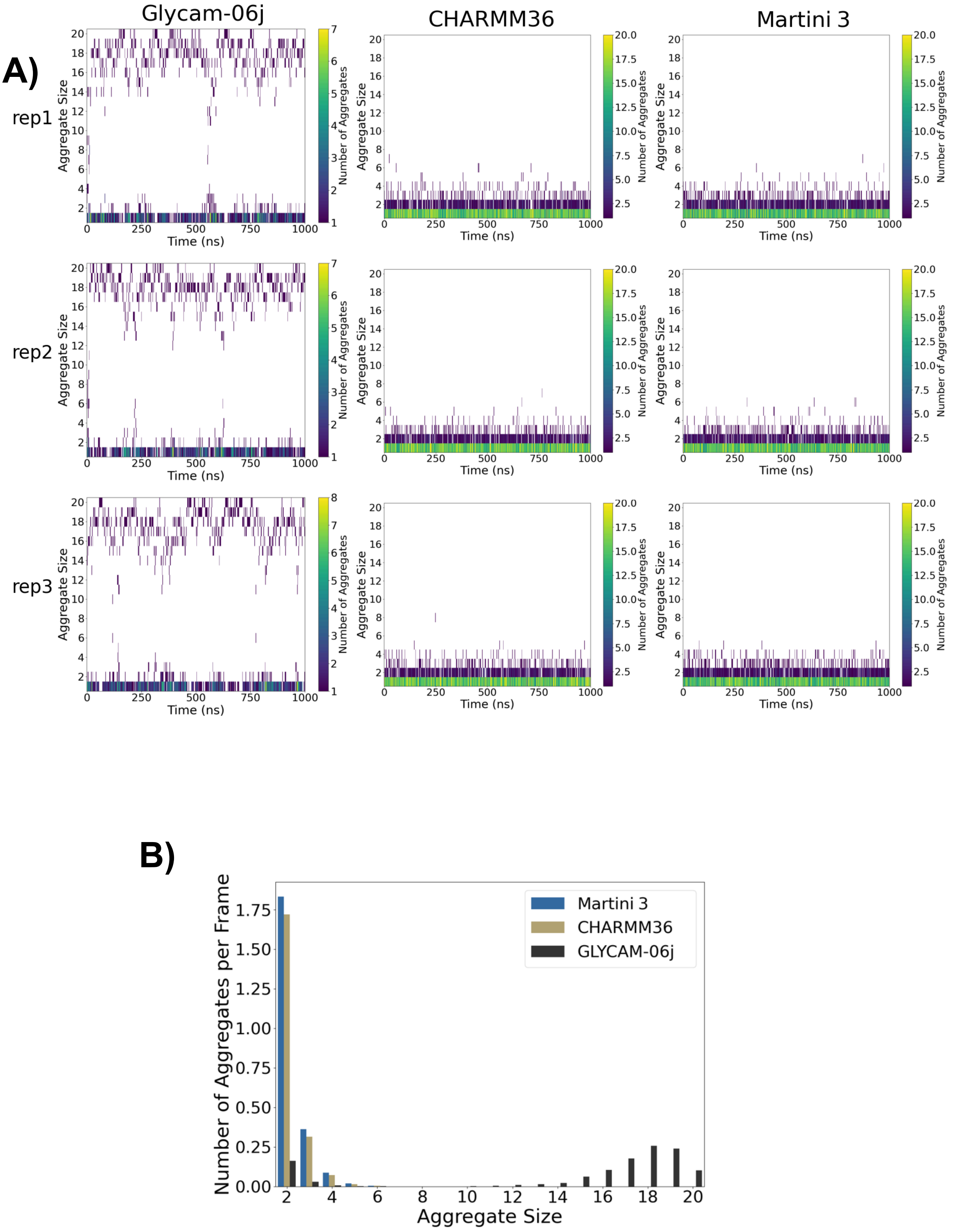

Figure S26: Aggregation data for the 6MB_0MB disaccharide using the Martini 3 coarse-grained forcefield (CG). Reference data is also shown from all-atom simulations using the GLYCAM-06j forcefield and CHARMM36. (**A**) Size of aggregates present at each frame of each replica (rep) trajectory, the number of aggregates is represented by the colour map. Data is provided for each replica trajectory individually. **(B)** Distribution of aggregate sizes during the last 500 ns of all three replica trajectories.

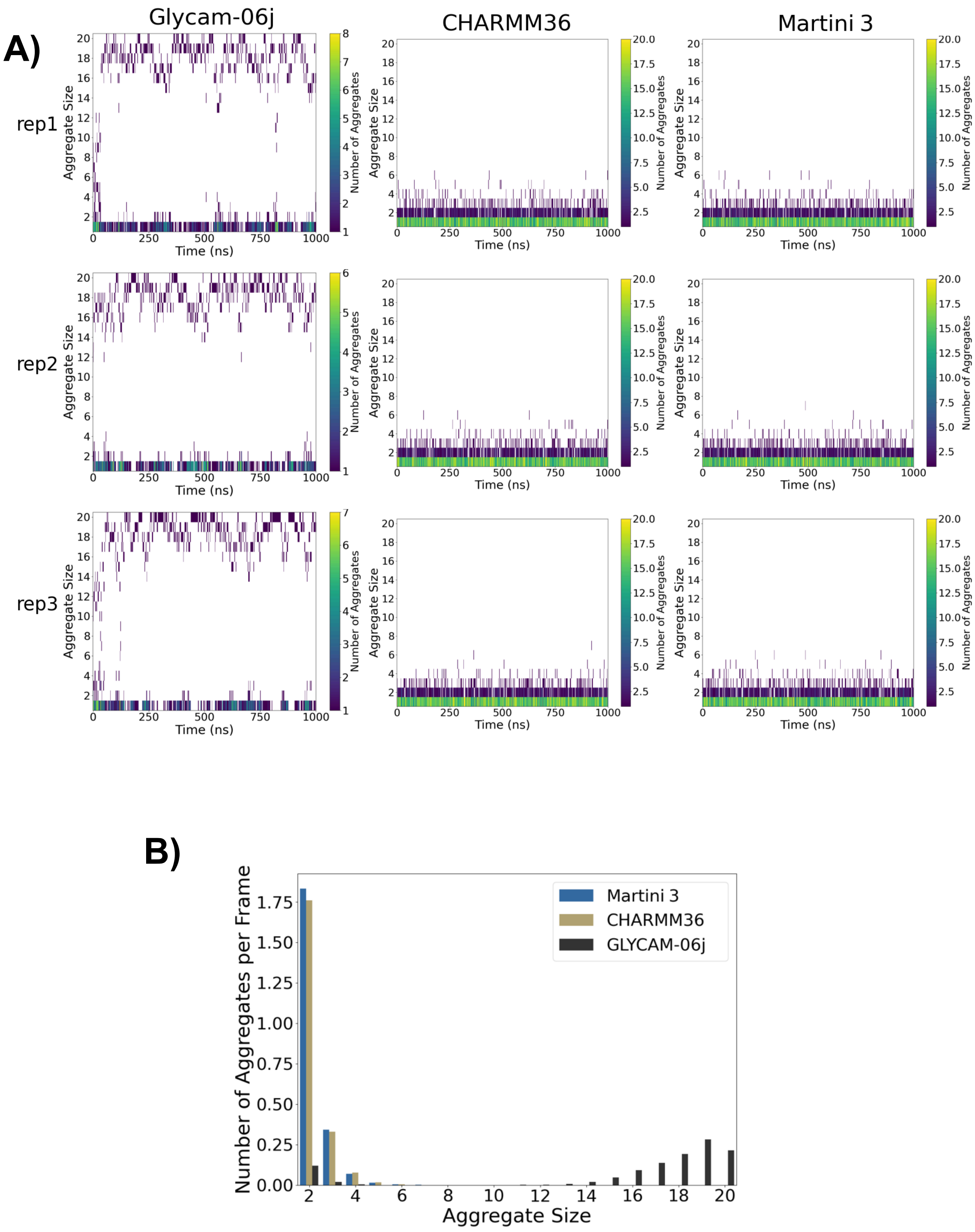

Figure S27: Aggregation data for the 6MA_0MB disaccharide using the Martini 3 coarse-grained forcefield (CG). Reference data is also shown from all-atom simulations using the GLYCAM-06j forcefield and CHARMM36. (**A**) Size of aggregates present at each frame of each replica (rep) trajectory, the number of aggregates is represented by the colour map. Data is provided for each replica trajectory individually. **(B)** Distribution of aggregate sizes during the last 500 ns of all three replica trajectories.

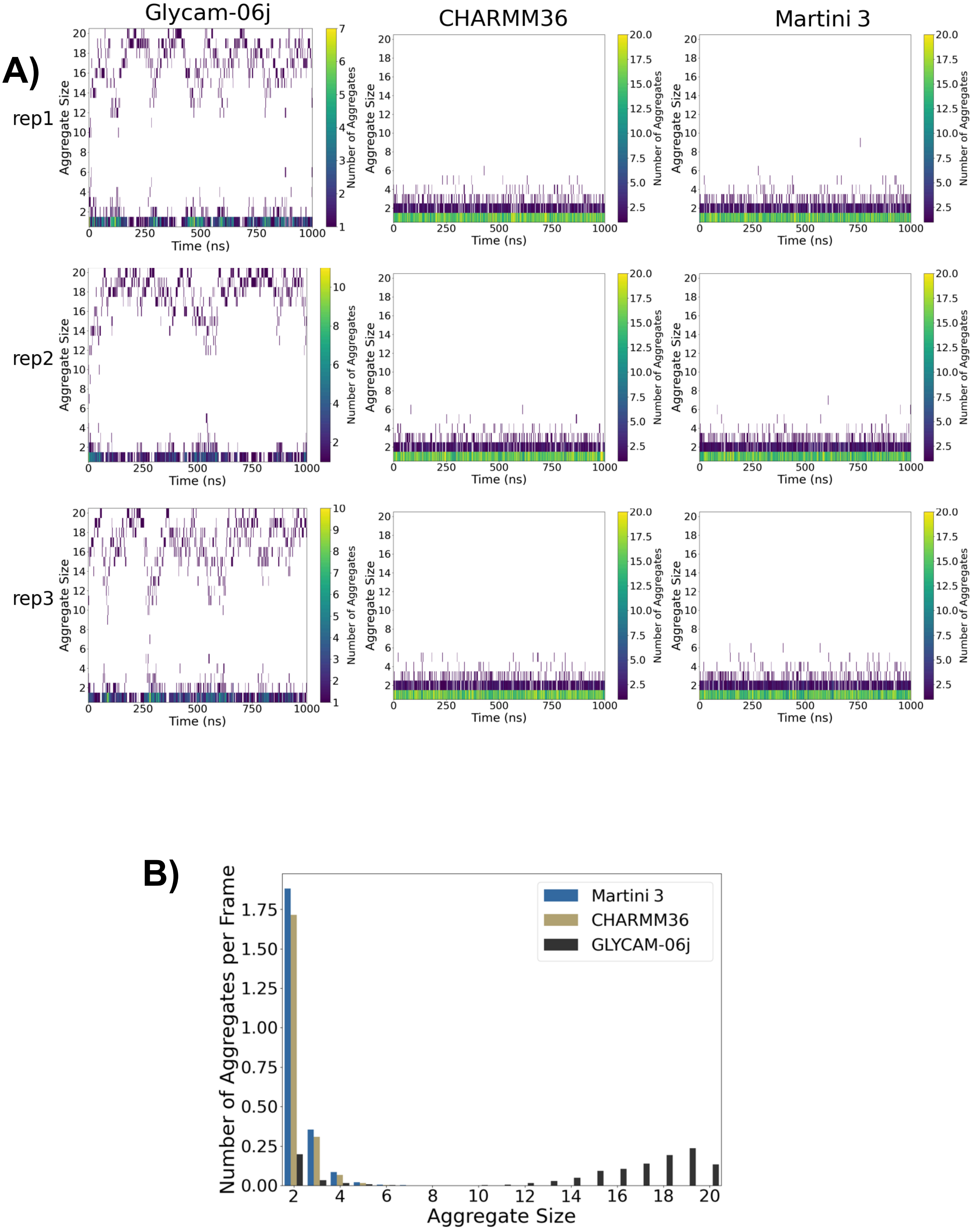

Figure S28: Aggregation data for the 3GB_0GB disaccharide using the Martini 3 coarse-grained forcefield (CG). Reference data is also shown from all-atom simulations using the GLYCAM-06j forcefield and CHARMM36. (**A**) Size of aggregates present at each frame of each replica (rep) trajectory, the number of aggregates is represented by the colour map. Data is provided for each replica trajectory individually. **(B)** Distribution of aggregate sizes during the last 500 ns of all three replica trajectories.

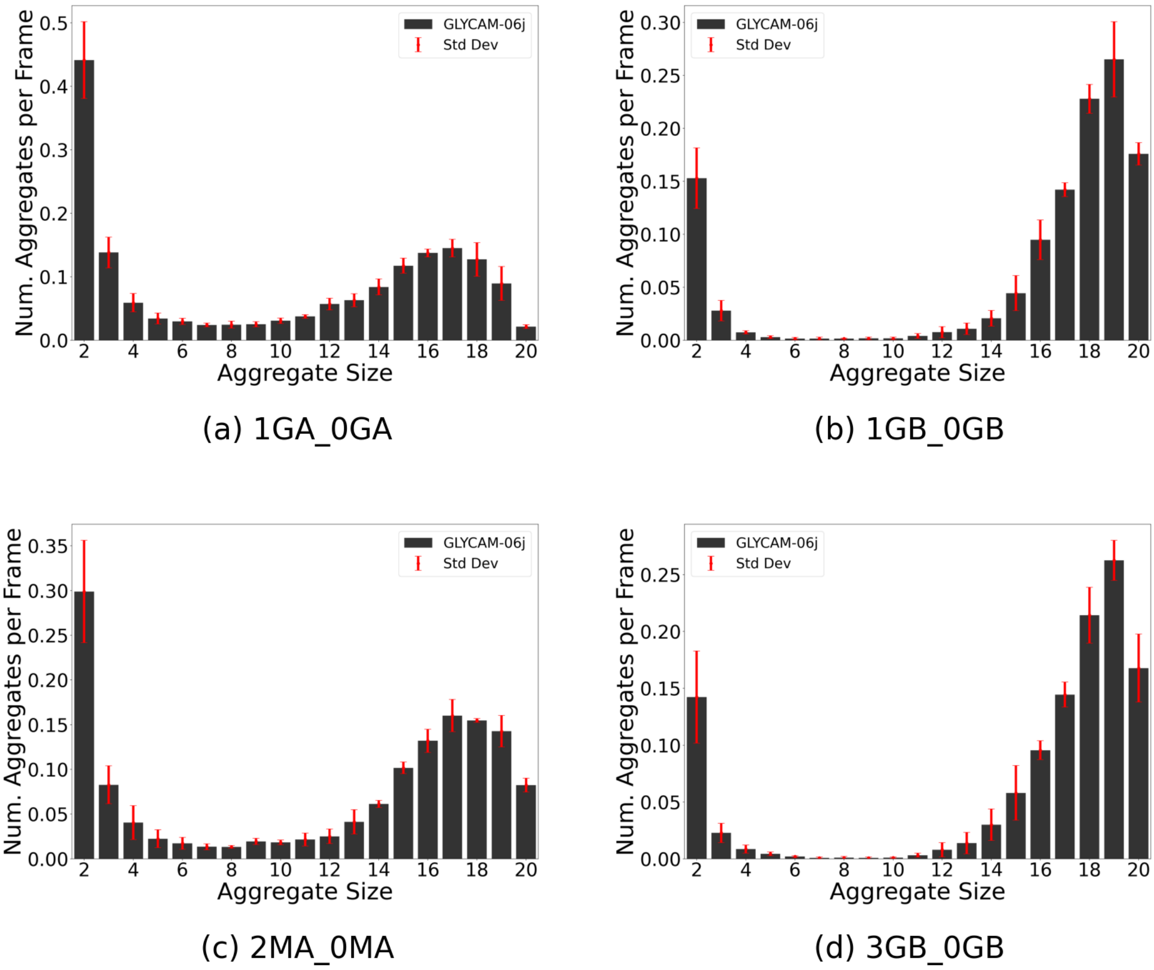

Figure S29: Aggregation data from extension of the Glycam-06j trajectories to 2 microseconds Standard deviations were calculated by performing an equivalent analysis on independent 500 ns chunks of each extended simulation**.** The standard deviations indicate that the overall profile of each aggregate distribution remained similar throughout each 500 ns chunk of the extended simulations.
