# Supplementary material for: Systematic Approach to Parametrization of Disaccharides for the Martini 3 Coarse-Grained Force Field
Source: J Chem Inf Model. 2025 Jan 17;65(3):1537–48. doi: 10.1021/acs.jcim.4c01874 (PMC11815824; doi:10.1021/acs.jcim.4c01874)
Supplement: Supplementary file 2 — ci4c01874_si_002.pdf [file ci4c01874_si_002.pdf]

Prefix	SMILES
1GA_0GA	O([C@@H]1[C@@H]([C@H]([C@@H]([C@@H](CO)O1)O)O)O)[C@@H]1[C@@H]([C@H]([C@@H]([C@@H](CO)O1)O)O)O
1GA_0GB	O([C@@H]1[C@@H]([C@H]([C@@H]([C@@H](CO)O1)O)O)O)[C@@H]1O[C@H](CO)[C@@H](O)[C@H](O)[C@H]1O
1GB_0GA	O([C@@H]1O[C@H](CO)[C@@H](O)[C@H](O)[C@H]1O)[C@@H]1[C@@H]([C@H]([C@@H]([C@@H](CO)O1)O)O)O
1GB_0GB	O([C@@H]1O[C@H](CO)[C@@H](O)[C@H](O)[C@H]1O)[C@@H]1O[C@H](CO)[C@@H](O)[C@H](O)[C@H]1O
2GA_0GA	O[C@@H]1[C@@H]([C@H]([C@@H]([C@@H](CO)O1)O)O)O[C@H]([C@@H]([C@H]([C@@H](CCO)O)O)O)O
2GA_0GB	O[C@@H]1[C@@H]([C@H]([C@@H]([C@@H](CO)O1)O)O)O[C@@H]1O[C@H](CO)[C@@H](O)[C@H](O)[C@H]1O
2GB_0GA	O[C@@H]1O[C@H](CO)[C@@H](O)[C@H](O)[C@H]1O[C@@H]1[C@@H]([C@H]([C@@H]([C@@H](CO)O1)O)O)O
2GB_0GB	O[C@@H]1O[C@H](CO)[C@@H](O)[C@H](O)[C@H]1O[C@@H]1O[C@H](CO)[C@@H](O)[C@H](O)[C@H]1O
3GA_0GA	O[C@@H]1[C@H](O)[C@H]([C@@H]([C@@H](CO)O1)O)O[C@@H]1[C@@H]([C@H]([C@@H]([C@@H](CO)O1)O)O)O
3GA_0GB	O[C@@H]1[C@H](O)[C@H]([C@@H]([C@@H](CO)O1)O)O[C@H](O)[C@@H]([C@H]([C@@H](CCO)O)O)O
3GB_0GA	O[C@@H]1O[C@H](CO)[C@@H](O)[C@@H]([C@H]1O)O[C@@H]1[C@@H]([C@H]([C@@H]([C@@H](CO)O1)O)O)O
3GB_0GB	O[C@@H]1O[C@H](CO)[C@@H](O)[C@@H]([C@H]1O)O[C@@H]1O[C@H](CO)[C@@H](O)[C@H](O)[C@H]1O
4GA_0GA	O[C@@H]1[C@H](O)[C@@H](O)[C@@H]([C@@H](CO)O1)O[C@H]([C@@H]([C@H]([C@@H](CC)O)O)O)O.O
4GA_0GB	O[C@@H]1[C@H](O)[C@@H](O)[C@@H]([C@@H](CO)O1)O[C@@H]1O[C@H](CO)[C@@H](O)[C@H](O)[C@H]1O
4GB_0GA	O[C@@H]1O[C@H](CO)[C@H]([C@H](O)[C@H]1O)O[C@@H]1[C@@H]([C@H]([C@@H]([C@@H](CO)O1)O)O)O
4GB_0GB	O[C@@H]1O[C@H](CO)[C@H]([C@H](O)[C@H]1O)O[C@@H]1O[C@H](CO)[C@@H](O)[C@H](O)[C@H]1O
6GA_0GA	O[C@@H]1[C@H](O)[C@@H](O)[C@H](O)[C@H](O1)CO[C@@H]1[C@@H]([C@H]([C@@H]([C@@H](CO)O1)O)O)O
6GA_0GB	O[C@@H]1[C@H](O)[C@@H](O)[C@H](O)[C@H](O1)CO[C@@H]1O[C@H](CO)[C@@H](O)[C@H](O)[C@H]1O
6GB_0GA	O[C@@H]1O[C@@H]([C@@H](O)[C@H](O)[C@H]1O)CO[C@@H]1[C@@H]([C@H]([C@@H]([C@@H](CO)O1)O)O)O
6GB_0GB	O[C@@H]1O[C@@H]([C@@H](O)[C@H](O)[C@H]1O)CO[C@@H]1O[C@H](CO)[C@@H](O)[C@H](O)[C@H]1O
1MA_0MA	O([C@@H]1[C@H]([C@H]([C@@H]([C@@H](CO)O1)O)O)O)[C@@H]1[C@H]([C@H]([C@@H]([C@@H](CO)O1)O)O)O
1MA_0MB	O([C@@H]1[C@H]([C@H]([C@@H]([C@@H](CO)O1)O)O)O)[C@@H]1O[C@H](CO)[C@@H](O)[C@H](O)[C@@H]1O
1MB_0MA	O([C@@H]1O[C@H](CO)[C@@H](O)[C@H](O)[C@@H]1O)[C@@H]1[C@H]([C@H]([C@@H]([C@@H](CO)O1)O)O)O
1MB_0MB	O([C@@H]1O[C@H](CO)[C@@H](O)[C@H](O)[C@@H]1O)[C@@H]1O[C@H](CO)[C@@H](O)[C@H](O)[C@@H]1O
2MA_0MA	O[C@@H]1[C@H]([C@H]([C@@H]([C@@H](CO)O1)O)O)O[C@@H]1[C@H]([C@H]([C@@H]([C@@H](CO)O1)O)O)O
2MA_0MB	O[C@@H]1[C@H]([C@H]([C@@H]([C@@H](CO)O1)O)O)O[C@@H]1O[C@H](CO)[C@@H](O)[C@H](O)[C@@H]1O
2MB_0MA	O[C@@H]1O[C@H](CO)[C@@H](O)[C@H](O)[C@@H]1O[C@@H]1[C@H]([C@H]([C@@H]([C@@H](CO)O1)O)O)O
2MB_0MB	O[C@@H]1O[C@H](CO)[C@@H](O)[C@H](O)[C@@H]1O[C@@H]1O[C@H](CO)[C@@H](O)[C@H](O)[C@@H]1O
3MA_0MA	O[C@@H]1[C@@H](O)[C@H]([C@@H]([C@@H](CO)O1)O)O[C@@H]1[C@H]([C@H]([C@@H]([C@@H](CO)O1)O)O)O
3MA_0MB	O[C@@H]1[C@@H](O)[C@H]([C@@H]([C@@H](CO)O1)O)O[C@H](O)[C@H]([C@H]([C@@H](CCO)O)O)O
3MB_0MA	O[C@@H]1O[C@H](CO)[C@@H](O)[C@@H]([C@@H]1O)O[C@@H]1[C@H]([C@H]([C@@H]([C@@H](CO)O1)O)O)O
3MB_0MB	O[C@@H]1O[C@H](CO)[C@@H](O)[C@@H]([C@@H]1O)O[C@@H]1O[C@H](CO)[C@@H](O)[C@H](O)[C@@H]1O
4MA_0MA	O[C@@H]1[C@@H](O)[C@@H](O)[C@@H]([C@@H](C)O1)O[C@H]([C@H]([C@H]([C@@H](CC)O)O)O)O.O.O
4MA_0MB	O[C@@H]1[C@@H](O)[C@@H](O)[C@@H]([C@@H](CO)O1)O[C@@H]1O[C@H](CO)[C@@H](O)[C@H](O)[C@@H]1O
4MB_0MA	O[C@@H]1O[C@H](CO)[C@H]([C@H](O)[C@@H]1O)O[C@@H]1[C@H]([C@H]([C@@H]([C@@H](CO)O1)O)O)O
4MB_0MB	O[C@@H]1O[C@H](CO)[C@H]([C@H](O)[C@@H]1O)O[C@H](O)[C@H]([C@H]([C@@H](CCO)O)O)O
6MA_0MA	O[C@@H]1[C@@H](O)[C@@H](O)[C@H](O)[C@H](O1)CO[C@@H]1[C@H]([C@H]([C@@H]([C@@H](CO)O1)O)O)O
6MA_0MB	O[C@@H]1[C@@H](O)[C@@H](O)[C@H](O)[C@H](O1)CO[C@@H]1O[C@H](CO)[C@@H](O)[C@H](O)[C@@H]1O
6MB_0MA	O[C@@H]1O[C@@H]([C@@H](O)[C@H](O)[C@@H]1O)CO[C@@H]1[C@H]([C@H]([C@@H]([C@@H](CO)O1)O)O)O
6MB_0MB	O[C@@H]1O[C@@H]([C@@H](O)[C@H](O)[C@@H]1O)CO[C@@H]1O[C@H](CO)[C@@H](O)[C@H](O)[C@@H]1O
